# Development of Biocompatible Cu(I)‐Microdevices for Bioorthogonal Uncaging and Click Reactions

**DOI:** 10.1002/chem.202400611

**Published:** 2024-04-16

**Authors:** Melissa van de L'Isle, Stephen Croke, Teresa Valero, Asier Unciti‐Broceta

**Affiliations:** ^1^ Edinburgh Cancer Research Institute of Genetics & Cancer University of Edinburgh Crewe Road South Edinburgh EH4 2XR UK; ^2^ Department of Medicinal & Organic Chemistry and Excellence Research Unit of Chemistry applied to Biomedicine and the Environment Faculty of Pharmacy University of Granada Campus de Cartuja s/n 18071 Granada Spain; ^3^ GENYO Centre for Genomics and Oncological Research Pfizer/University of Granada/Andalusian Regional Government Avda. Ilustración 114 18016 Granada Spain; ^4^ Instituto de Investigación Biosanitaria ibs.GRANADA Granada Spain

## Abstract

Transition‐metal‐catalyzed bioorthogonal reactions emerged a decade ago as a novel strategy to implement spatiotemporal control over enzymatic functions and pharmacological interventions. The use of this methodology in experimental therapy is driven by the ambition of improving the tolerability and PK properties of clinically‐used therapeutic agents. The preclinical potential of bioorthogonal catalysis has been validated *in vitro* and *in vivo* with the *in situ* generation of a broad range of drugs, including cytotoxic agents, anti‐inflammatory drugs and anxiolytics. In this article, we report our investigations towards the preparation of solid‐supported Cu(I)‐microdevices and their application in bioorthogonal uncaging and click reactions. A range of ligand‐functionalized polymeric devices and off‐on Cu(I)‐sensitive sensors were developed and tested under conditions compatible with life. Last, we present a preliminary exploration of their use for the synthesis of PROTACs through CuAAC assembly of two heterofunctional mating units.

## Graphical abstract

Here we report the development of solid‐supported Cu(I) catalysts and their application in bioorthogonal uncaging and click reactions. Polymeric devices featuring a range of tridentate ligands and off‐on Cu(I)‐sensitive sensors were developed and tested under conditions compatible with life. We also present a preliminary exploration of their use for in situ PROTAC synthesis through CuAAC assembly of two heterofunctional mating units.

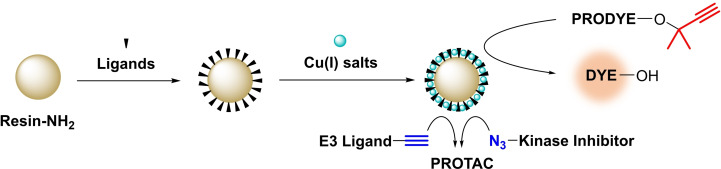

## Introduction

The use of abiotic metal catalysts to regulate cell functions in a bioorthogonal fashion was introduced over a decade ago.[^1^, ^2^] Li *et al*. employed Pd(II) complexes for the intracellular activation of a bacterial lyase whose catalytic lysine residue had been modified with a propargyloxycarbonyl (PoC) group,^[1]^ whereas Weiss and coworkers developed a *N*1‐propargyl derivative of the cytotoxic drug 5FU that was harmless to cells and selectively uncaged in a locally‐controlled manner by Pd(0)‐functionalized implants.^[2]^ Notably, taking different paths, both labs demonstrated that the propargyl group was highly sensitive to Pd‐mediated uncaging, which ended up replacing the previously used allyl‐based groups[^3^, ^4^] as the masking group of choice for the design of Pd‐activatable probes and bioactive agents.[^5^, ^6^, ^7^, ^8^] These and other seminal works[^9^, ^10^, ^11^, ^12^, ^13^, ^14^] sparked the growth of this emergent sub‐area of bioorthogonal chemistry, catalyzing the development of numerous prodrug strategies,[^15^, ^16^, ^17^, ^18^, ^19^, ^20^, ^21^, ^22^, ^23^, ^24^, ^25^, ^26^, ^27^, ^28^] novel biomedical applications[^29^, ^30^, ^31^, ^32^, ^33^, ^34^, ^35^, ^36^, ^37^, ^38^, ^39^, ^40^, ^41^] and the exploration of additional transition metals, including Ru,[^11^, ^23^] Pt,[^42^, ^43^] Au,[^44^, ^45^] Fe[^37^, ^38^] and Cu.[^16^, ^46^, ^47^, ^48^]

**Figure 1:**
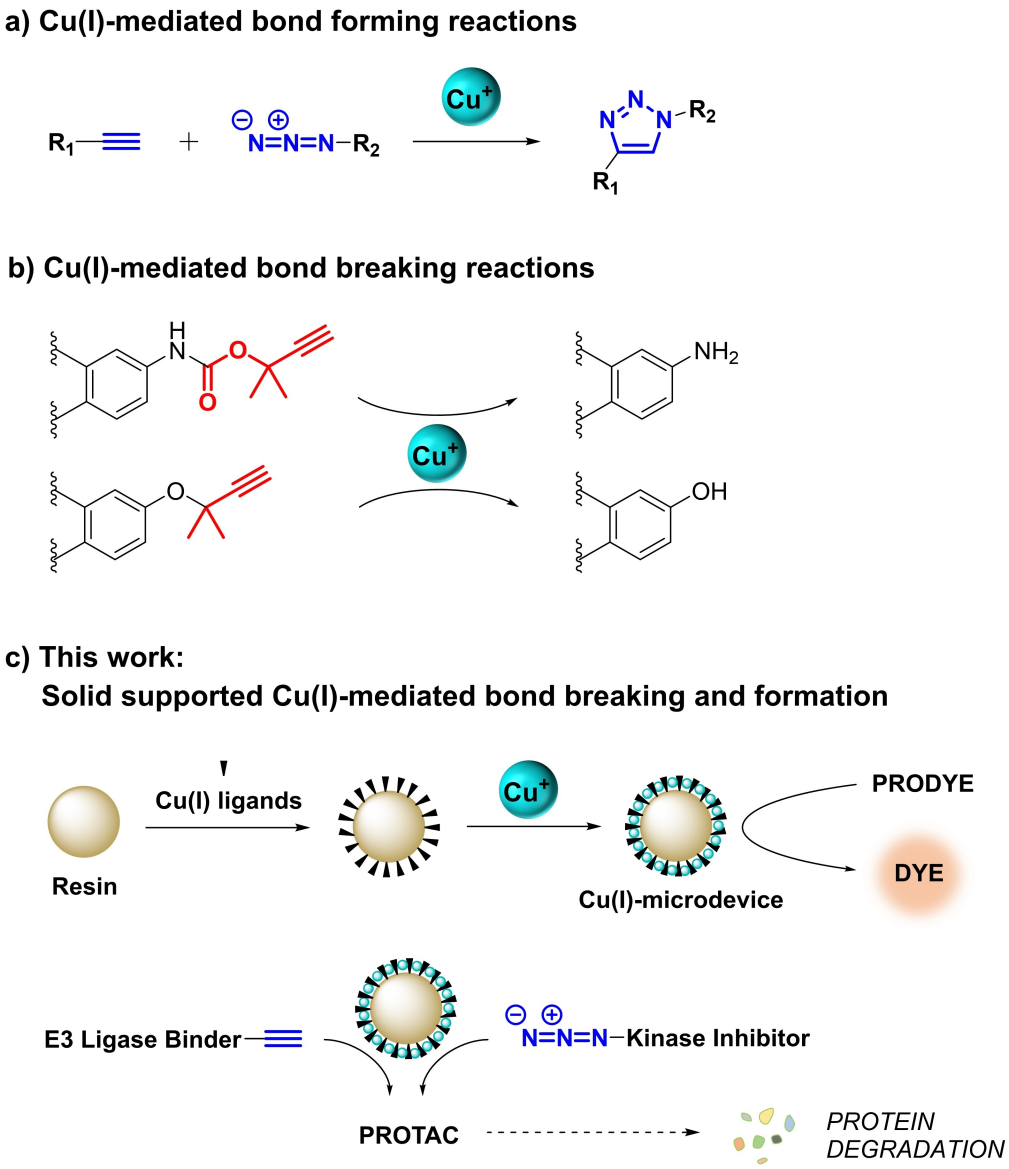
Previous homogeneous Cu(I)‐mediated bond‐forming and bond‐breaking reactions explored in bioorthogonal catalysis (a,b) and current work (c).

Numerous studies have contributed to expand the scope of alkynes as reactants and substrates for different metals, including Cu. Although Cu(II) is the physiological oxidation state of this transition metal in cells and organisms, several research groups have demonstrated the compatibility of Cu(I)‐mediated catalysis to living environments.[^46^, ^47^, ^48^, ^49^, ^50^, ^51^, ^52^] The alkynophilic properties of Cu(I) have not only found application in assisting azide‐alkyne cycloadditions (CuAAC, see Figure 1a) in cells,[^46^, ^47^, ^50^, ^51^, ^52^] but can also be used to cleave O−C bonds when the corresponding alkynyloxy moiety features its triple bond at the β‐position and double methylation at the α‐position (Figure 1b). Albertazzi and Palmans,^[48]^ and Chen^[49]^ showed that *O*‐(1,1‐dimethylpropargyl) groups are particularly labile to Cu(I) in the presence of suitable ligands and sodium ascorbate. As a result, both bond‐forming and bond‐breaking reactions have been explored in bioorthogonal catalysis for the synthesis of ‘clickable’ drugs and the uncaging of ‘cleavable’ probes, respectively.

Inspired by these investigations, here we report the development and bioorthogonal use of solid‐supported Cu(I) catalysts to mediate bond‐breaking and bond‐forming reactions (Figure 1c). We demonstrate that these extracellular catalytic devices are tolerated by cells and capable of uncaging masked dyes under physiological conditions. Finally, we also explore the assembly of proteolysis‐targeting chimeras via *in situ* CuAAC chemistry as a novel strategy to induce combined spatially‐ and molecularly‐targeted protein degradation.

## Results and Discussion

**Scheme 1:**
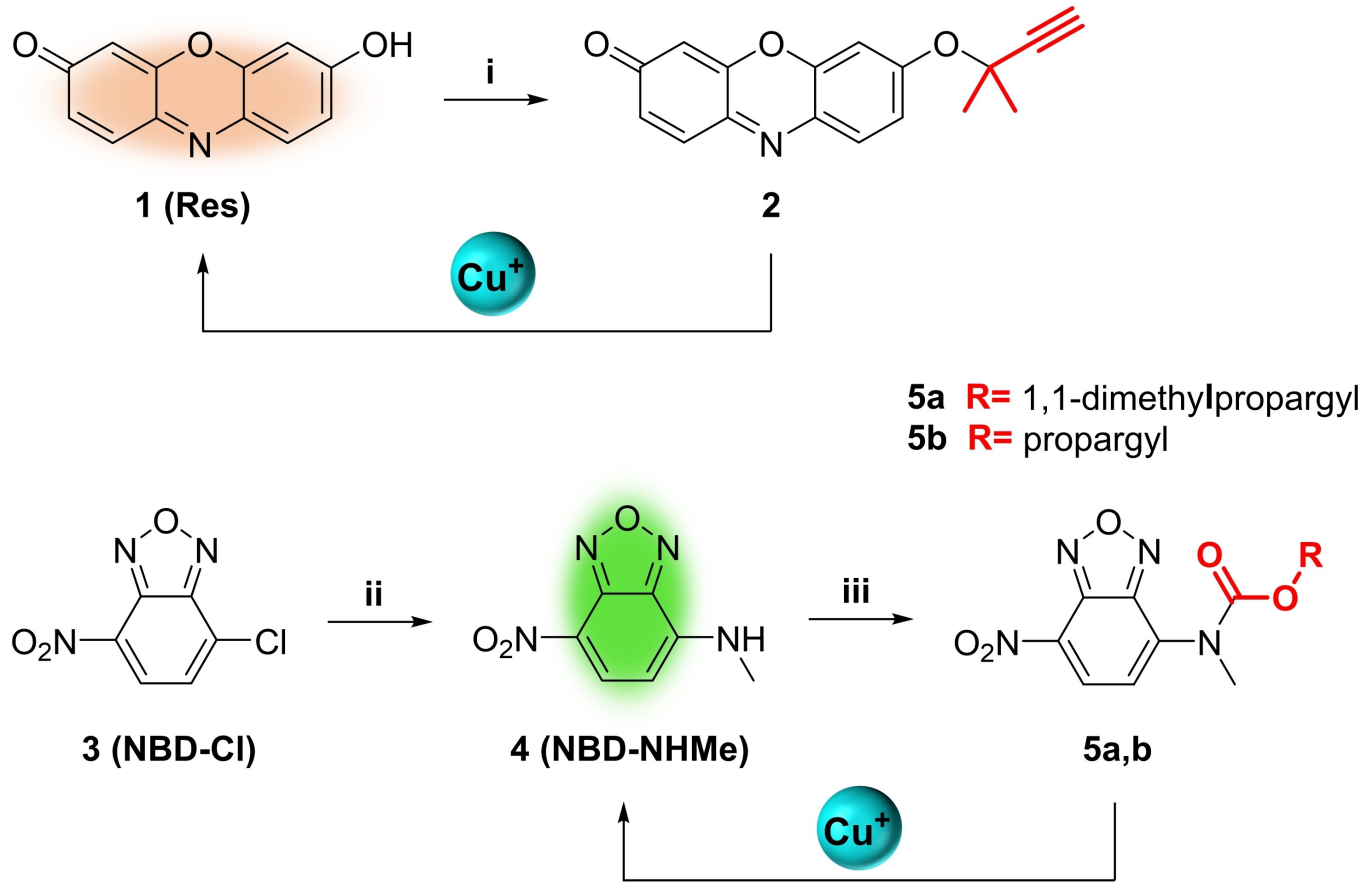
Synthesis of resorufin‐based prodye **2** and NBD‐based prodyes **5 a,b**. Reaction conditions: (i) DBU, 3‐chloro‐3‐methylbut‐1‐yne, CuCl_2_, MeCN, rt, 4 h, 15 %; (ii) methylamine, EtOH, rt, 30 min., 82 %; (iii) Et_3_N, 4‐nitrophenol‐1,1‐dimethylpropargyloxycarbonate or propargyl chloroformate, DMF, rt, overnight (47 % and 33 % for **5 a** and **5 b**, respectively).

**Preparation and preliminary screening of Cu(I)‐activatable prodyes for fluorimetric studies**. To confirm and rank the reactivity of Cu catalysts, two types of off‐on fluorescent sensors were developed. Scheme 1 describes the synthesis routes for the preparation of novel caged analogues of resorufin (**1**, **Res**) and nitrobenzoxadiazole ‐methylamine (**4**, NBD‐NHMe). To achieve selective sensitivity to Cu(I), dimethyl‐substituted propargyl handles were incorporated into key functional groups required for the fluorescent properties of the dyes. Prodye **2** was prepared in a single step by reacting **Res** with 3‐chloro‐3‐methylbut‐1‐yne in the presence of DBU and copper (II) chloride (CuCl_2_) in MeCN. Synthesis of prodye **5 a** was carried out in two steps: reaction of NBD−Cl, **3**, and methylamine in DMF followed by carbamate formation with 4‐nitrophenol‐1,1‐dimethylpropargyloxycarbonate. Propargyloxycarbonyl‐protected prodye **5 b** was prepared as a non‐Cu(I)‐cleavable control, since this masking group have been found to be resistant to Cu(I)‐mediated cleavage.[^48^, ^49^]

**Figure 2:**
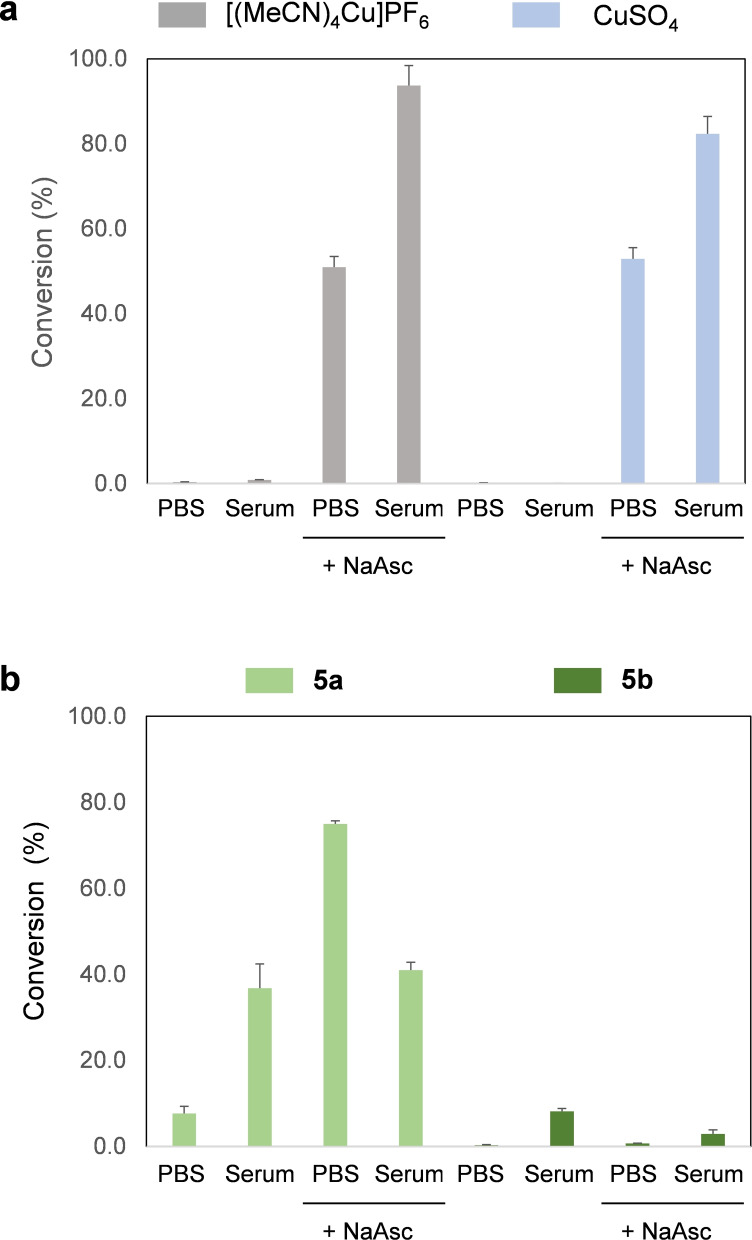
Fluorogenic analysis of the capacity of Cu salts to uncage prodyes **2** and **5 a,b** for 2 h under different conditions. Fluorescence detection of **Res** with PerkinElmer 2101 Envision Multilabel Plate Reader (λ_ex_=550 nm/λ_em_=580 nm) and detection of **4** with Tecan Spark 20 M Plate Reader (λ_ex_=485 nm/λ_em_=530 nm).

To validate the sensitivity of the sensors to Cu, pro‐fluorophore **2** was incubated with two different Cu salts: [(MeCN)_4_Cu]PF_6_ and CuSO_4_.5H_2_O. Reactions were performed at 37°C in a thermomixer (750 rpm) in PBS only or supplemented with 10 % serum (FBS), and in the absence or presence of sodium ascorbate (NaAsc). Fluorescence analysis (λex/em=485/535 nm) determined that **2** was only cleaved by copper salts in the presence of the natural antioxidant NaAsc (see Figure 2), which generates Cu(I) *in situ*. Interestingly, both [(MeCN)_4_Cu]PF_6_ and CuSO_4_ showed higher capacity to convert **2** into **Res** in the presence of serum proteins (10 % FBS). Over 90 % conversion in the presence of serum took place in 2 h. On the other hand, uncaging of NBD‐based prodye **5 a** with [(MeCN)_4_Cu]PF_6_ also required NaAsc, although the reaction proceeded at slower rate in the presence of serum. As expected, incubation with Poc‐protected prodye **5 b** resulted in low levels of fluorescence emission. Based on these results, **Res**‐based off‐on sensor **2** was selected for testing the catalytic properties of the Cu(I)‐microdevices.

**Synthesis of appendable Cu(I) tridentate ligands**. Tridentate 1,2,3‐triazole‐based ligands display remarkable capacities to accelerate Cu(I)‐mediated reactions in aqueous media.^[53]^ Mascareñas proved that these ligands are capable of facilitating CuAAC reactions inside human cells,^[47]^ which was also confirmed for Cu(I)‐mediated uncaging reactions by Chen.^[49]^ Motivated by these works, we envisaged the incorporation of this kind of ligands onto the surface of biocompatible resins to coordinate and trap Cu(I) ions at the outer surface of a solid support, and thereby generate so‐called Cu(I)‐microdevices capable of eliciting bioorthogonal Cu(I)‐catalyzed reactions with spatial control, i. e. at the location of the catalytic implant.

**Scheme 2:**
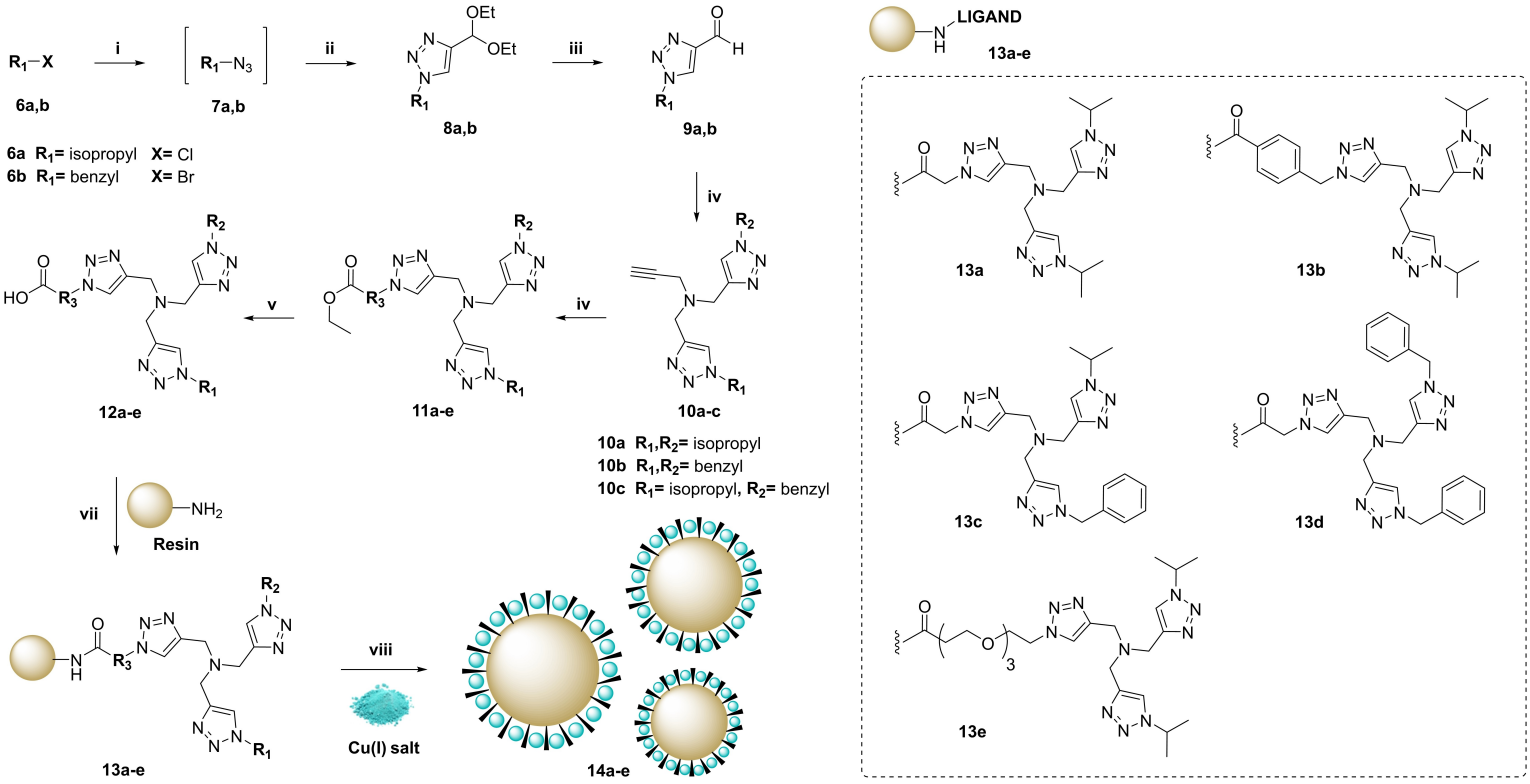
General synthesis of Cu(I) tridentate ligands and preparation of solid‐supported Cu(I) catalysts. The library of ligands employed for Tentagel resin functionalization is shown in the box. Reaction conditions: (i) NaN_3_, NaHCO_3_, *t*‐BuOH/H_2_O, rt, 10 min.; (ii) 3,3‐diethoxy‐but‐1‐yne, CuSO_4_, NaAsc, 70 °C, 24 h; (iii) DCE/ H_2_O, TFA, 3–9 h, over 2‐steps 86 % and 92 % for **9 a** and **9 b**, respectively; (iv) propargyl amine, sodium triacetoxyborohydride, DCM, rt, 2–4 h, 10a 70 %, 10b 31 %, 10c 24 %; (v) ethyl azidoacetate or N_3_‐R_3_, Cu(OAc)_2_, NaAsc, MeCN, rt, overnight; (vi) KOH, MeOH, rt, overnight; (vii) Tentagel‐NH_2_ resin, DIPEA, oxyma, DIC, DMF, rt, overnight; (viii) [(MeCN)_4_Cu]PF_6_, then NaAsc, MeOH, rt, 2 min vortex.

First, we prepared a library of tridentate triazole‐based ligands featuring a carboxyl group in one of the rings to facilitate covalent attachment to the resins via amide coupling. The synthesis of the Cu‐chelator library is described in Scheme 2. One‐pot reaction of alkyl halides **6 a,b** with sodium azide followed by CuAAC reaction with 3,3‐diethoxy‐1‐propyne generated the corresponding acetal‐protected triazoles **8 a,b**. Aldehyde formation followed by reductive amination gave rise to *N*,*N*‐bis‐(1,2,3‐triazolemethyl)‐N‐propargyl amines **10 a**–**c**. The third triazole ring was formed by CuAAC reaction with ethyl 2‐azidoacetate, ethyl 4‐(azidomethyl)benzoate or ethyl azido‐PEG_3_‐carboxylate to incorporate the precursor of the carboxyl handle into the tridentate ligand. The reaction crudes required purification by semipreparative TLC to obtain the pure tridentate ligands **11 a**–**f**. Saponification with potassium hydroxide produced the corresponding carboxylic acids **12 a**–**f** without further purification.

**Preparation and characterization of Cu(I)‐microdevices**. Amino‐functionalized PEG‐grafted polystyrene resins (Rapp Polymere) of 75 μm in diameter were used to prepare the catalytic devices, since they have shown high biocompatibility *in vitro* and *in vivo* with metal nanoparticles trapped in their polymeric structure.[^19^, ^28^, ^41^] Of note, these resins are larger than cells and therefore remain in the extracellular environment, avoiding intracellular catalyst deactivation. The ligands were coupled to the resins using oxyma/DIC and, subsequently, treated with [(MeCN)_4_Cu]PF_6_ and NaAsc at room temperature for 2 min to facilitate the coordination of Cu(I) ions to the resins. The Cu content of each of the Cu(I)‐microdevices generated was characterized by ICP‐MS, demonstrating that the efficacy of metal ion loading was influenced by the nature of the ligand. Freshly prepared Cu(I)‐microdevices were then treated with prodye **2** in the absence and presence of serum. Interestingly, while Cu‐microdevices containing more lipophilic phenyl‐rich ligands (**14 b**, **14 c** and **14 d**) led to higher Cu‐to‐ligand ratios, the best performing catalytic devices did not possess ligands with phenyl rings (**14 a** and **14 e**). **14 a**, which features two isopropyl groups and a short linking spacer, led to the most favorable catalytic properties (see Table 1). All the catalytic devices generated the fluorescent dye **Res**, although Cu(I)‐microdevices containing the ligand **14 a** showed superior catalytic properties in serum with 83 % conversion in 15 min. The increase of Cu(I) reaction rates by the ligand‐functionalized resins compared to free copper salts (see Figure 2a) is noteworthy.

**Table 1:** Chemical and functional characterization of Cu(I)‐microdevices.

Ligand/Resin size	Est. maximum loading capacity (μmol/mg)^*a*^	Exp. Cu‐to‐ligand loading ratio avg. (%)^*a*^	2‐to‐Res conversion in PBS (%)^*b*^	2‐to‐Res conversion in PBS+FBS (%)^*b*^
**14 a/75 μm**	0.391	36	42	83
**14 b/75 μm**	0.380	76	31	81
**14 c/75 μm**	0.384	64	38	20
**14 d/75 μm**	0.377	55	31	65
**14 e/75 μm**	0.370	32	32	72

^*a*^ The ICP‐MS samples were prepared by leaching the Cu(I)‐microdevices with a 3 : 1 HCl:HNO_3_ solution and dilution to 2.5 % v/v acid/H_2_O sample solution. The copper content per microdevice was determined by calculating the maximal ligand loading and estimating the maximal copper coordination value at a 1 : 1 ratio. ^*b*^ The uncaging percentage of 25 μM prodye **2** into **Res** by 0.5 mg Cu(I)‐microdevices (**13 a**–**e**) was determined in PBS and serum, in the presence of an excess of NaAsc. Data in the table shows uncaging percentages after 15 min reactions.

Additionally, to study the influence of the resin size and the copper salt, the isopropyl‐triazole‐based ligand **12 a** was coupled to resins of 10, 30 and 75 μm in average diameter (0.21, 0.26 and 0.46 mmol/g of loading capacity, respectively) and loaded with tetrakis(acetonitrile)copper(I) tetrafluoroborate ([(MeCN)_4_Cu]BF_4_) followed by NaAsc treatment. The variation of the ratio of surface amino groups was addressed to study the most favourable ligand density. Commercial TentaGel™ TBTA resins (Sigma Aldrich, 75 μm, 0.17 mmol/g of loading capacity) were acquired and treated with the Cu(I) salt under the same reaction conditions to compare with the novel library of Cu(I)‐microdevices. ICP‐MS analysis showed that the superior loading capacity of the 75 μm resins led to an increment of Cu content. Importantly, fluorimetric assays determined that the isopropyl functionalized 75 μm resin showed to be the most favourable ligand:resin ratio and exhibited the highest catalytic activity, being superior to the commercial resin and the resins of smaller sizes (see Table S1 and Figure S1, Supp. Inf.). Therefore, **14 a** was selected as the Cu(I)‐microdevices for subsequent studies (named Cu(I)‐microdevices from now on).

**Figure 3:**
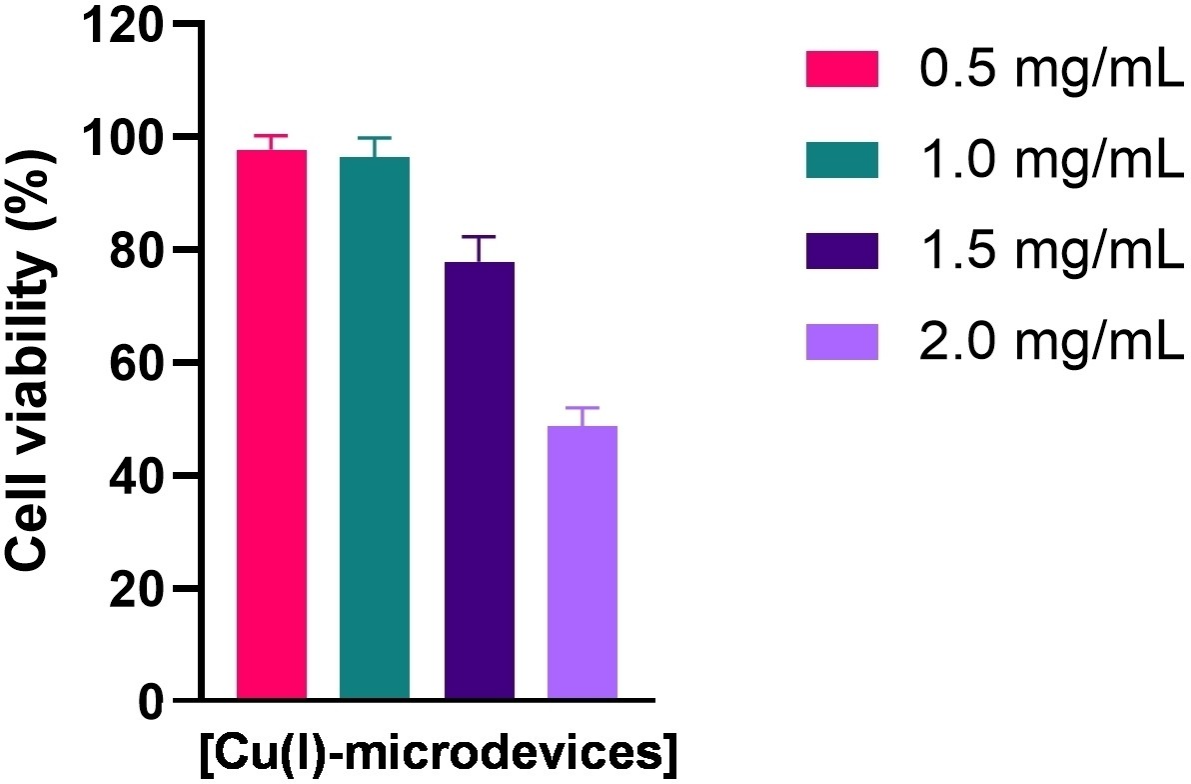
Biocompatibility study for Cu(I)‐microdevices. MDA‐MB‐231 cells were incubated for 5 d with 0.1, 0.5, 1, 1.5 or 2 mg/mL of Cu(I)‐microdevices and cell viability determined with PrestoBlue. Error bars: ±SD from n=3.

**Tolerability of Cu(I)‐microdevices in cell culture**. Even though Cu(II) is an essential cofactor for a variety of physiological processes,^[54]^ it is cytotoxic in excess due to its potential to generate reactive oxygen species in the cytoplasm.^[55]^ Although Cu(I) is considered to be less harmful to cells, its toxicity cannot be underestimated in certain conditions.^[56]^ Our drive to trap Cu(I) on the surface of ‘extracellular’ devices aimed to explore the bioorthogonal potential of Cu(I), with two fundamental objectives: (i) to achieve control over the location where Cu(I) reactions occur; and (ii) to reduce cell entry and copper‐mediated toxicities. As a first step, we tested the tolerability of human cells to the catalytic devices by treating the breast cancer MDA‐MB‐231 cells with a range of concentrations of Cu(I)‐microdevices (0.1, 0.5, 1, 1.5 and 2 mg/mL). After 5 d of incubation, cell viability was determined using the PrestoBlue assay. As shown in Figure 3, a slight reduction of cell viability was observed at 1.5 mg/mL. Importantly, cells exhibited high tolerability to the presence of Cu(I)‐microdevices at a concentration of 1 mg/mL or lower. Of note, prior fluorogenic studies were performed at 0.5 mg/mL. To test the devices at their maximum reactivity potential, 1 mg/mL was chosen as the optimal concentration of Cu(I)‐microdevices for cell culture experimentation.

**Development of Cu(I)‐assemblable proteolysis‐targeting chimeras (PROTACs)**. Our lab has significant interest in understanding the role of kinases in different malignancies and seeks for novel ways to inhibit or abolish kinase activity for cancer treatment. One of the main concerns of kinase inhibitors is their promiscuity, which results in moderate to severe side effects.^[57]^ In fact, numerous clinically‐tested kinase inhibitors that display highly‐potent anticancer activities in preclinical models have not yet fulfilled their medical potential because of off‐target toxicities. Nonetheless, such off‐target activities can in many cases enhance anticancer potency.^[58]^ Therefore, harnessing the antitumor potential of these agents by controlling where and when they elicit their activity could boost their clinical use.

**Figure 4:**
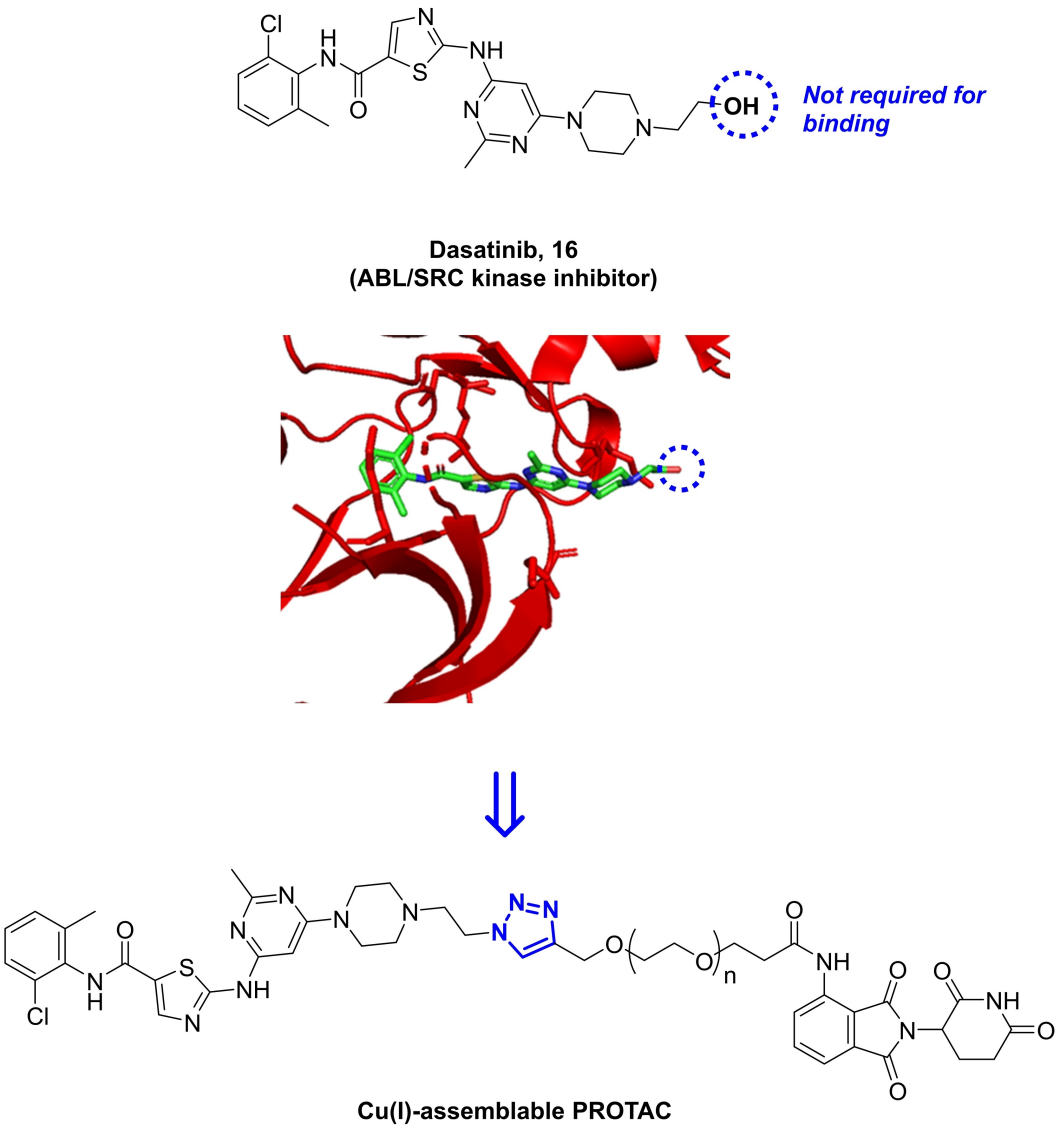
Strategies for the development of a Cu(I)‐assemblable dasatinib‐pomalidomide PROTAC. Co‐crystal structure of dasatinib bound to SRC kinase domains (PDB: 3G5D) shows that the OH group is solvent exposed and does not to interact with the protein.

Capitalizing on the chemical scope offered by Cu(I), a novel strategy was investigated to achieve spatiotemporal disruption of kinase signaling by Cu(I)‐mediated PROTAC assembly to induce spatial and molecular control over kinase degradation (Figure 4). We designed a triazole‐linked PROTAC of the ABL/SRC inhibitor dasatinib. Proteolysis‐targeted chimeras (PROTACs) are heterobifunctional molecules that exploit the capacity of the proteasome system to recognise and degrade proteins that are tagged with poly‐ubiquitin chains. Its modular structure comprises a ligand with high affinity to a specific protein (=degradation target) and a fragment with high binding capacity to an E3‐ligase connected through a linking group. PROTACs bring an E3‐ligase and the protein of choice to close proximity to enforce ubiquitination, thus signaling to proteasomal degradation.[^59^, ^60^] The use of this emerging strategy to ‘disable’ onco‐targets has been validated with multiple E3‐ligase binders and protein ligands. Nonetheless, PROTACs are typically limited by their large size and lipophilicity (>800 Da, LogP>5), which result in DMPK issues. In addition, systemic proteolytic activity and hijacking physiological E3‐ligase activity can cause toxic events in healthy tissues and organs. We envisioned that these limitations could be circumvented by assembling a PROTAC at the site of the disease by solid‐supported CuAAC reaction of two ‘halved’ complementary units. As a proof‐of‐concept, we selected the promiscuous ABL/SRC kinase inhibitor dasatinib and the E3 ligase ligand pomalidomide to design a Cu(I)‐assemblable PROTAC. According to the co‐crystal structure of dasatinib, **16**, in complex with the kinase domain of SRC,^[61]^ its hydroxyl group is solvent exposed and does not establish bonding interactions with the protein (see Figure 4). Consequently, this position is ideal to grow the structure without affecting the binding capacity of the molecule, as shown for different strategies[^62^, ^63^] including PROTACs,^[64]^ and would be critical to develop a potent protein degrader.

**Scheme 3:**
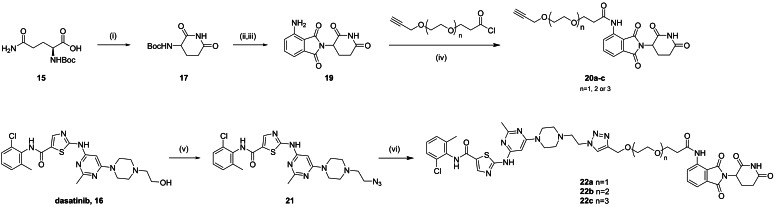
Synthesis of triazole‐linked dasatinib‐pomalidomide conjugates **22 a**–**c**. Reagents and conditions: (i) CDI, DMAP, THF, reflux, 14 h (77 %); (ii) Phthalic anhydride, NaOAc, CH_3_CO_2_H, reflux, 6 h (81 %); (iii) SnCl_2_.2H_2_O, THF, reflux, 1 h (11 %); (iv) alkyne‐PEGn‐acyl chloride, pyridine, DMF, 0 °C‐80 °C, overnight (56‐77 %) (v) MsCl, TEA, DMF, 0 °C‐RT, 3 h then NaN_3_, RT, o/n (70 %); (vi) CuSO_4_, NaAsc, TBTA, H_2_O/DMF, 80 °C, 1 h (70‐86 %).

Following the synthetic steps described in Scheme 3 (see full experimental protocols in the Supp. Inf.), we prepared three different conjugates, **22 a**–**c**, by CuAAC reaction of pomalidomide‐PEGn‐alkynes **20 a**–**c** and dasatinib‐azide **21** in good yields. The addition of the commercial reagent tris[(1‐benzyl‐1H‐1,2,3‐triazol‐4‐yl)methyl]amine (TBTA) increased the yield of the reaction from approx. 15 % (in the absence of TBTA) to over 70 % under the same conditions, which was encouraging for the prospect of our work given the types of ligands used in the microdevices. The use of different PEG spacers aimed to optimize protein degradation, with a “goldilocks” length described whereby the linker length is not too short to result in steric hindrance of the proteins blocking the formation of a ternary complex, or too long whereby multiple ternary complex conformers are possible and ubiquitination is not favored.^[65]^

**Figure 5:**
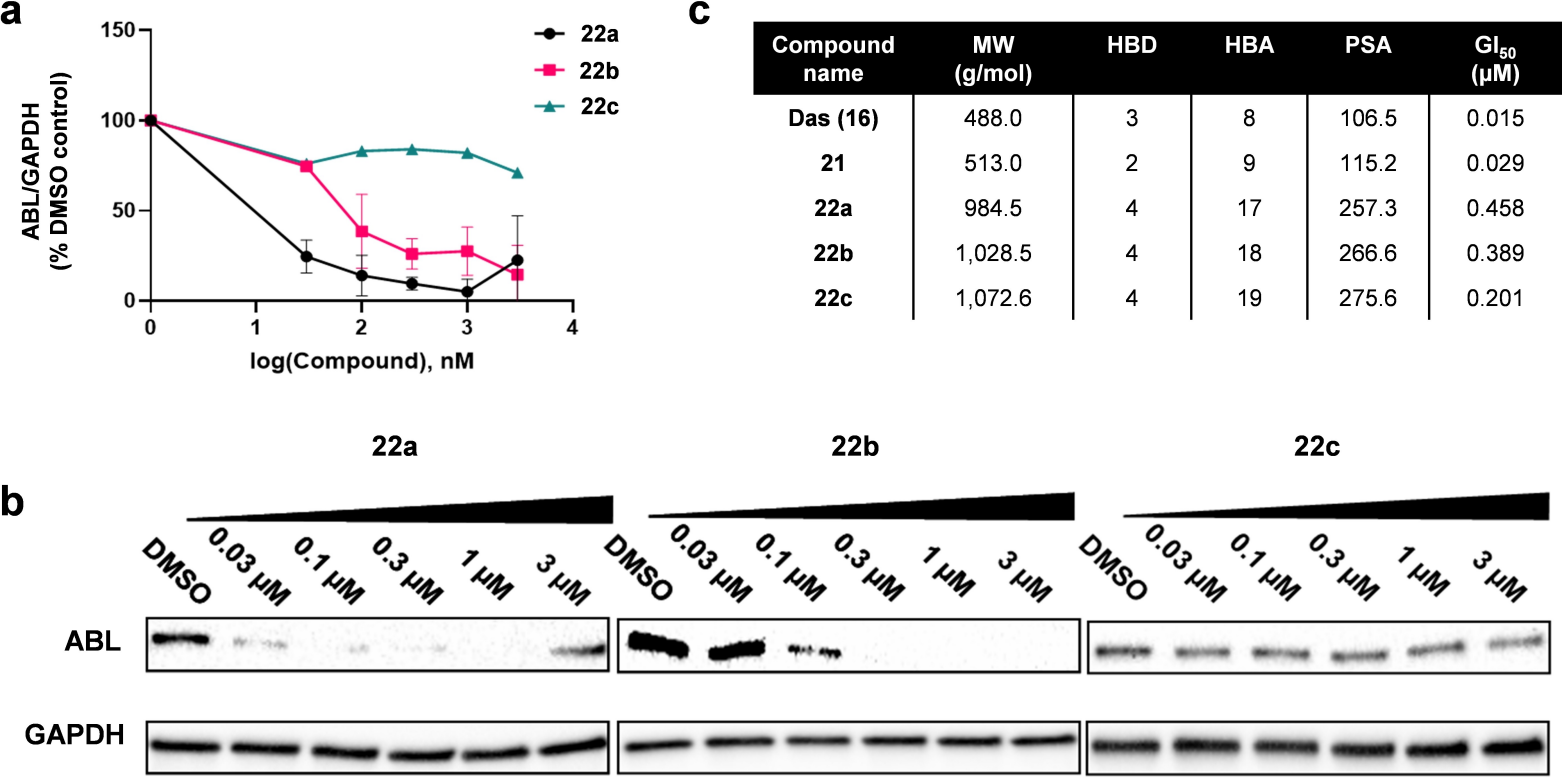
**Protein degradation and antiproliferative activity of 22 a–c in MDA‐MB‐231 cells**. (a) Cells were treated for 24 h prior to cell lysis and analysis by Western blot. ABL kinase levels were normalized to GAPDH and expressed as a % of the ratio of the DMSO control. Graphs show average of two biological replicates with standard error. (b) Representative Western blot of MDA‐MB‐231 cells. (c) GI_50_ values with standard error and structural properties of **22 a**–**c** compared to **16** (dasatinib) and **21** (dasatinib‐azide).

The conjugates were then tested in MDA‐MB‐231 breast cancer cells, which express the nonreceptor tyrosine kinase ABL, and analyzed by Western blot. Cells were treated with **22 a**–**c** for 24 h at a dose range from 0.03 to 3 μM. Cell lysis and immunoblotting are fully described in the Supp. Inf. As shown in Figure 5a,b, **22 a** and **22 b** achieved dose dependent decrease of ABL protein. A trend was evident across the series: the longer the length of the linker, the lower the protein degradation, with **22 c** showing very low levels of protein degradation. The most potent degrader, **22 a**, was able to achieve in excess of 90 % degradation of ABL at lower concentrations than **22 b**. Of note, the highest concentration used for **22 a**, 3 μM, achieved lower protein degradation than treatment with 1 μM. This phenomenon is known as the hook effect,^[60]^ which occurs when elevated intracellular concentrations of PROTACs result in the saturation of the E3 ligase and/or ABL binding site, preventing the formation of the ternary complexed needed for degradation. Interestingly, ABL degradation was inversely proportional to the antiproliferative effect of the PROTAC, indicating that additional effects on other targets (e. g., SRC) are responsible for the full cytotoxic activity of each compound. These results also demonstrate that ABL kinase is not the key oncogenic driver of MDA‐MB‐231 cell proliferation, in agreement with Soellner's observations.^[66]^ A comparative analysis of the different structural properties of dasatinib, azide **21** and the conjugates is also shown in Figure 5c.

Next, since SRC kinase is also targeted by dasatinib, immunoblotting studies were performed to determine the capacity of **22 a**–**c** to degrade this non‐receptor tyrosine kinase (Figure S2, Supporting Information). Interestingly, only **22 a** degraded SRC to some degree, although with substantially lower efficacy than for ABL. This indicates that the structural design of these conjugates is not optimal to form an effective ternary complex to favor SRC ubiquitination. Therefore, ABL was selected as the target of choice for subsequent studies.

**Figure 6:**
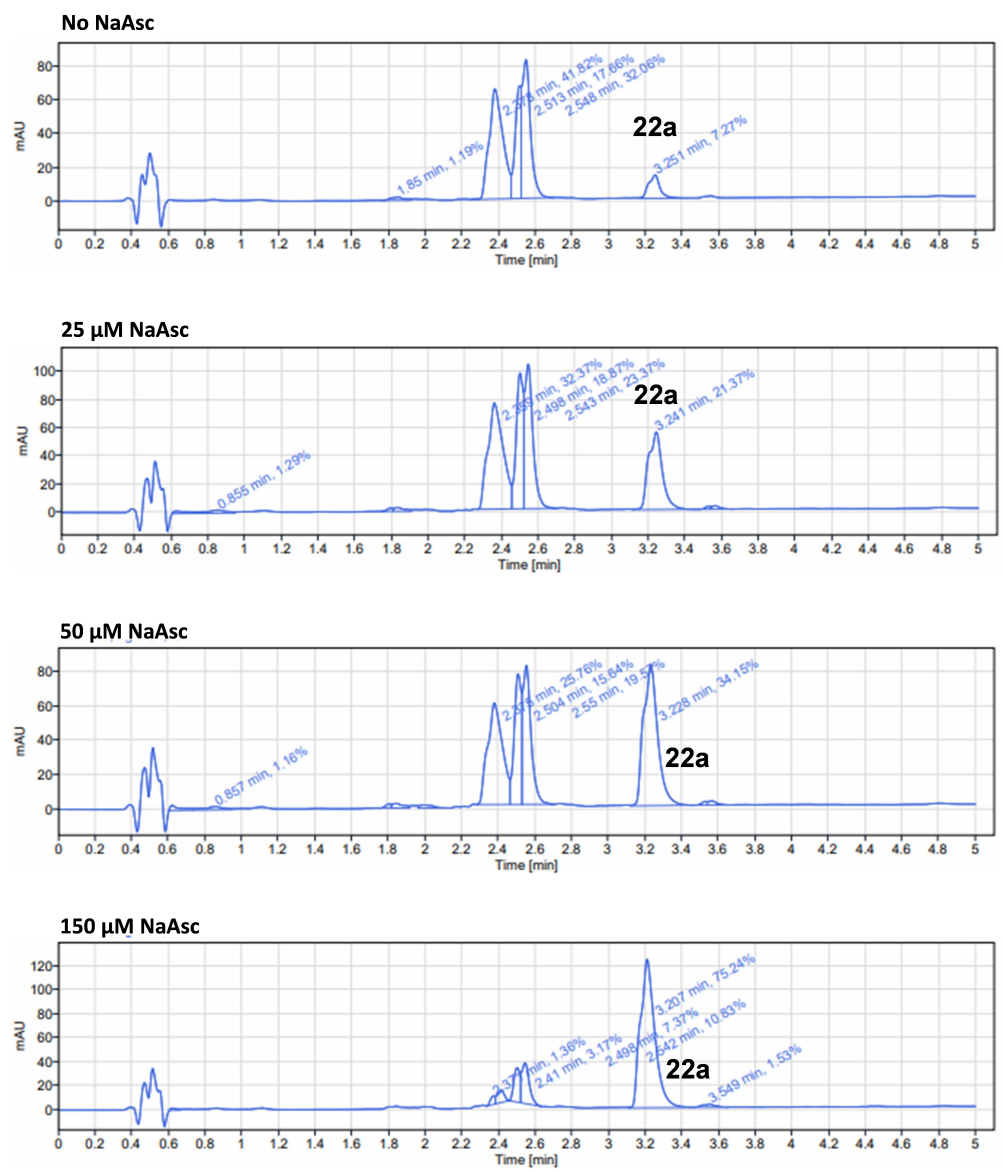
***In vitro*** **synthesis of PROTAC**. Representative HPLC chromatograms after 2 h reaction. Reaction conditions: **20 a** (100 μM), **21** (100 μM) and Cu(I)‐microdevices (1 mg/mL) were incubated with various concentrations of NaAsc in H_2_O:MeOH (70 : 30) at 37 °C in a thermomixer.

Given the superior proteolytic activity of **22 a** for ABL, we proceeded to test the *in situ* generation of this PROTAC under biologically‐relevant conditions. An *in vitro* click assay was performed in aqueous media by reacting precursors **20 a** (100 μM) and **21** (100 μM) in the presence of Cu(I)‐microdevices (1 mg/ml) at 37°C in a Thermomixer. Samples were analyzed at various time points by HPLC‐MS. As show in the chromatogram of Figure 6 (top panel), a third peak with the mass corresponding to **22 a** appears after 2 h, confirming that the strategy is able to assemble the PROTAC under these conditions. No side products were produced at detectable levels. After 24 h, the reaction had reached approx. 50 % yield (see Figure S2, Supp. Inf.). However, the reaction stalled after 24 h. To boost the CuAAC reaction, the experiment was repeated in the presence of NaAsc. According to the literature, plasma levels of NaAsc ranges between 50 and 150 μM.^[67]^ Therefore, reactions were performed using various NaAsc concentrations up to 150 μM. As shown in Figure 6, the natural reductant NaAsc had a major effect on increasing the reaction rates, with 75 % of **22 a** being generated after 2 h incubation in the presence of 150 μM of NaAsc. As in previous studies, the reactivity of the Cu(I)‐microdevices at catalyzing the CuAAC reaction was dependent on maintaining the Cu(I) oxidation state.

**Figure 7:**
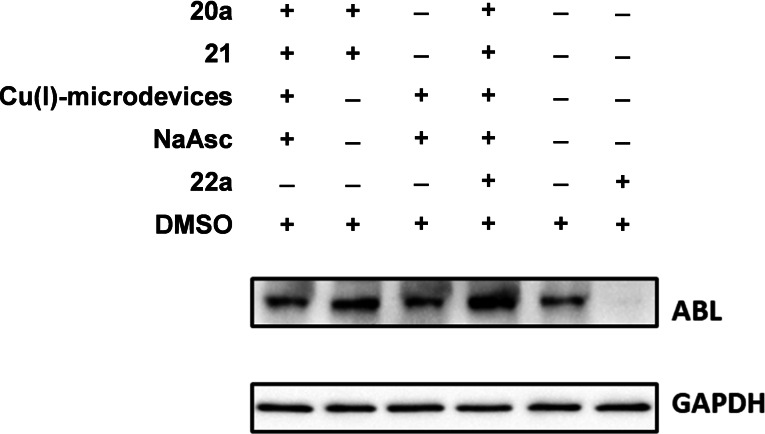
***In situ*** **PROTAC assembly in cancer cell culture**. MDA‐MB‐231 were treated with **20 a** (0.25 μM), **21** (0.25 μM), Cu(I)‐microdevices (1 mg/ml) and NaAsc (150 μM) for 48 h prior to cell lysis and analysis by Western blot. Negative controls lack one or more of the key components. **22 a** (0.25 μM) only was used as positive control.

***In situ*** **PROTAC synthesis**. With the conditions optimized for the click assay, we progressed to study whether the *in situ* assembly of PROTAC **22 a** could lead to ABL degradation in cancer cell culture. Breast cancer MDA‐MB‐231 cells were treated with the precursors **20 a** and **21**, and Cu(I)‐microdevices (1 mg/mL), in culture media supplemented with NaAsc (150 μM). After 24 h incubation, cells were lysed and immunoblotting carried out as described in the Supp. Inf. As negative controls, cells were also incubated with the PROTAC precursors in the absence of catalyst, and with the catalyst in the absence of precursors. Treatment with **22 a** was used as positive control. Disappointingly, the *in situ* PROTAC assembly experiment did not lead to a reduction of ABL protein levels, whereas **22 a** led to potent ABL degradation (Figure 7). Since the formation of **22 a** was confirmed by HPLC, given that the precursors can also enter cells freely, we hypothesized that the stronger affinity of the precursors for their targets (E3 ligase for **20 a** and ABL kinase for **21**) outcompetes the assembled PROTAC for binding sites thus preventing the formation of the tertiary complex and impeding ABL degradation. To provide evidence of this, we performed the *in situ* PROTAC assembly experiment in the presence of fully assembled **22 a**. As shown in Figure 7, in the presence of precursors **20 a** and **21**, PROTAC **22 a** was not able to reduce protein levels. In our opinion, this competition phenomenon, which is analogous to the hook effect, becomes a limitation for the application of *in situ‐*assembled PROTACs to induce site‐specific protein degradation as long as the precursors are free to interact independently with their targets. Alternative strategies capable of accelerating the PROTAC assembly[^68^, ^69^] might reduce the impact of this situation if combined with spatiotemporal control.

## Conclusions

In this manuscript we have reported an investigation on the application of solid‐supported Cu(I) to facilitate bioorthogonal uncaging and assembling reactions. To this aim, off‐on Cu(I)‐sensitive sensors and ligand‐functionalized polymeric devices were developed and tested under conditions compatible with life. Of note, from the range of novel tridentate ligands used to decorate the solid support, the isopropyl‐featuring ligand (least lipophilic of the series) mediated superior conversion rates at physiological conditions. In addition, a novel strategy was devised to modulate kinase activity in cancer cell culture to explore the reactivity scope offered by Cu(I). While CuAAC reactions were successfully mediated by Cu(I)‐microdevices under biocompatible conditions, the strategy of assembling a PROTAC *in situ* did not succeed in inducing protein degradation due to competition with the pre‐assembled units, what discourages the use of this approach to achieve spatially‐controlled protein degradation. Future studies led in this direction should explore coupling reactions with ultrafast kinetics to avoid the competition issues found with the present strategy. As a final note, it is important to highlight that Cu(I) reactions were accelerated under NaAsc levels typically found *in vivo* in the intersticial fluid.^[67]^ While this is encouraging, the reliance on reductant agents to maintain and regenerate Cu(I) suggests that the most relevant tumors where this method could find application are those that have become hypoxic due to reduced oxygen supply, which are common in advanced malignancies. Follow‐on studies should take into account the Cu(I)/Cu(II) redox potential and explore theranostic applications and anatomical sites that are favorable for Cu(I) catalysis.

## Conflict of interests

The authors declare no conflicts of interest.

1

## Supporting information

As a service to our authors and readers, this journal provides supporting information supplied by the authors. Such materials are peer reviewed and may be re‐organized for online delivery, but are not copy‐edited or typeset. Technical support issues arising from supporting information (other than missing files) should be addressed to the authors.

Supporting Information

## Data Availability

The data that support the findings of this study are available in the supplementary material of this article.
