# Supplementary material for: Development of Biocompatible Cu(I)‐Microdevices for Bioorthogonal Uncaging and Click Reactions
Source: Chemistry. 2024 Apr 16;30(30):e202400611. doi: 10.1002/chem.202400611 (PMC11497292; doi:10.1002/chem.202400611)
Supplement: Supplementary file 1 — Supporting Information [file CHEM-30-e202400611-s001.pdf]

// Chemistry–A European Journal

Supporting Information

Development of Biocompatible Cu(I)-Microdevices for Bioorthogonal Uncaging and Click Reactions

Melissa van de L'Isle, Stephen Croke, Teresa Valero, and Asier Unciti-Broceta*

Supporting Information

Development of Biocompatible Cu(I)-Microdevices for Bioorthogonal PROTAC Assembly

Melissa van de L'Isle,[a,†] Stephen Croke,[a,†] Teresa Valero,[a,b] and Asier Unciti-Broceta*[,a]

[a] *Edinburgh Cancer Research, Institute of Genetics & Cancer, University of Edinburgh, Crewe Road South, Edinburgh EH4 2XR, UK*

[b] *Department of Medicinal & Organic Chemistry and Excellence Research Unit of Chemistry applied to Biomedicine and the Environment, Faculty of Pharmacy, University of Granada, Campus de Cartuja s/n, 18071, Granada, Spain. GENYO, Centre for Genomics and Oncological Research, Pfizer/University of Granada/Andalusian Regional Government, Avda. Ilustración 114,18016, Granada, Spain. Instituto de Investigación Biosanitaria ibs.GRANADA, Granada, Spain.*

Table of Contents:

1. General experimental protocols
2. Cu(I)-sensors for fluorimetric assays
3. Synthesis of the tris-triazole ligands
4. Copper loading onto ligand-functionalized resins
    a. Table S1
    b. Figure S1
5. Cu(I)-assemblable PROTACs
6. Biological assays
    a. Figure S2
    b. Figure S3
7. Literature references

1. General experimental protocols

Chemicals

Chemicals were purchased from Sigma-Aldrich UK, Fluorochem UK, Acros Organics, TCI UK, Alfa Aesar UK or Fisher Scientific UK and used without further purification. Compound **20b** ([9]01833) and compound **20c** (901834) were purchased from Sigma-Aldrich UK.

Chromatography

TLC silica gel plates Si 60 F_{254} from Merck were used for TLC analysis. Column Chromatography was done with commercially available silica gel (220 – 440 mesh, Sigma-Aldrich).

Analytic UPLC-MS data was obtained by using Agilent 1260 infinity II system equipped with an InfinityLab Poroshell 120 EC-C18, 3.0 x 100 mm, 2.7 µm, 1000 bar (PN 695575-302) with a binary mixture of A (0.1% formic acid, 99.9% H2O) and B (0.1% formic acid, 99.9% MeOH) as a mobile phase (flow = 1.0 mL/min) in a starting linear gradient as described.

Nuclear Magnetic Resonance Spectroscopy

Nuclear magnetic resonance (NMR) spectra were recorded on a Bruker AV-400 (400 Hz) instrument, in the deuterated solvent stated. ^{1}H NMR chemical shifts are quoted to the nearest 0.01 ppm and referenced to the residual non-deuterated solvent peak. Coupling constants (J) are given to the nearest 0.1 Hz. The following abbreviations are used to indicate signal multiplicity: s singlet, d doublet, t triplet and m multiplet. ^{13}C NMR chemical shifts are quoted to the nearest 0.01 ppm and referenced to the deuterated solvent peak.

2. Cu(I)-sensors for fluorimetric assays

Synthesis of turn-off/on Resorufin derivatives

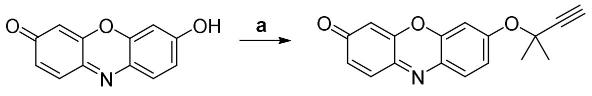

Res (1) 2

a) Synthesis of Resorufin-dimethylpropargyl (2)

Resorufin **1** (1.0 eq., 200.0 mg, 0.938 mmol) was dissolved with DBU (1.1 eq., 93 µL, 0.62 mmol) in anhydrous MeCN (4.0 mL) under nitrogen atmosphere, in a flask that was preliminary dried with a heatgun under high vacuum and 3-times flushed with nitrogen (g). Next, 3-chloro-3-methylbut-1-yne (2.0 eq., 193 µL, 1.72 mmol) and copper chloride (0.1 eq., 15 mg, 0.086 mmol) were carefully added. The reaction mixture was allowed to stir at room temperature and the product formation was monitored by HPLC-MS. After 4 hours the mixture was concentrated under reduced pressure and directly purified via semipreparative-TLC, with 2.5% ethyl acetate in acetonitrile. The product was found as orange band at Rf = 0.65. Collection of the quenched fluorophore proceeded by eluting the silica fraction containing the product in EtOAc, followed by gravity filtration and removal of the solvents *in vacuo* to give Reso-O-dmP **2** as orange solid (40.1 mg, 0.144 mmol, 15%)

^{1}H NMR (500 MHz, DMSO): δ 7.81 (d, *J* = 8.9 Hz, 1H), 7.55 (d, *J* = 9.8 Hz, 1H), 7.31 (d, *J* = 2.6 Hz, 1H), 7.21 (dd, *J* = 8.8, 2.6 Hz, 1H), 6.81 (dd, *J* = 9.8, 2.1 Hz, 1H), 6.31 (d, *J* = 2.1 Hz, 1H), 3.91 (s, 1H), 1.71 (s, 6H). **^{13}C NMR (126 MHz, DMSO):** δ 185.41, 159.18, 149.64, 146.01, 144.38, 134.95, 133.96, 130.92, 128.49, 117.48, 105.80, 105.24, 84.53, 78.39, 73.19, 29.12, 28.96. **HRMS (ESI) (m/z):** $[M+H]^+$ calcd. for $C_{17}H_{14}NO_3$, 280.09682 ; found $[M+H]^+$: 280.0964

^{1}H NMR spectrum of compound 2. Spectra were taken in DMSO-d$_6$ at 500 MHz.

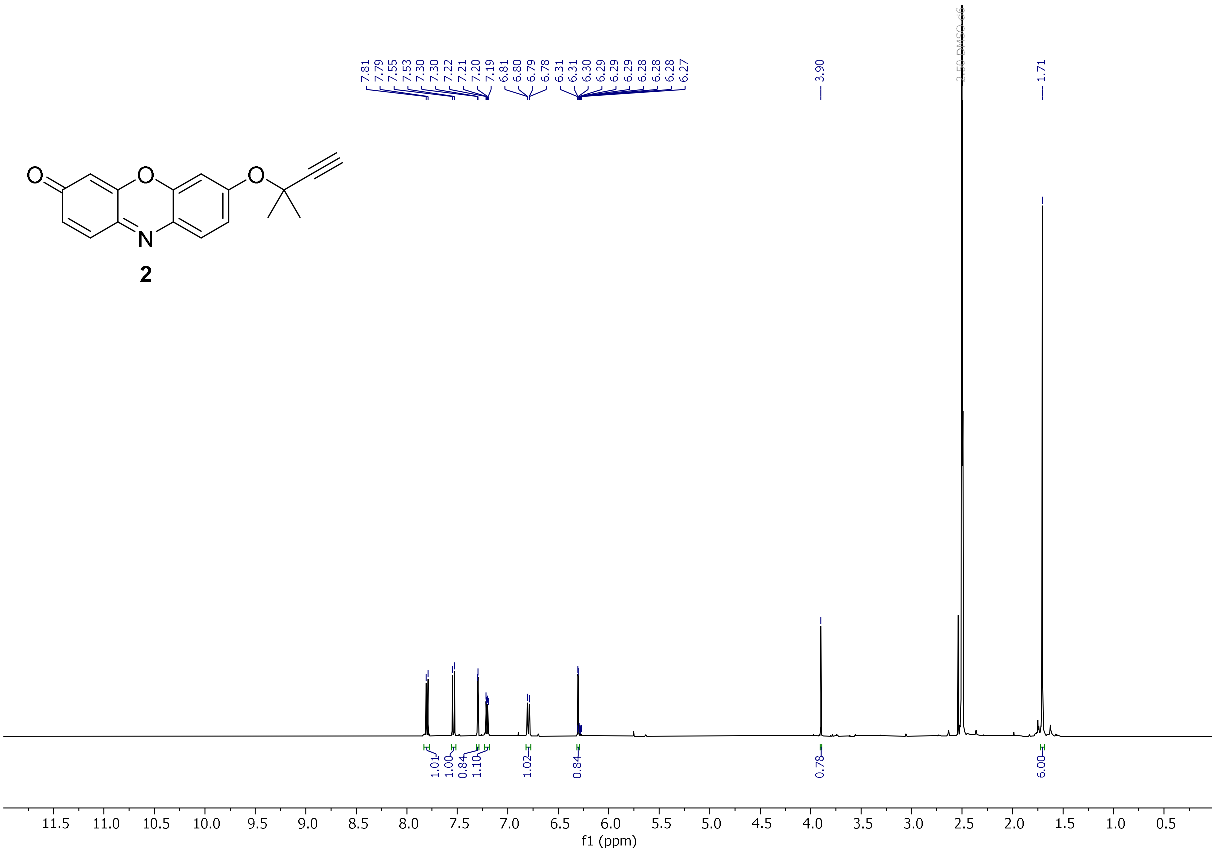

^{13}C NMR spectrum of compound 2. Spectra were taken in DMSO-d$_6$ at 126 MHz.

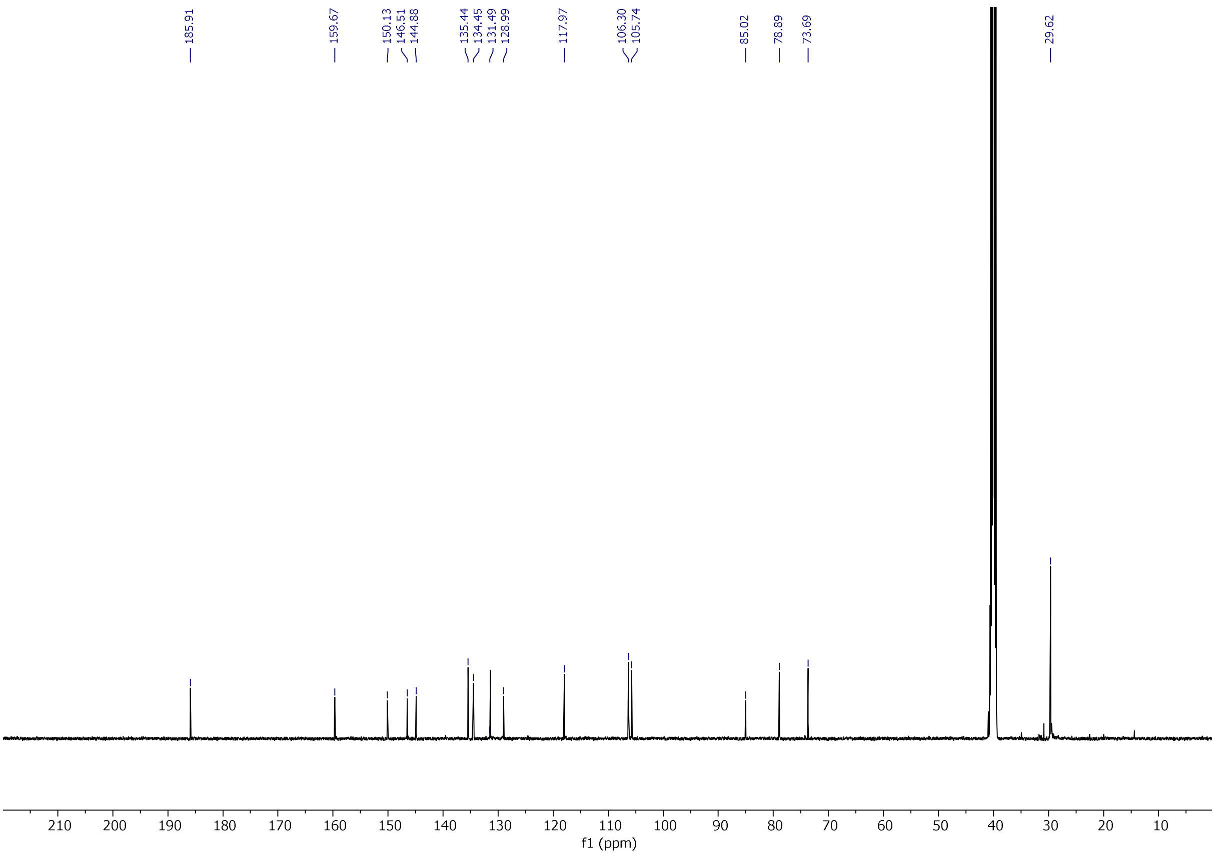

Synthesis of NBD-NHMe-dmPoc (5a) and NBD-NHMe-Poc (5b)

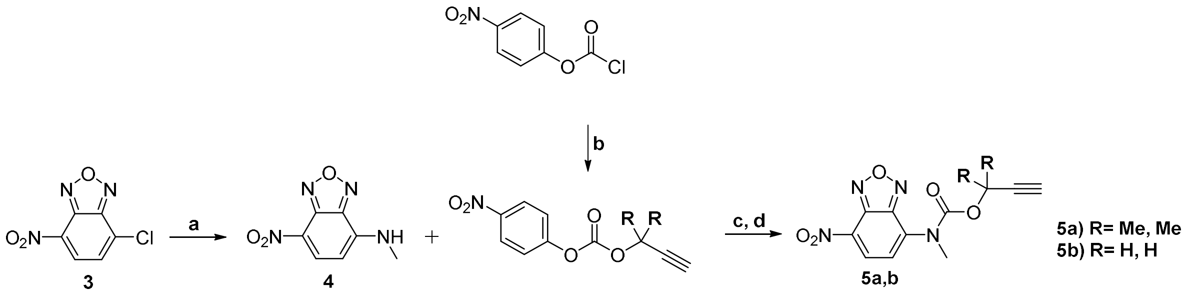

a) Synthesis of NBD-methylamine (4)

The NBD-methylamine **4** was prepared in similar fashion as reported by Ghosh et al.[1] The NBD-chloride **3** (1.0 eq., 150.0 mg, 0.75 mmol) was dissolved in ethanol (4.0 mL) with methylamine (1.2 eq., 0.45 mL, 0.90 mmol) added to the solution. After 30 minutes of stirring at room temperature, the starting material was fully converted into desired product **4**. The reaction mixture was diluted with brine and extracted with EtOAc. After concentration *in vacuo*, the crude was purified by flash chromatography. The NBD-methylamine **4** was obtained as green solid (120 mg, 0.788 mmol, 82%)

^{1}H NMR (500 MHz, DMSO): δ 9.48 (s, 1H), 8.54 (d, J = 9.0 Hz, 1H), 6.32 (d, J = 9.0 Hz, 1H), 3.07 (s, 3H)

b) Synthesis of 4-nitrophenol-dimethylpropargyloxycarbonate

The 4-nitrophenol-dm-Poc reagent was prepared by addition of 4-nitrophenylchloroformate (1.0 eq., 500.0 mg, 2.48 mmol) to a solution of 2-methyl-3-butyn-2-ol (1.5 eq., 361 µL, 3.72 mmol) and pyridine (1.5 eq., 300 µL, 3.72 mmol) in DCM (17.0 mL), according to the procedure by Bertrand and Gesson.[2] The isolation was achieved via semipreparative-TLC, with 10% EtOAC in *n*-hexane as eluent. The product was found with UV detection at Rf=0.50. Subsequently, the product fraction on silica was extracted and filtered off with EtOAC. The solvents were removed under reduced pressure, 4-nitrophenol-dm-Poc was obtained as white solid (325.7 mg, 1.31 mmol, 53%)

^{1}H NMR (500 MHz, CDCl3): δ 8.27 (d, J = 9.1 Hz, 2H), 7.41 (d, J = 9.3 Hz, 2H), 2.64 (s, 1H), 1.81 (s, 6H)

c) Synthesis of NBD-methylamine-dimethylpropargyloxycarbamate (5a)

The NBD-NHMe **4** (1.0 eq., 20.0 mg, 0.103 mmol) was dissolved in anhydrous DMF (1.5 mL), under nitrogen atmosphere, together with triethylamine (4.0 eq., 58.0 µL, 0.412 mmol). Subsequently, the 4-nitrophenol-dmPoc **7a** (1.2 eq., 31.0 mg, 0.124 mmol) was added and allowed to stir at room temperature overnight. Then, the reaction mixture was concentrated and the crude oil was purified via semipreparative-TLC, with 35% ethyl acetate in *n*-hexane as eluent. The product was found by UV detection at R*f*=0.30 and collected by filtrated with EtOAc as eluent. The solvents were removed *in vacuo* and NBD-NHMe-dmPoc **5a** was obtained as yellow solid (14.87 mg, 0.049 mmol, 47%)

^{1}H NMR (500 MHz, DMSO): δ 8.73 (d, *J* = 8.0 Hz, 1H), 7.69 (d, *J* = 8.2 Hz, 1H), 3.58 (s, 1H), 3.47 (s, 3H), 1.58 (s, 6H). **^{13}C NMR (126 MHz, DMSO):** δ 189.20, 184.94, 181.21, 176.02, 170.93, 170.57, 161.43, 122.04, 113.22, 111.13, 75.01, 66.02. **HRMS (ESI) (m/z):** $[M+Na]^+$ calcd. for $C_{13}H_{12}N_4O_5$, 327,06999; found $[M+Na]^+$: 327.0694

d) Synthesis of NBD-methylamine-propargyloxycarbamate (5b)

Synthesis and purification proceeded in similar fashion as the reported for the reported NBD-NHEt-Poc analogue.[3] The NBD-NHMe-Poc **5b** was obtained as yellow solid (9.5 mg, 0.034 mmol, 33%)

^{1}H NMR (500 MHz, CDCl3): δ 8.52 (d, *J* = 8.0 Hz, 1H), 7.60 (d, *J* = 8.2 Hz, 1H), 4.84 (d, *J* = 2.4 Hz, 2H), 2.53 (t, *J* = 2.4 Hz, 1H). **^{13}C NMR (126 MHz, $CDCl_3$):** δ 153.73, 147.27, 143.79, 138.51, 131.48, 123.30, 77.36, 77.08, 75.98, 54.67, 38.06.

HRMS (ESI) (m/z): $[M+Na]^+$ calcd. for $C_{11}H_8N_4O_5$, 299.03869; found $[M+Na]^+$: 299.0395

^{1}H NMR spectrum of compound 5a. Spectra were taken in DMSO-d6 at 500 MHz.

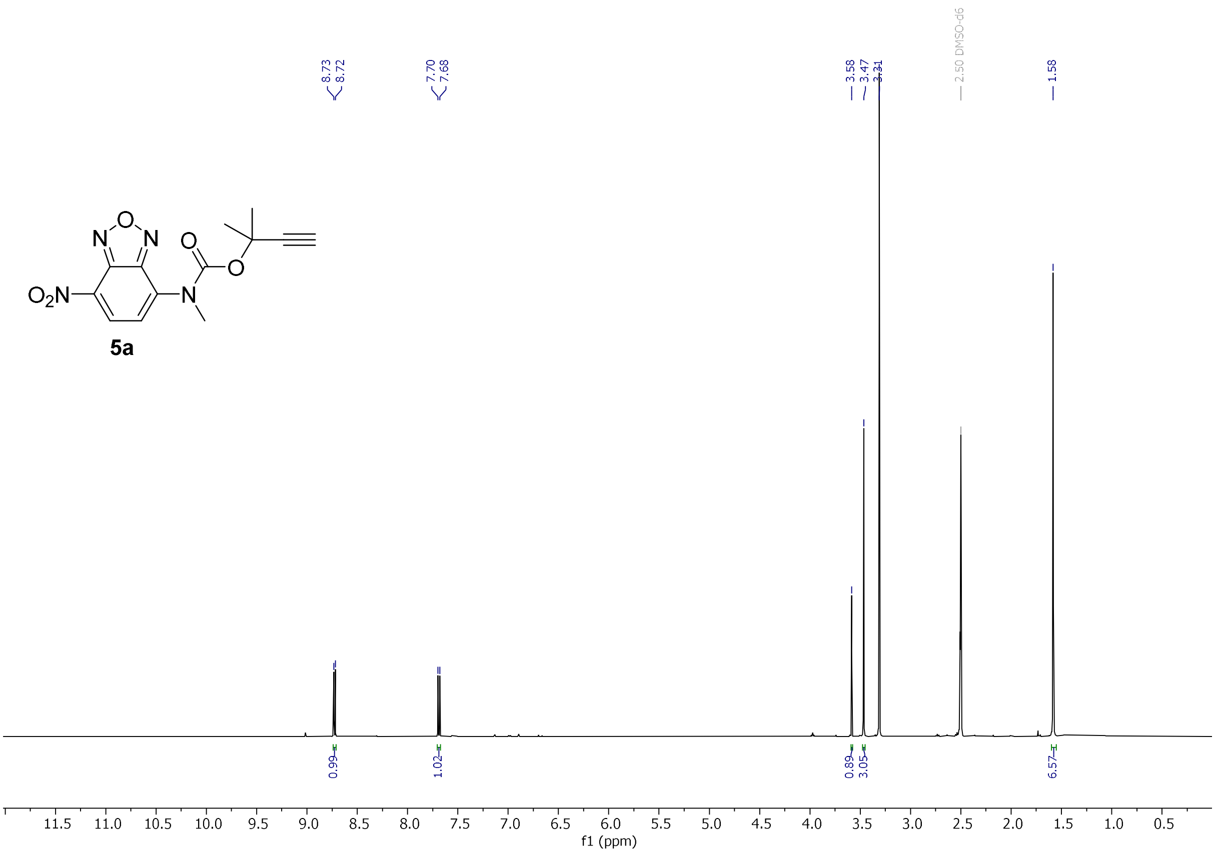

^{13}C NMR spectrum of compound 5a. Spectra were taken in DMSO-d6 at 126 MHz.

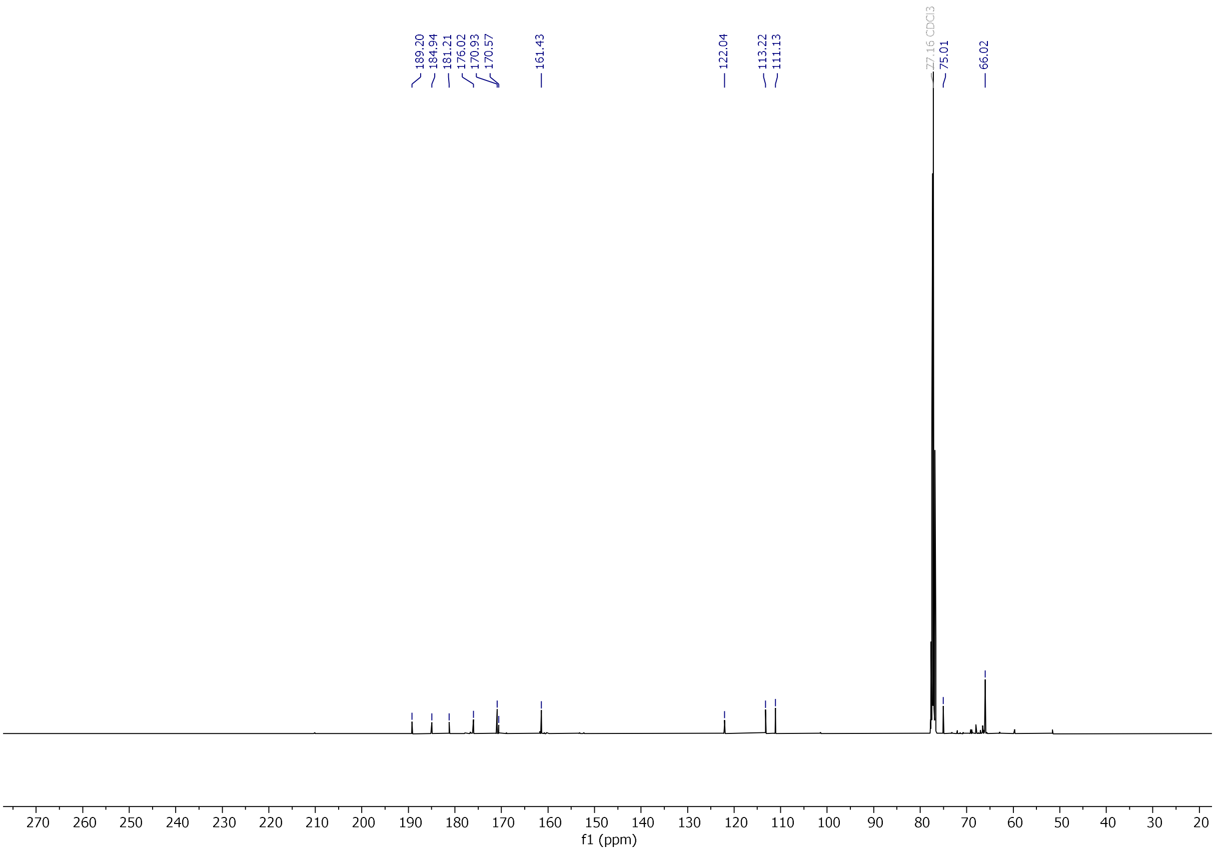

^{1}H NMR spectrum of compound 5b. Spectra were taken in $CDCl_3$-d_1 at 500 MHz.

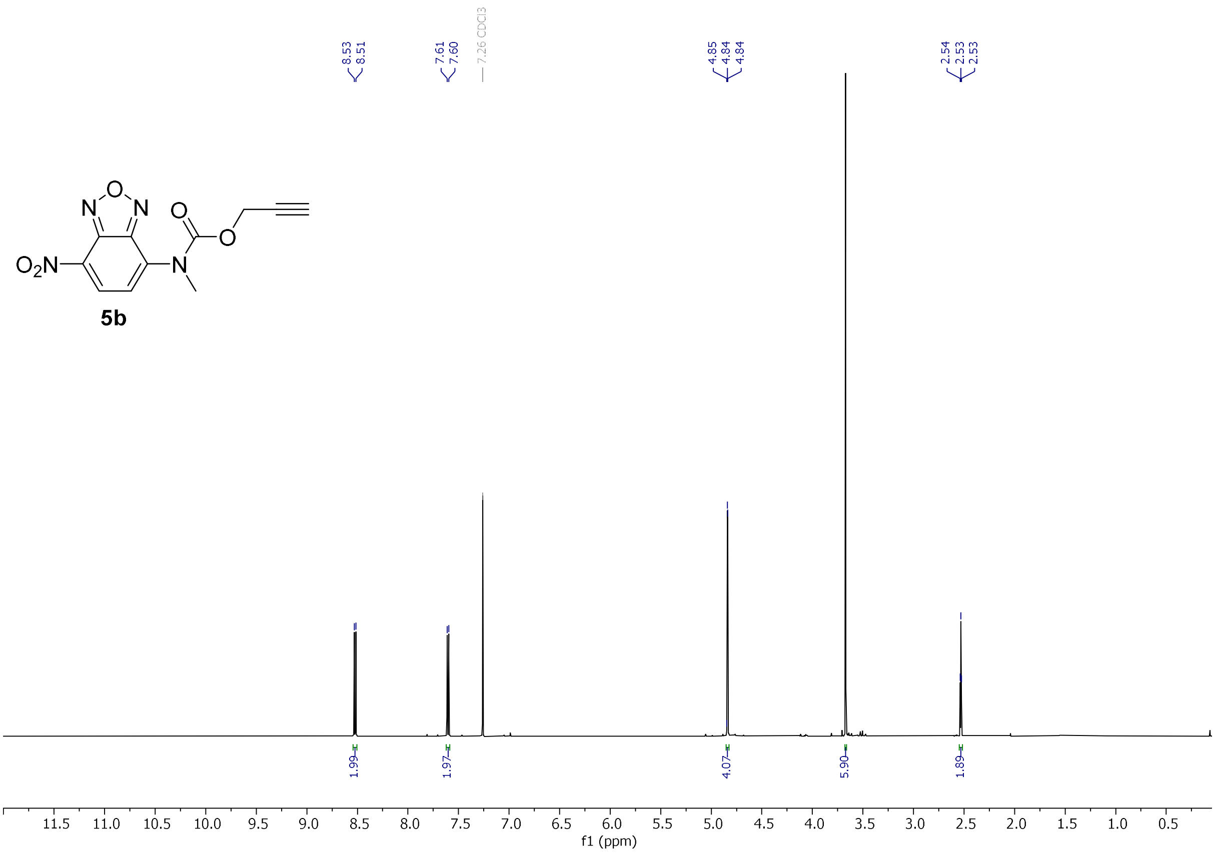

^{13}C NMR spectrum of compound 5b. Spectra were taken in $CDCl_3$-d_1 at 126 MHz.

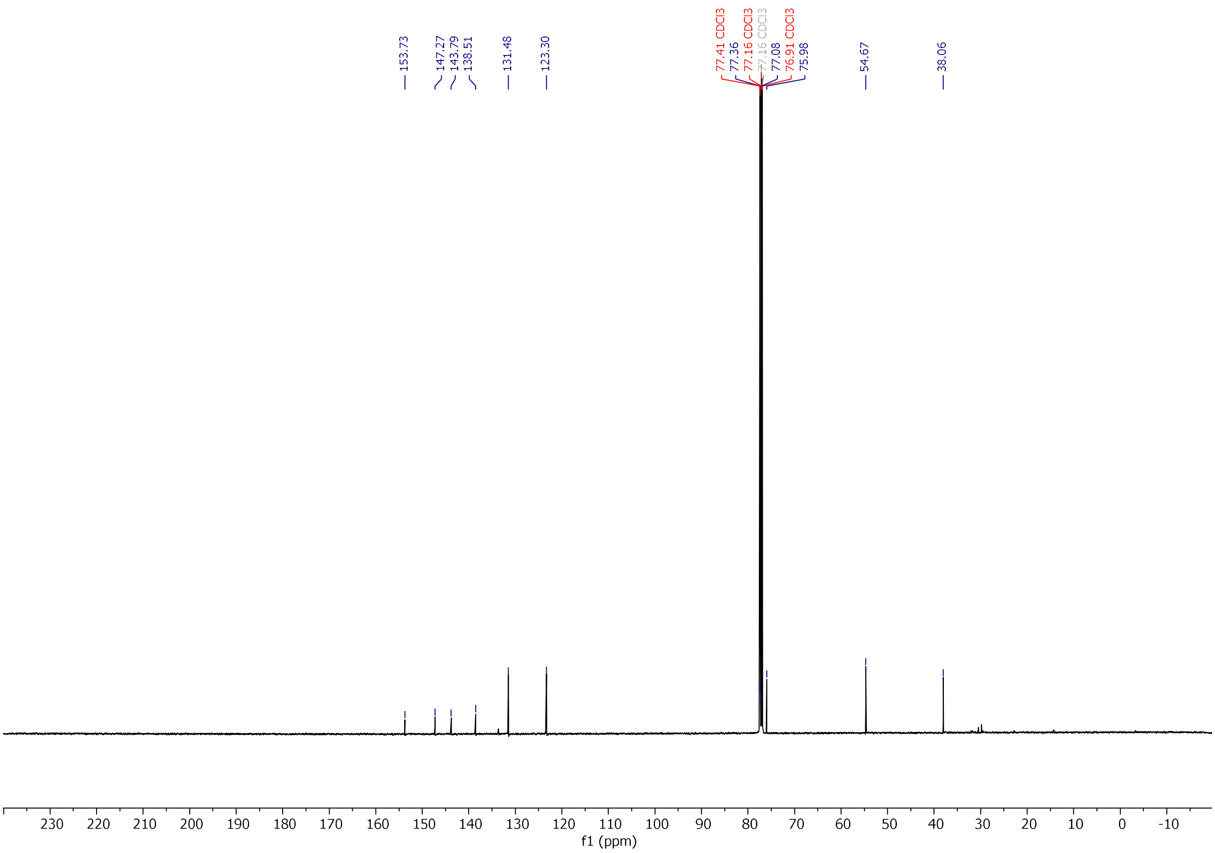

3. Synthesis of the tris-triazole ligands

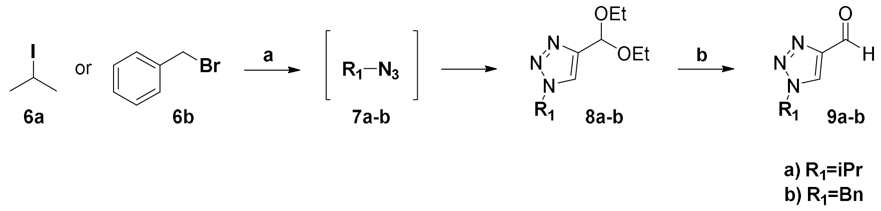

a) Acetal synthesis via the in situ generation of alkyl-azides (8a-b)

In order to achieve the in situ generation of the functionalised azides, sodium azide (3.0 eq., 2.34 g, 36.0 mmol) and sodium bicarbonate (3.0 eq., 3.0 g, 36.0 mmol) are dissolved in 1:1 mixture of *tert*-BuOH/H_2O (50 ml). To this mixture, the isopropyl iodide or benzyl bromide (3.0 eq., 36.0 mmol) were added and stirred at room temperature for 5-10minutes. Next, the 3,3-diethoxy-but-1-yne (1.0 eq., 1.73 mL, 12.0 mmol), copper(II) sulfate pentahydrate (0.06 eq., 184 mg, 0.74 mmol) and sodium ascorbate (0.4 eq., 960 mg, 4.85 mmol). The reaction mixtures was allowed to stir at 70 degrees Celsius for 24 hours. After allowing the reaction mixture to cool-down to room temperature, an extraction was performed by the addition of 1:1 brine/sodium bicarbonate sat. aq. (300 ml) and ethyl acetate (3x200 ml). The organic phases were collected, dried over $MgSO_4$, filtrated and concentrated under reduced pressure. The crude yellow oil obtained, required to be dried in the high vacuum oven at 40 degrees Celsius for at least 24 hours. The beige product **8a-b** solid was directly used in the next synthetic step, to prevent product loss from flash chromatography purification.

* For analytical purposes the crude oil containing **8a** was purified by flash column chromatography, with 10 to 60% EtOAc in *n*-hexane, product **8a** was obtained as clear oil (1.45 g, 6.80 mmol, 57%).

^{1}H NMR (500 MHz, $CDCl_3$): δ 7.58 (s, 1H), 5.70 (s, 1H), 4.87 – 4.77 (m, 1H), 3.65 (ddq, J = 43.7, 9.5, 7.1 Hz, 4H), 1.57 (dd, J = 6.8, 0.9 Hz, 6H), 1.26 – 1.21 (m, 6H).

b) Aldehyde synthesis by trifluoroacetic acid oxidation of acetals (9a-b)

The acetal **8a-b** (1.0 eq., 1.45 g, 6.80 mmol) was added to a 3:1 mixture of dichloroethane/distilled H_2O (10.5 mL) at room temperature. Trifluoroacetic acid (1.0 mL) was slowly added to the solution and stirred at room temperature until the acetal was completely consumed, TLC monitoring with $KMnO_4$ staining analysis as **8a** and **9a** are not UV detectable. Then sodium bicarbonate sat. aq. (150 mL) and brine (150 mL) were added, extracted with DCM (3 x 250 mL), the organic phase were combined, dried over

$MgSO_4$, filtrated and concentrated by rotary evaporation. The liquid product was several times co-evaporated with toluene and either high vacuum dried overnight or directly used in the next synthetic step. In general the yields were determined over 2-steps.

1-isopropyl-1H-1,2,3-triazole-4-carbaldehyde 9a

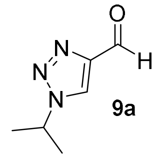

9a

Data in agreement as reported by L'abbé et al.[4]

White solid (527.2 mg, 3.79 mmol, 86% over 2-steps)

^{1}H NMR (500 MHz, $CDCl_3$): δ 10.13 (s, 1H), 8.12 (s, 1H), 4.89 (p, *J* = 6.7 Hz, 1H), 1.62 (d, *J* = 6.8 Hz, 6H). **^{13}C NMR (126 MHz, $CDCl_3$):** δ 185.40, 147.71, 122.95, 53.82, 23.01.

1-benzyl-1H-1,2,3-triazole-4-carbaldehyde (9b)

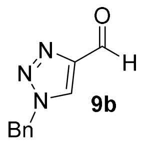

9b

Data in agreement as reported by Chan and Fokin.[5]

White solid (2.06 g, 11.0 mmol, 92% over 2-steps)

^{1}H NMR (500 MHz, $CDCl_3$): δ 10.13 (s, 1H), 7.98 (s, 1H), 7.41 (dd, *J* = 5.0, 1.9 Hz, 3H), 7.35 – 7.27 (m, 2H), 5.59 (s, 2H). **^{13}C NMR (126 MHz, $CDCl_3$):** δ 185.21, 148.22, 133.48, 129.58, 128.55, 125.17, 54.77.

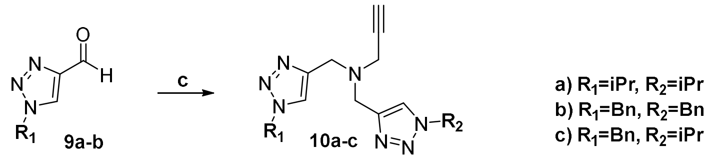

c) di-triazole propargyl amines via reductive aminations (10a-c)

In a round bottom flask containing the aldehyde **9a-c** (2.2 eq., 1.841 g, 13.2 mmol) and propargyl amine (1.0 eq., 381 μL, 6.0 mmol) dissolved in DCM (45 mL), was sodium triacetoxyborohydride (3.0 eq., 5.5 g, 26.04 mmol) portion-wise added. The reaction mixture was stirred at room temperature for 2 hours when complete conversion was detected by HPLC-MS. In order to quench the sodium triacetoxyborohydride, distilled H_2O (40 mL) and conc. sulfuric acid (2 mL) were added, to the ice-bath cooled reaction mixture at 0 degrees Celsius. After 15 minutes, while stirring continued, potassium carbonate and H_2O were carefully added until pH=7 was reached.[5] Then extraction was performed with

addition of H_2O (100 mL) and DCM (3x200 mL), the organic layers were collected, dried over $MgSO_4$, filtrated and concentrated by rotational evaporation. The crude product was purified by flash column chromatography with 5% Et_3N in EtOAc and the product were concentrated *in vacuo*.

***N*,*N*-Bis[(1-isopropyl-1H-1,2,3-triazol-4-yl)methyl]prop-2-yn-1-amine (10a)**

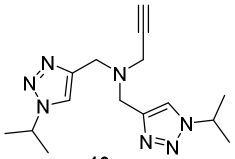

10a

White solid (1.261 g, 4.18 mmol, 70%)

^{1}H NMR (500 MHz, $CDCl_3$): δ 7.61 (s, 2H), 4.80 (hept, *J* = 6.8 Hz, 2H), 3.86 (s, 4H), 3.38 (d, *J* = 2.4 Hz, 2H), 2.28 (t, *J* = 2.4 Hz, 1H), 1.56 (d, *J* = 6.8 Hz, 13H). **^{13}C NMR (126 MHz, $CDCl_3$):** δ 144.01, 120.72, 78.64, 73.81, 53.01, 47.98, 42.30, 23.13. **HRMS (ESI) (m/z):** $[M+H]^+$ calcd. for $C_{15}H_{24}N_7$, 302.20877; found $[M+H]^+$: 302.20877 and $[M+Na]^+$: 324.1906

***N*,*N*-Bis[(1-benzyl-1H-1,2,3-triazol-4-yl)methyl]prop-2-yn-1-amine (10b)**

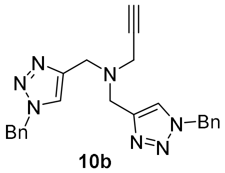

10b

White solid (527.5 mg, 1.32 mmol, 31%)

^{1}H NMR (400 MHz, $CDCl_3$): δ 8.02 (s, 2H), 7.36 (m, 5H), 7.29 – 7.26 (m, 3H), 5.53 (s, 4H), 4.22 (s, 4H), 3.63 (s, 2H), 2.54 (s, 1H). Data in agreement with the reported NMR spectra.[5]

***N*-1-Benzyl-1H-1,2,3-triazole-4-methylpropargyl amine**

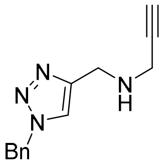

Beige solid (458.9 mg, 2.03 mmol, 47%)

HPLC-MS (ESI): 2 µL injection, gradient 5 to 95% MeCN in H_2O, 5 minute run; **product rt:** 1.613 min, **(m/z):** $[M]^+$ calcd. for $C_{13}H_{14}N_4$, 226.12, found $[M]^+$ 226.8 $[M+Na]^+$ 249.9; **product rt:** 1.878 min, **(m/z):** found $[M]^+$ 226.9 $[M+H]^+$ 227.9 $[M+Na]^+$ 248.9

***N,N*-[(1-benzyl-1H-1,2,3-triazol-4-yl)methyl]-[(1-isopropyl-1H-1,2,3-triazol-4-yl)methyl]prop-2-yn-1-amine (10c)**

Aldehyde **10a** (1.3 eq., 140 mg, 1.3 mmol), N-1-Benzyl-1H-1,2,3-triazole-4-methylpropargyl amine (1.3 eq., 140 mg, 1.3 mmol) in DCM (5.0 mL) with sodium triacetoxyborohydride (1.5 eq., 318 mg, 1.5 mmol) 3.5 hours. Similar purification methods as to obtain **10a-b**

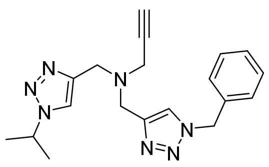

10c

White solid (82.5mg, 0.236 mmol, 24%)

^{1}H NMR (500 MHz, $CDCl_3$): δ 7.56 (s, 1H), 7.51 (s, 1H), 7.41 – 7.30 (m, 2H), 5.51 (s, 2H), 4.79 (p, *J* = 6.8 Hz, 1H), 3.84 (d, *J* = 5.4 Hz, 4H), 3.36 (d, *J* = 2.5 Hz, 2H), 2.25 (t, *J* = 2.4 Hz, 1H), 1.56 (d, *J* = 6.8 Hz, 6H). **^{13}C NMR (126 MHz, $CDCl_3$):** δ 145.01, 144.24, 134.85, 129.25, 128.85, 128.20, 123.16, 120.56, 78.73, 73.73, 54.28, 53.02, 48.05, 47.98, 42.33, 23.18. **HRMS (ESI) (m/z):** M+H]$^+$ calcd. for $C_{19}H_{24}N_7$, 350.20877; found [M+H]$^+$: 350.2092 and [M+Na]$^+$: 372.1905

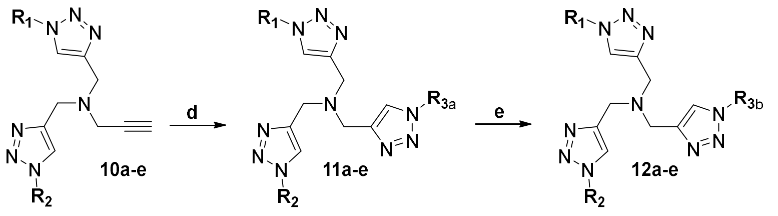

d) Tris-triazole ester formation by CuAAC (11a-e)

The synthetic strategy is a modified version by Chan et al., whereas other tris-triazole derivatives were prepared.[6] The third triazole is introduced by dissolving di-(isopropyl-triazole)-ethyl-amine propargyl **10a** or **10b-c** (1.0 eq., 250 mg, 0.829 mmol) and the corresponding ethyl azidoacetate or functionalised azides (1.2 eq.) in acetonitrile (4 mL). Copper acetate (0.1 eq., 15 mg, 0.083 mmol) and sodium ascorbate (0.4 eq., 66.0 mg, 0.332 mmol) were added and stirred at room temperature overnight. The extraction was performed by adding 1:1 brine/sodium bicarbonate sat. aq. (200 mL) and EtOAc (3x100 mL) to the reaction mixture. The organic layers were combined, dried over $MgSO_4$ and the solvents were removed *in vacuo*. In order to achieve high purity and prevent product loss by flash column chromatography, the crude oil was purified via semipreparative-TLC, with 5% triethyl amine in EtOAc as the eluent. In general, the products **6a-e** were found by UV-detection at Rf=0.45. The silica containing product was extracted by EtOAc as eluent, filtrated and concentration under reduced pressure.

* trans-esterification products and hydrolysed product were found as well

e) Saponification of tris-triazole esters into tris-triazole carboxylic acids (12a-f)

The ester **11a** (1.0 eq., 130 mg, 0.302 mmol) or **11b-e** was dispensed into MeOH (4.0 mL) with potassium hydroxide (0.310 g, 5.5 mmol). The reaction was stirred at room temperature overnight and conversion of the ester into carboxylic acid was monitored by HPLC-MS. The reaction mixture was diluted with distilled H_2O (5 mL) and drop-wise acidified with concentrated HCl until the indicator paper showed pH=1. The visible appearance of white product suspension was extracted with DCM (3x50 mL). After combining, the organic layers were dried over $NaSO_4$, concentrated by rotary evaporation and dried under high vacuum, to obtain the product **12a-e** as white solid.

2-(4-((bis((1-isopropyl-1H-1,2,3,triazol-4-yl)methyl)amino)methyl)-1H-1,2,3,-triazol-1-yl)ethyl acetate (11a)

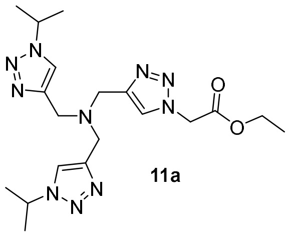

11a

White solid (134.2 mg, 0.312 mmol, 38%)

^{1}H NMR (500 MHz, CDCl$_3$): δ 8.04 (s, 1H), 7.95 – 7.77 (s, 2H), 5.11 (s, 2H), 4.82 – 4.70 (m, *J* = 6.8 Hz, 2H), 4.20 (q, *J* = 7.1 Hz, 2H), 3.87 (s, 6H), 1.54 (d, *J* = 6.8 Hz, 12H), 1.24 (t, *J* = 7.1 Hz, 3H). **^{13}C NMR (126 MHz, CDCl$_3$):** δ 166.26, 62.63, 60.53, 53.34, 51.12, 46.96, 29.84, 23.14, 21.19, 14.22. **HRMS (ESI) (m/z):** $[M+H]^+$ calcd. for $C_{19}H_{30}N_{10}O_2$, 431.26; found $[M+H]^+$: 431.2636 and $[M+Na]^+$: 453.2458

2-(4-((bis((1-isopropyl-1H-1,2,3,triazol-4-yl)methyl)amino)methyl)-1H-1,2,3,-triazol-1-yl)methyl carboxylic acid (12a)

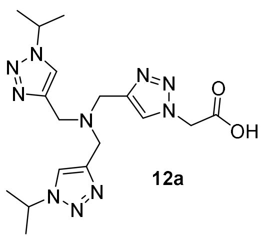

12a

White solid (100.7 mg, 0.250 mmol, 83%)

^{1}H NMR (500 MHz, CDCl$_3$): δ 8.57 (s, 1H), 8.40 (s, 2H), 5.24 (s, 2H), 4.84 (hept, *J* = 6.9 Hz, 2H), 4.40 (d, *J* = 7.6 Hz, 6H), 1.61 (d, *J* = 6.8 Hz, 14H). **^{13}C NMR (126 MHz, CDCl$_3$):** δ 168.17, 136.23, 135.52, 129.66, 125.96, 53.94, 51.31, 46.54, 46.32, 23.03. **HRMS (ESI) (m/z):** $[M+H]^+$ calcd. for $C_{17}H_{27}N_{10}O_2$, 403.23130; found $[M+H]^+$: 403.2322 and $[M+Na]^+$: 425.2129

2-(4-((bis((1-benzyl-1H-1,2,3,triazol-4-yl)methyl)amino)methyl)-1H-1,2,3,-triazol-1-yl)ethyl acetate (11b)

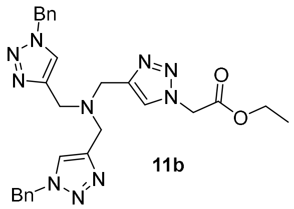

White solid (86.2 g, 0.164 mmol, 50%)

^{1}H NMR (500 MHz, $CDCl_3$): δ 8.03 (s, 1H), 7.79 (d, *J* = 22.5 Hz, 2H), 7.35 – 7.24 (m, 6H), 7.23 – 7.18 (m, 4H), 5.46 (s, 4H), 5.08 (s, 2H), 4.19 (q, *J* = 7.2 Hz, 2H), 3.86 (s, 6H), 1.22 (t, *J* = 7.2 Hz, 3H). **^{13}C NMR (126 MHz, $CDCl_3$):** δ 166.22, 134.64, 129.30, 128.94, 128.28, 128.21, 62.64, 54.44, 51.08, 46.91, 14.22. **HRMS (ESI) (m/z):** $[M+H]^+$ calcd. for $C_{27}H_{31}N_{10}O_2$, 527.26260; found $[M]^+$: 527.2632, $[M+H]^+$: 527.2632 and $[M+Na]^+$: 549.2443

2-(4-((bis((1-benzyl-1H-1,2,3,triazol-4-yl)methyl)amino)methyl)-1H-1,2,3,-triazol-1-yl)methyl carboxylic acid (12b)

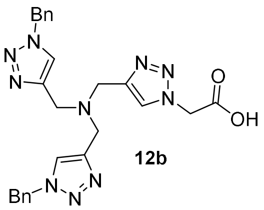

White solid (53.8 mg, 0.108 mmol, 43% over 2-steps)

^{1}H NMR (500 MHz, $CDCl_3$): δ 10.12 (s, 1H), 8.44 (s, 1H), 8.30 (s, 2H), 7.39 – 7.23 (m, 10H), 5.52 (s, 4H), 5.13 (s, 2H), 4.40 – 4.32 (m, 6H). **^{13}C NMR (126 MHz, $CDCl_3$):s** δ 174.36, 168.42, 136.27, 134.30, 129.57, 129.31, 129.03, 128.30, 128.07, 56.25, 54.59, 51.35, 46.71, 46.22. **HRMS (ESI) (m/z):** $[M+H]^+$ calcd. for $C_{25}H_{27}N_{10}O_2$, 499.23130; found $[M+H]^+$: 499.2322

4-(((1-benzyl-1H-1,2,3,triazol-4-yl)methyl)amino)-1-isopropyl-1H-1,2,3,triazol-4-yl)methyl)-1H-1,2,3,-triazol-1-yl)ethyl acetate (11c)

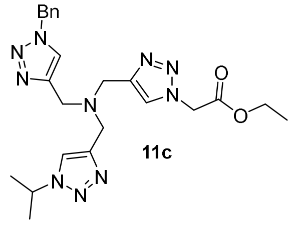

White solid (82.4 mg, 0.172 mmol, 57%)

^{1}H NMR (500 MHz, $CDCl_3$): δ 7.93 (s, 1H), 7.75 (s, 1H), 7.71 (s, 1H), 7.39 – 7.33 (m, 3H), 7.30 – 7.26 (m, 2H), 5.53 (s, 2H), 5.15 (s, 2H), 4.80 (hept, *J* = 6.8 Hz, 1H), 4.25 (q, *J* = 7.1 Hz, 2H), 3.81 – 3.75 (m, 6H), 1.58 (d, *J* = 6.8 Hz, 6H), 1.29 (t, *J* = 7.2 Hz, 3H). **^{13}C NMR (126 MHz, $CDCl_3$):** δ 166.41, 134.90, 129.24, 128.83, 128.17, 125.71, 124.09, 121.57, 62.52, 54.30, 53.09, 51.01, 47.25, 47.23, 47.11, 23.15, 14.21. **HRMS (ESI) (m/z):** $[M+H]^+$ calcd. for $C_{23}H_{31}N_{10}O_2$, 479.26260; found $[M+H]^+$: 479.2641 and $[M+Na]^+$: 501.24454

4-(((1-benzyl-1H-1,2,3,triazol-4-yl)methyl)amino)-1-isopropyl-1H-1,2,3,triazol-4-yl)methyl)-1H-1,2,3,-triazol-1-yl)methyl carboxylic acid (12c)

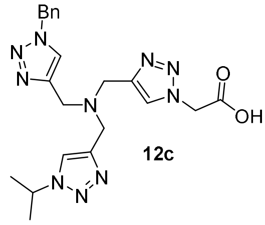

12c

White solid (60.4 mg, 0.134 mmol, 78%)

^{1}H NMR (500 MHz, $CDCl_3$): δ 10.14 (s, 1H), 8.52 (s, 1H), 8.35 (d, *J* = 14.5 Hz, 2H), 7.38 – 7.27 (m, 5H), 5.56 (s, 2H), 5.18 (s, 2H), 4.89 – 4.77 (m, 1H), 4.37 (d, *J* = 14.5 Hz, 6H). **^{13}C NMR (126 MHz, $CDCl_3$):** δ 210.99, 174.42, 168.21, 136.28, 135.47, 134.22, 129.63, 129.37, 129.10, 128.32, 128.09, 125.94, 69.74, 54.72, 53.93, 51.32, 46.66, 46.24, 31.87, 29.39, 20.55, 14.25. **HRMS (ESI) (m/z):** $[M+H]^+$ calcd. for $C_{21}H_{27}N_{10}O_2$, 451.23130; found $[M+H]^+$: 451.2322 and $[M+Na]^+$: 473.2142

Synthesis of the azide functionalized reagent: Methyl-4-(azidomethyl)benzoate

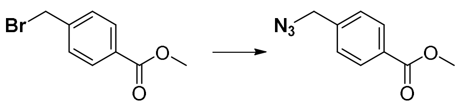

Methyl-4-(bromomethyl)benzoate (1.0 eq., 229.0 mg, 1.0 mmol) was dissolved in a 1:1 mixture of t-BuOH/H_2O (4 mL). Sodium azide (2.0 eq., 130.0 mg, 2.0 mmol) was added and was allowed to stir for 4 hours at room temperature. To isolate the desired product, the reaction mixture was diluted with $NaHCO_3$ aq. sat. (100 mL), brine (100 mL) and extracted with EtOAc (3x 100 mL). The combined organic layers were dried over $NaSO_4$, filtrated and concentrated under reduced pressure. Azide was obtained as clear oil (173.8 mg, 0.91 mmol, 91%).

^{1}H NMR (601 MHz, $CDCl_3$): δ 8.00 – 7.97 (m, 2H), 7.33 – 7.30 (m, 2H), 4.34 (s, 2H), 3.85 (s, 3H). **^{13}C NMR (151 MHz, $CDCl_3$):** δ 166.76, 140.53, 130.31, 128.08, 54.42, 52.31. Data in agreement with literature.[7]

2-(4-((bis((1-isopropyl-1H-1,2,3,triazol-4-yl)methyl)amino)methyl)-1H-1,2,3,-triazol-1-yl)methyl benzoate (11d)

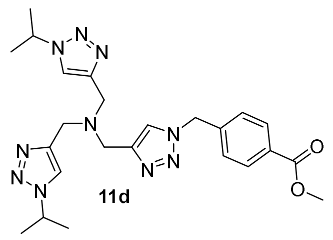

11d

White solid (168.1 mg, 0.34 mmol, 41%) – 363-f2

^{1}H NMR (500 MHz, CDCl$_3$): δ 8.03 (d, *J* = 8.5 Hz, 3H), 7.33 (d, *J* = 8.4 Hz, 2H), 5.60 (s, 2H), 4.82 (p, *J* = 6.8 Hz, 6H), 3.91 (s, 3H), 1.60 (d, *J* = 6.8 Hz, 12H). **^{13}C NMR (126 MHz, CDCl$_3$):** δ 166.54, 139.52, 130.74, 130.54, 130.24, 127.96, 53.92, 53.34, 52.40, 46.95, 23.13. **HRMS (ESI) (m/z):** $[M+H]^+$ calcd. for $C_{24}H_{33}N_{10}O_2$, 493.27825; found $[M+H]^+$: 493.2797 and $[M+Na]^+$: 515.2600

2-(4-((bis((1-isopropyl-1H-1,2,3,triazol-4-yl)methyl)amino)methyl)-1H-1,2,3,-triazol-1-yl)methyl-4-benzoic acid (12d)

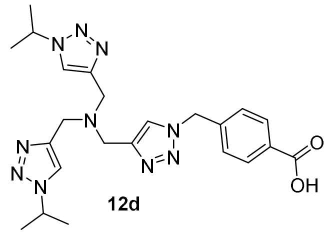

12d

White solid (88.4 mg, 0.185 mmol, 54%)

^{1}H NMR (500 MHz, CDCl$_3$): δ 10.14 (s, 1H), 8.46 (s, 1H), 8.38 (s, 2H), 7.91 (d, *J* = 8.5 Hz, 2H), 7.30 (d, *J* = 8.5 Hz, 2H), 5.63 (s, 2H), 4.84 (h, *J* = 6.8 Hz, 2H), 4.37 (d, *J* = 4.7 Hz, 6H), 1.60 (d, *J* = 6.8 Hz, 12H). **^{13}C NMR (126 MHz, CDCl$_3$):** δ 168.39, 139.27, 136.94, 135.67, 130.89, 130.53, 128.30, 127.97, 125.76, 54.10, 53.87, 46.46, 46.39, 23.02. **HRMS (ESI) (m/z):** $[M+H]^+$ calcd. for $C_{23}H_{31}N_{10}O_2$, 479.26260; found $[M+H]^+$: 479.2640 and $[M+Na]^+$: 501.2443

Synthesis of the *tert*-Butyl 3-(2-(2-(2-azidoethoxy)ethoxy)ethoxy)propanoate

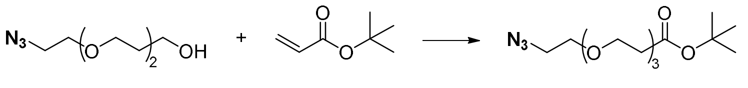

Synthesis towards obtaining *tert*-Butyl 3-(2-(2-(2-azidoethoxy)ethoxy)ethoxy)propanoate was performed in similar synthetic fashion as described by Ursuegui et al.[8] The data is in agreement as reported by Edem et al.[9]

Clear oil (385.5 mg, 1.27 mmol, 79%)

^{1}H **NMR (500 MHz, $CDCl_3$):** δ 3.73 – 3.59 (m, 14H), 3.38 (t, *J* = 5.1 Hz, 2H), 2.50 (t, *J* = 6.6 Hz, 2H), 1.44 (s, 9H). ^{13}C **NMR (126 MHz, $CDCl_3$):** δ 171.04, 80.64, 70.86, 70.81, 70.75, 70.52, 70.18, 67.05, 50.84, 36.43, 28.23.

2-(4-((bis((1-isopropyl-1H-1,2,3,triazol-4-yl)methyl)amino)methyl)-1H-1,2,3,-triazol-1-yl)-*tert*-butyl-3-(2-(2-(2-azidoethoxy)ethoxy)ethoxy)propanoate (11e)

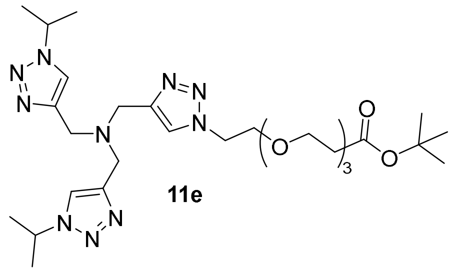

11e

Clear oil, (maximal yield: 276.98 mg, 0.458 mmol)

HPLC-MS (ESI): 2 µL injection, gradient 5 to 95% MeCN in H_2O, 5 minute run; **product rt:** 2.906 min, **(m/z):** $[M]^+$ calcd. for $C_{28}H_{48}N_{10}O_5$, 604.38, found $[M+H]^+$ 605.1 and $[M+Na]^+$ 627.0

* directly used in the next step to obtain product **12e**

2-(4-((bis((1-isopropyl-1H-1,2,3,triazol-4-yl)methyl)amino)methyl)-1H-1,2,3,-triazol-1-yl) 3-(2-(2-(2-azidoethoxy)ethoxy)ethoxy)propanoic acid (12e)

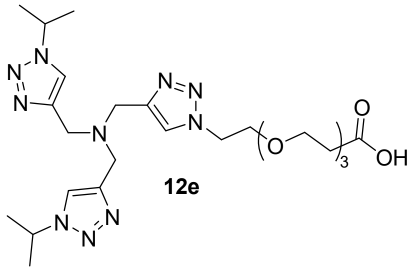

12e

Clear oil (66.1 mg, 0.120 mmol, 26% over 2-steps)

^{1}H **NMR (500 MHz, $CDCl_3$):** δ 10.14 (s, 1H), 8.39 (s, 1H), 8.34 (s, 2H), 4.84 (hept, *J* = 6.8 Hz, 2H), 4.64 – 4.60 (m, 2H), 4.38 (s, 2H), 4.33 (s, 4H), 3.90 (t, 2H), 3.75 (t, *J* = 5.8 Hz, 2H), 3.63 – 3.54 (m, 8H), 2.56 (t, *J* = 5.8 Hz, 2H), 1.62 (d, *J* = 6.8 Hz, 12H). ^{13}C **NMR (126 MHz, $CDCl_3$):** δ 173.87, 128.88, 125.63, 71.16, 70.52, 69.47, 66.84, 53.80, 50.84, 46.80, 46.12, 35.33, 23.03. **HRMS (ESI) (m/z):** $[M+H]^+$ calcd. for $C_{24}H_{41}N_{10}O_5$, 549.32559; found $[M+H]^+$: 549.3253 and $[M+Na]^+$: 571.3062

^{1}H NMR spectrum of compound 8a. Spectra were taken in $CDCl_3$-d_1 at 500 MHz

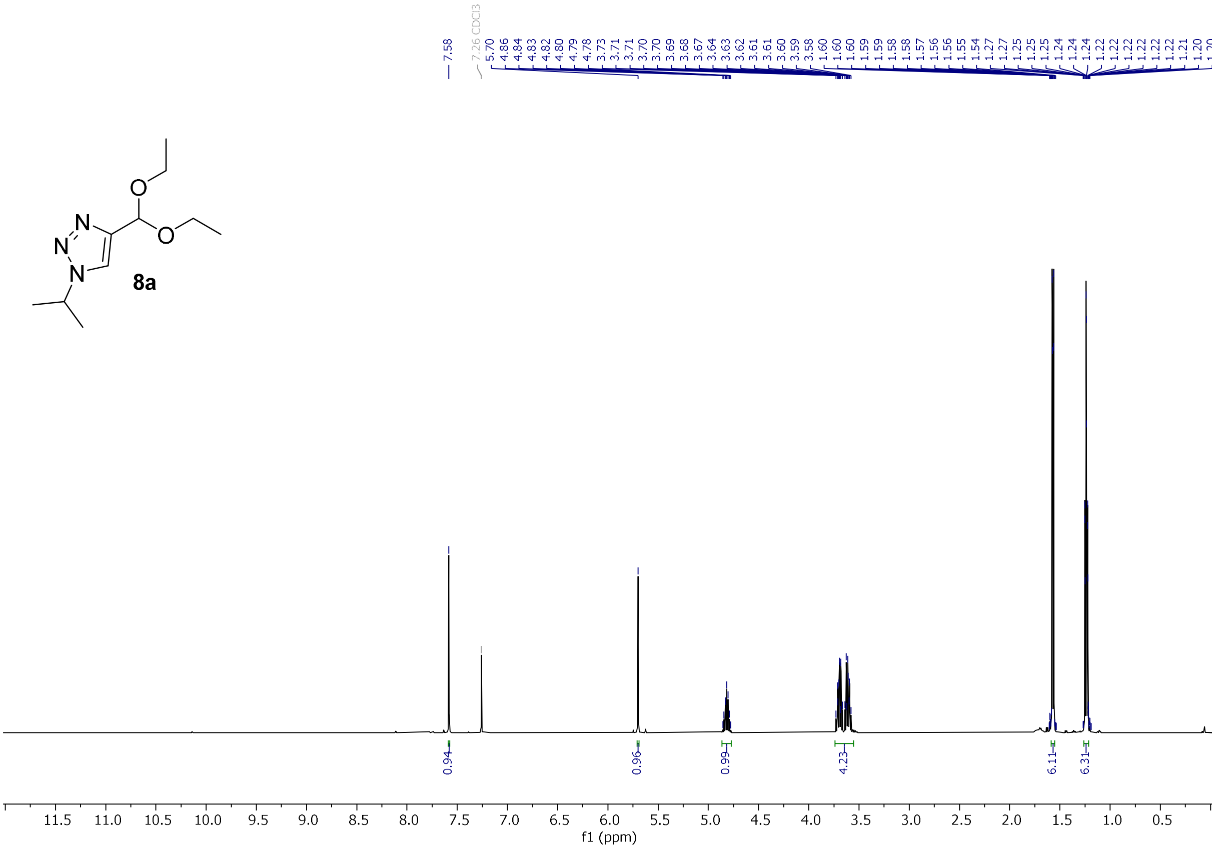

^{1}H NMR spectrum of compound 4a. Spectra were taken in $CDCl_3$-d_1 at 500 MHz

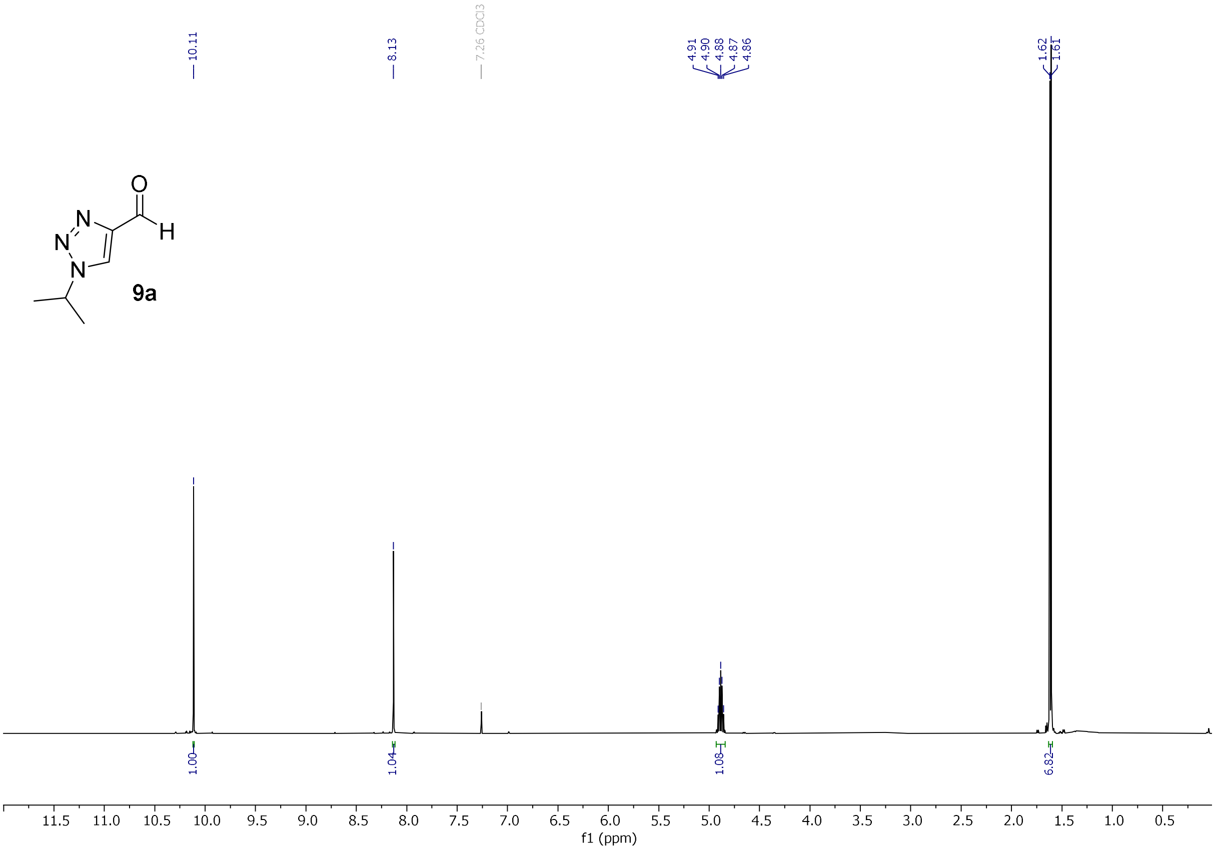

HPLC-MS analysis of compound 10d, rt: 2.906 min, **ESI (m/z):** $[M]^+$ calcd. for $C_{13}H_{14}N_4$, 226.12, found $[M]^+$ 226.8 $[M+Na]^+$ 249.9 & **rt:** 1.878 min, **(m/z):** found $[M]^+$ 226.9 $[M+H]^+$ 227.9 $[M+Na]^+$ 248.9

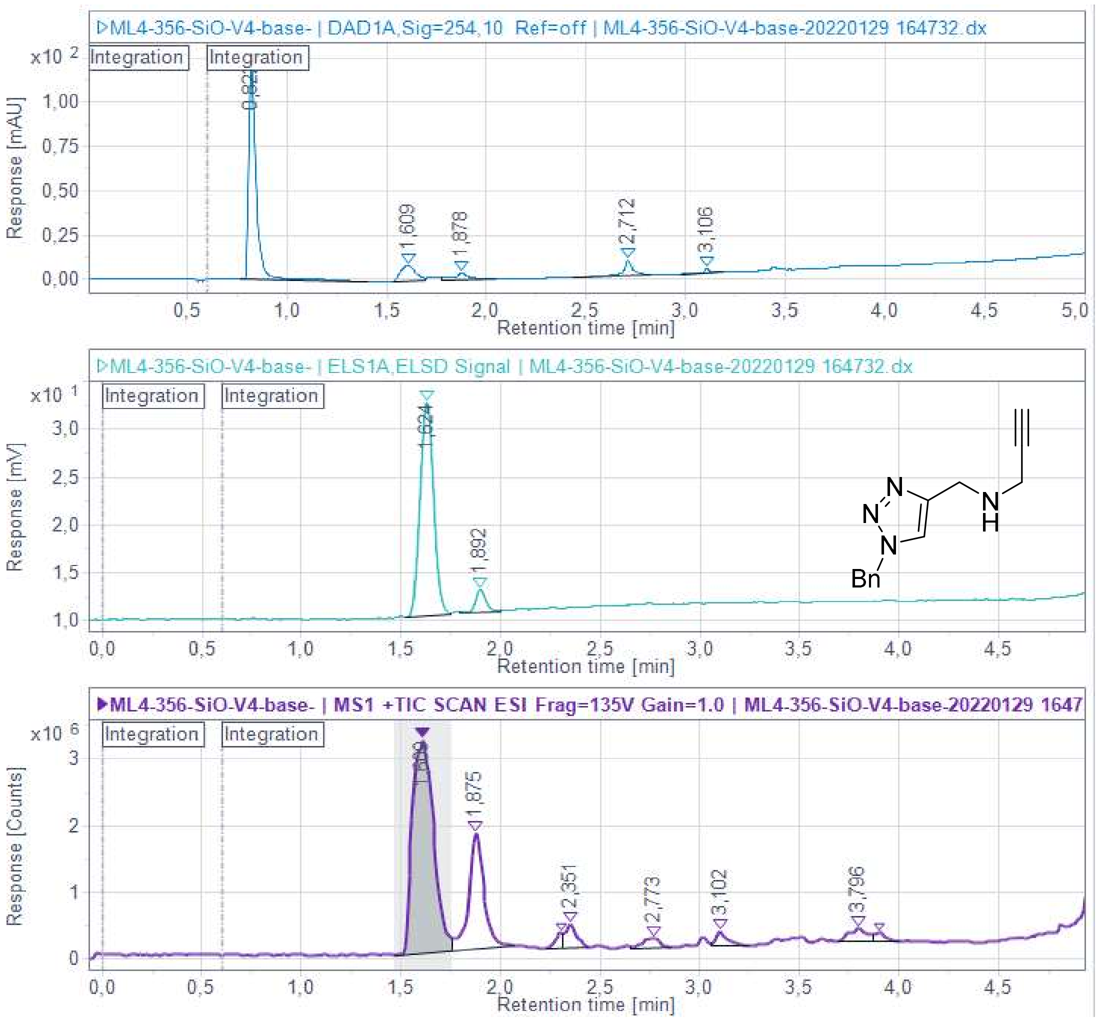

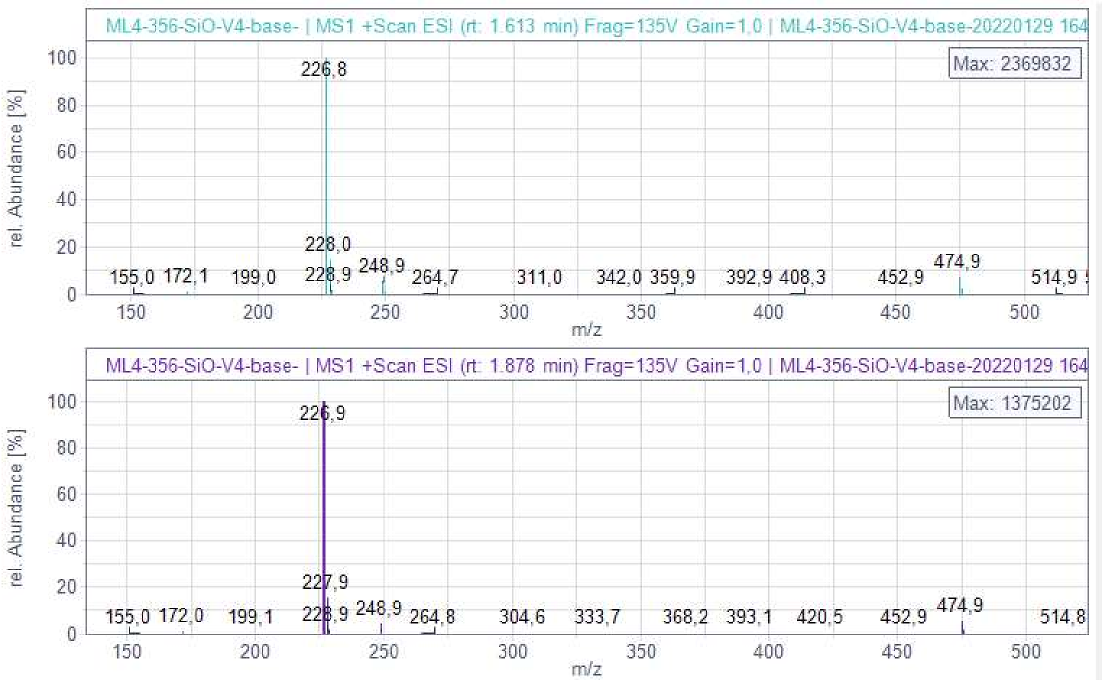

^{1}H NMR spectrum of compound 10a. Spectra were taken in $CDCl_3$-d_1 at 500 MHz

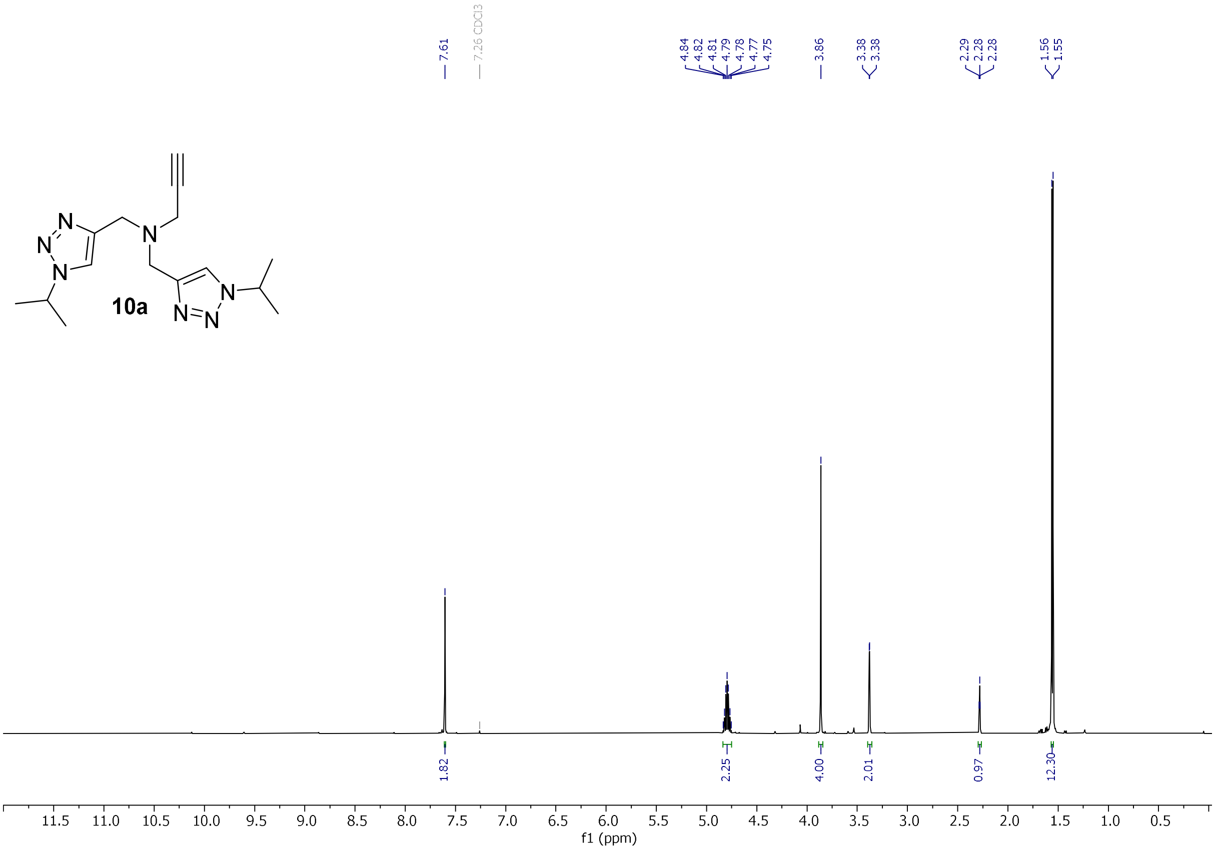

^{13}C NMR spectrum of compound 10a. Spectra were taken in $CDCl_3$-d_1 at 126 MHz

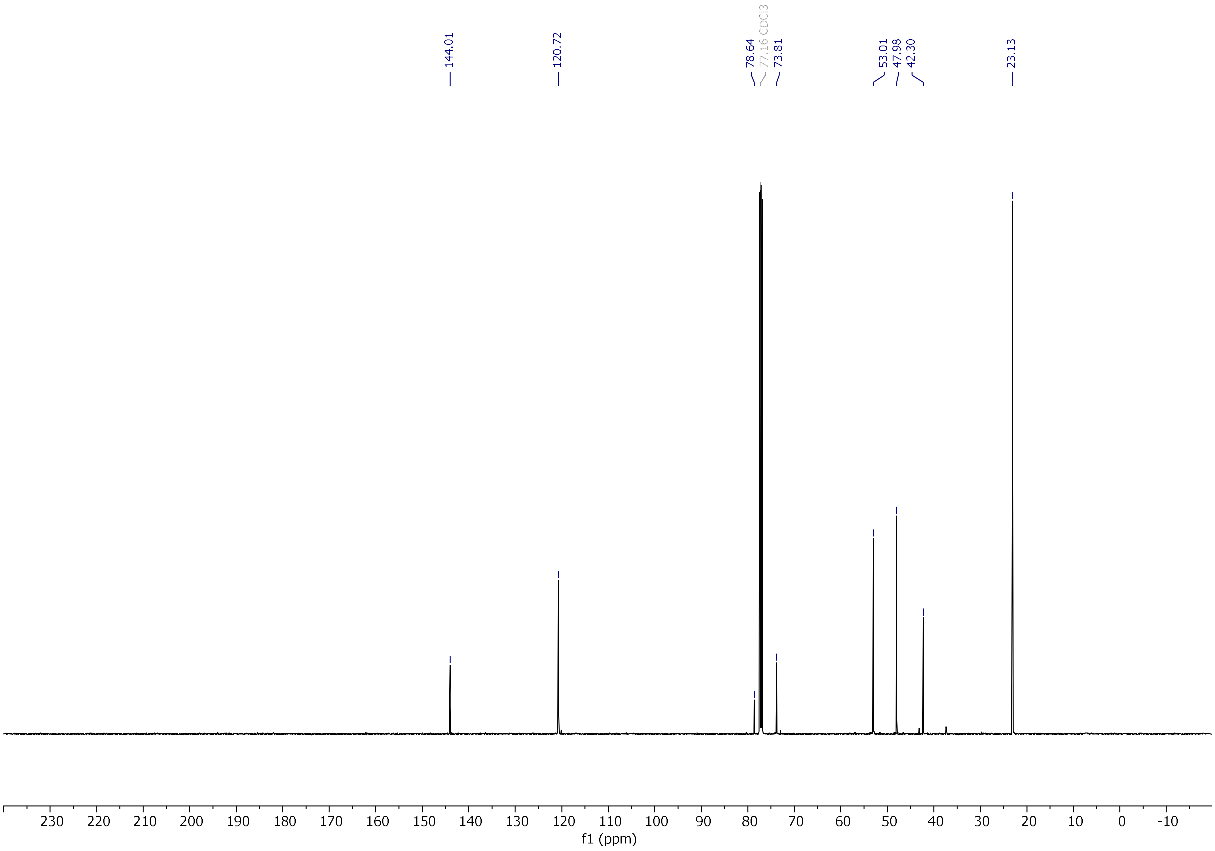

^{1}H NMR spectrum of compound 10c. Spectra were taken in $CDCl_3$-d_1 at 500 MHz

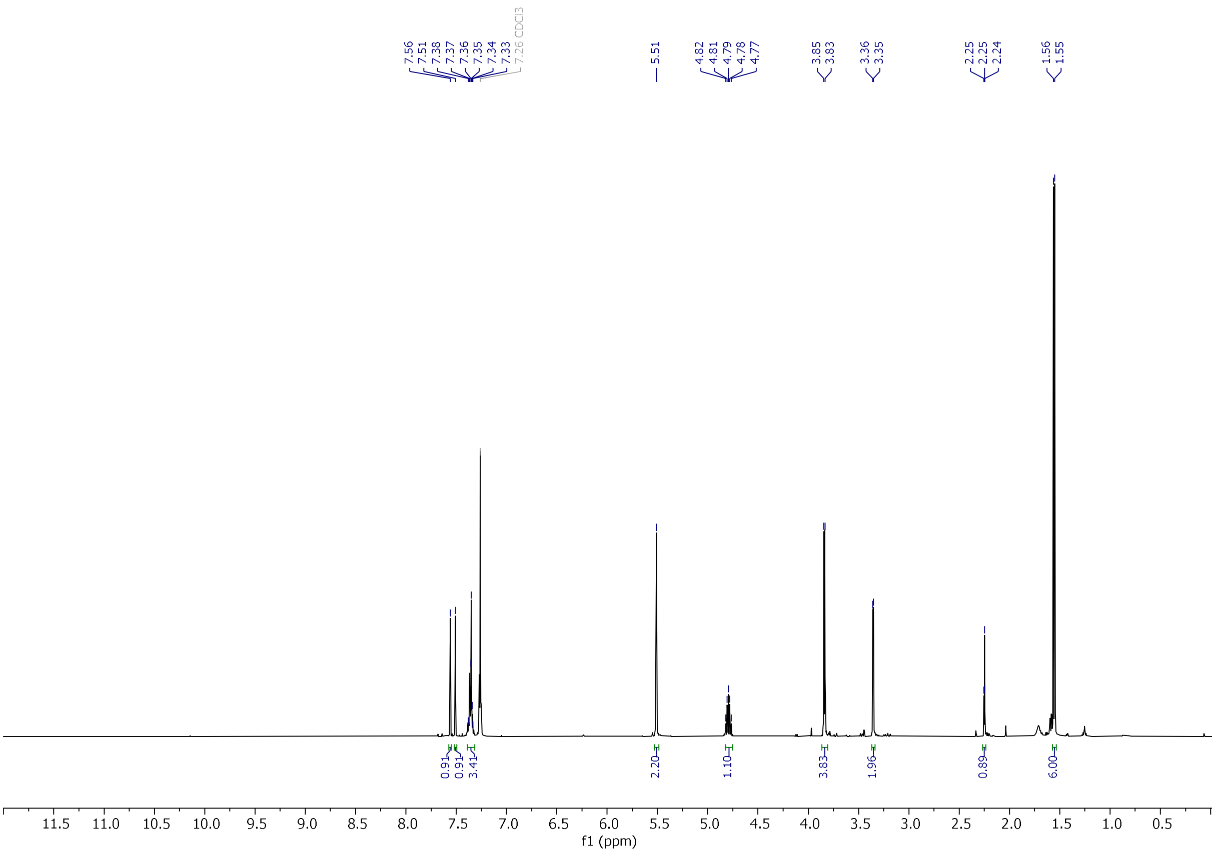

^{13}C NMR spectrum of compound 10c. Spectra were taken in $CDCl_3$-d_1 at 126 MHz

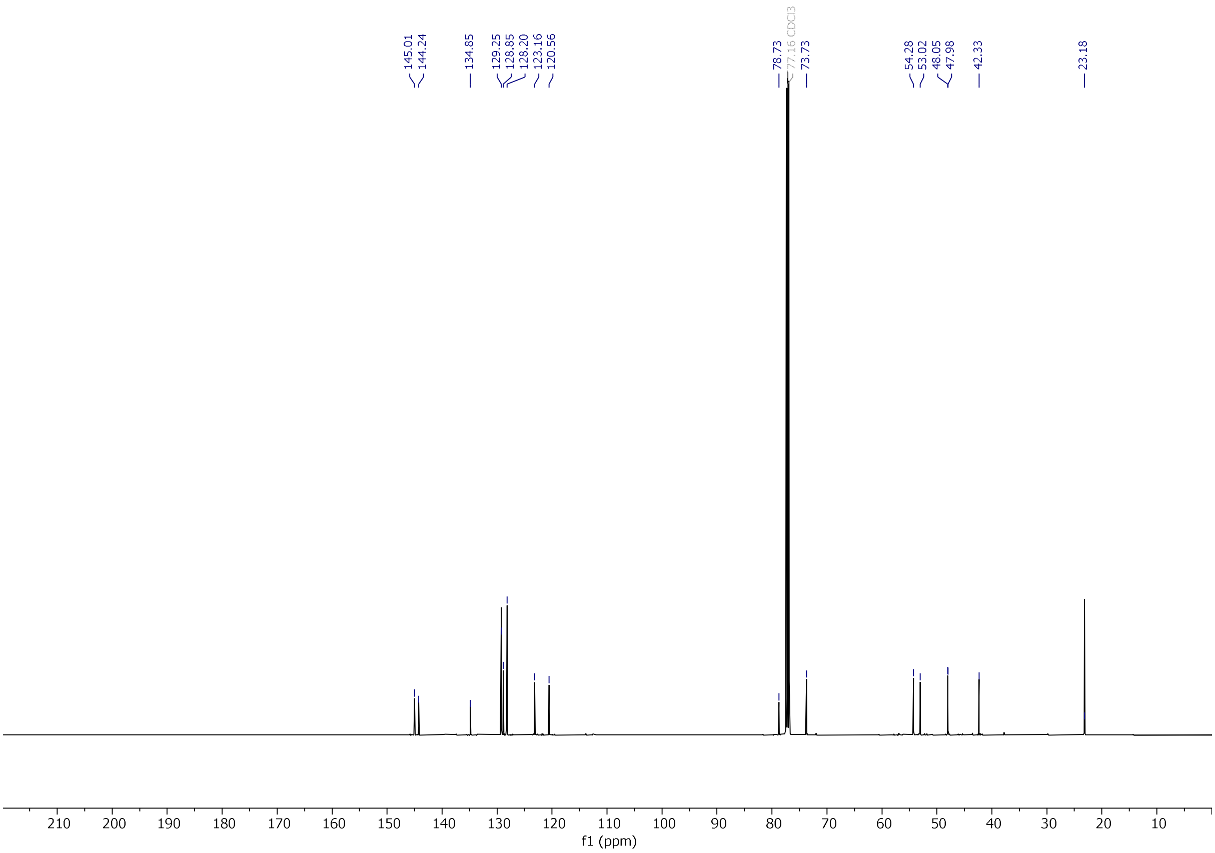

^{1}H NMR spectrum of compound 12a. Spectra were taken in $CDCl_3$-d_1 at 500 MHz

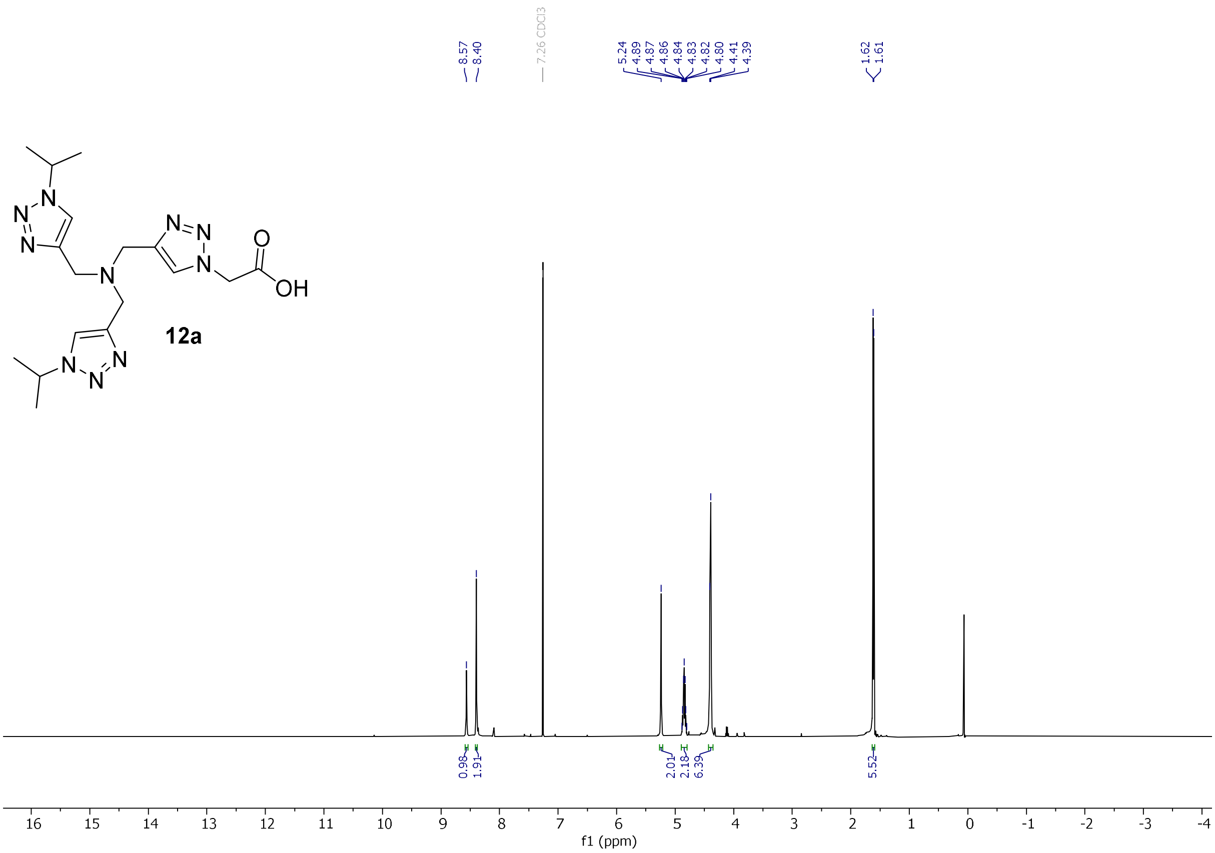

^{13}C NMR spectrum of compound 12a. Spectra were taken in $CDCl_3$-d_1 at 126 MHz

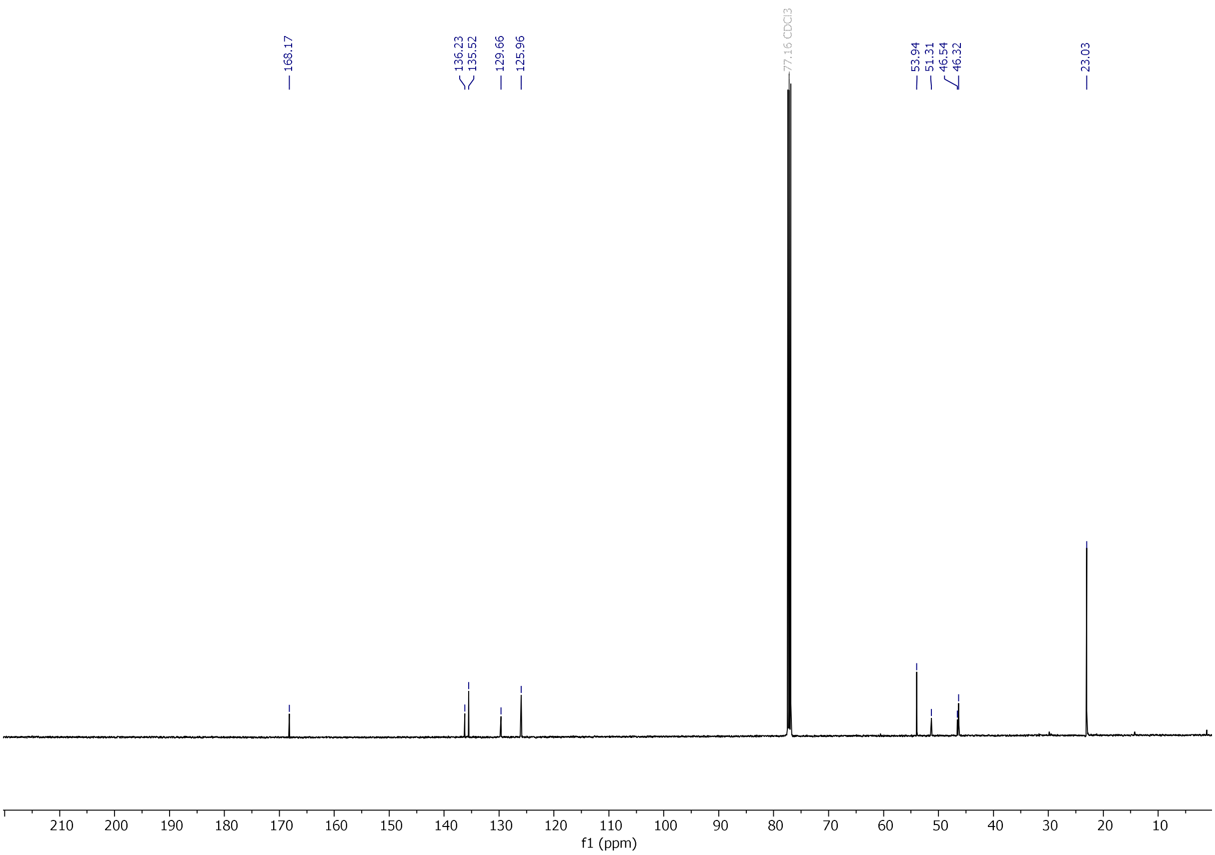

^{1}H NMR spectrum of compound 12b. Spectra were taken in $CDCl_3$-d_1 at 500 MHz

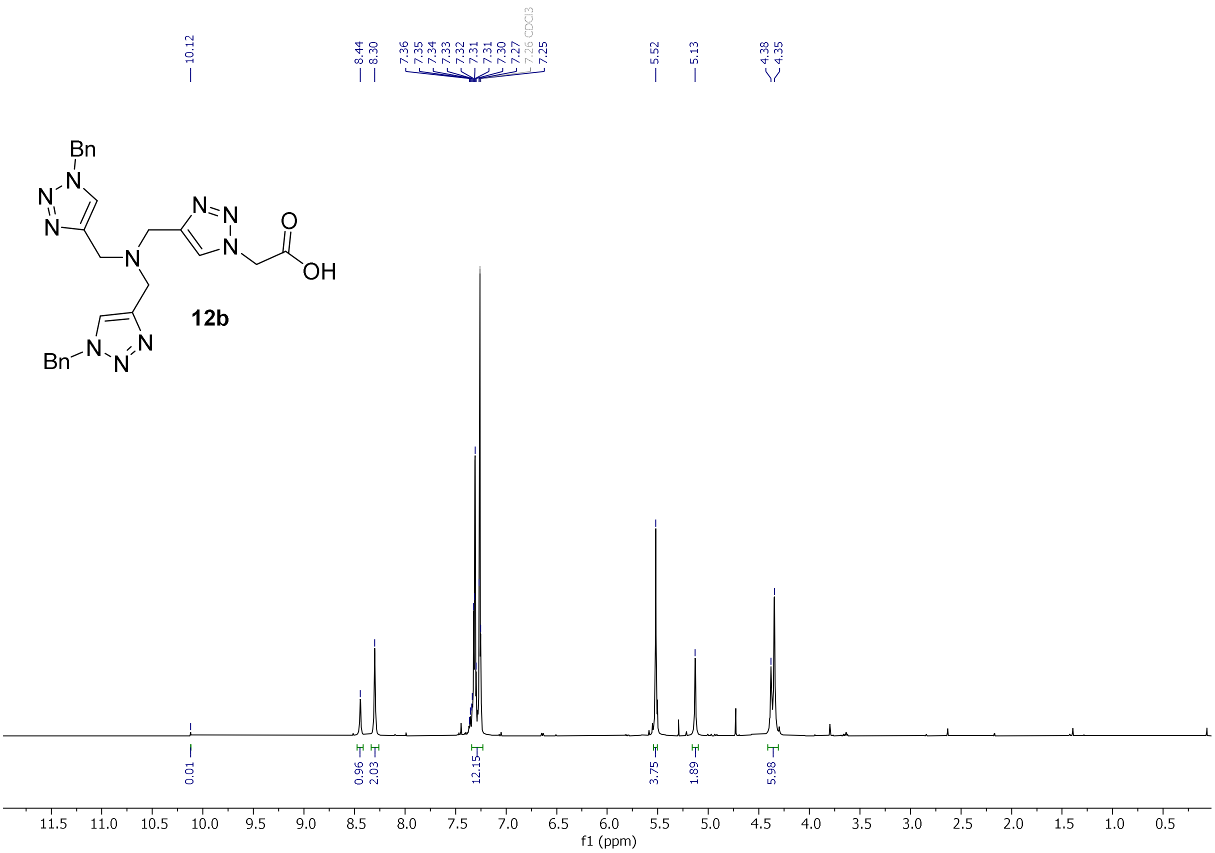

^{13}C NMR spectrum of compound 12b. Spectra were taken in $CDCl_3$-d_1 at 126 MHz

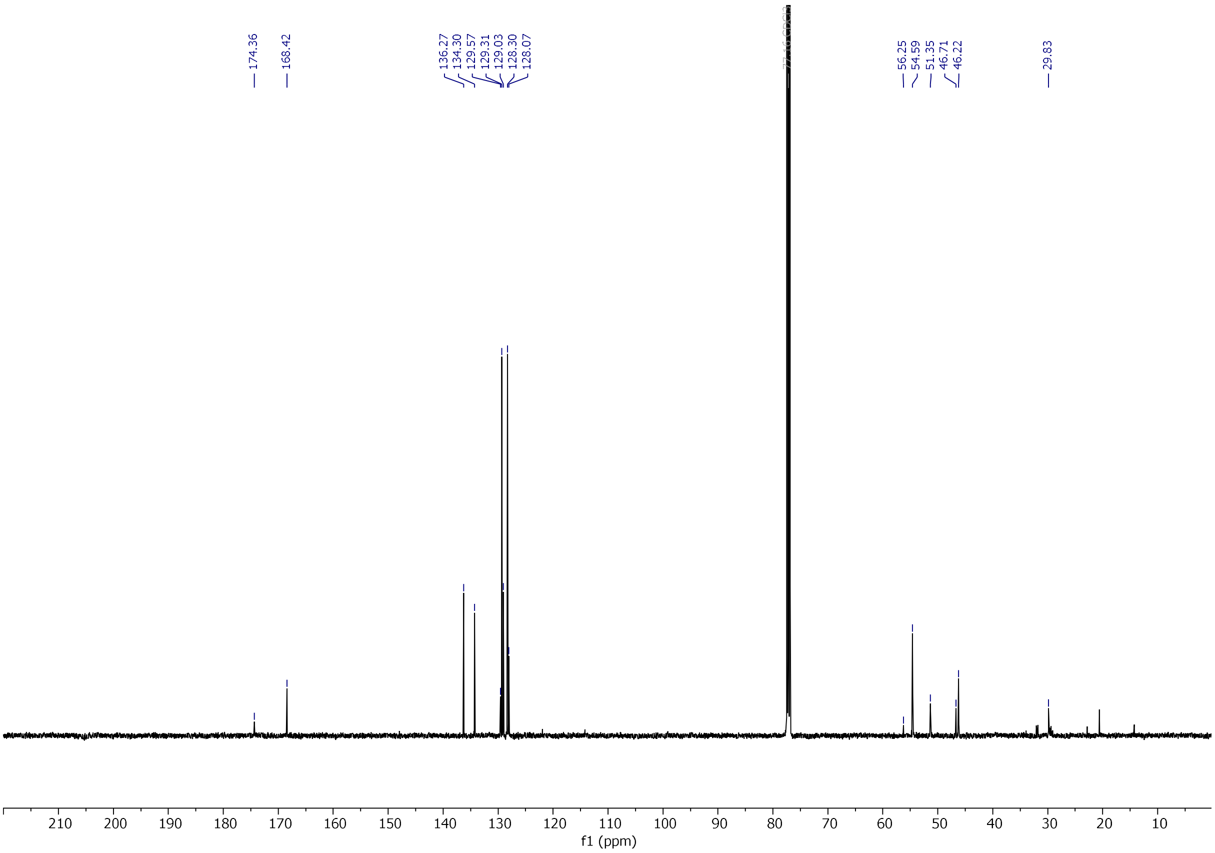

^{1}H NMR spectrum of compound 12c. Spectra were taken in $CDCl_3$-d$_1$ at 500 MHz

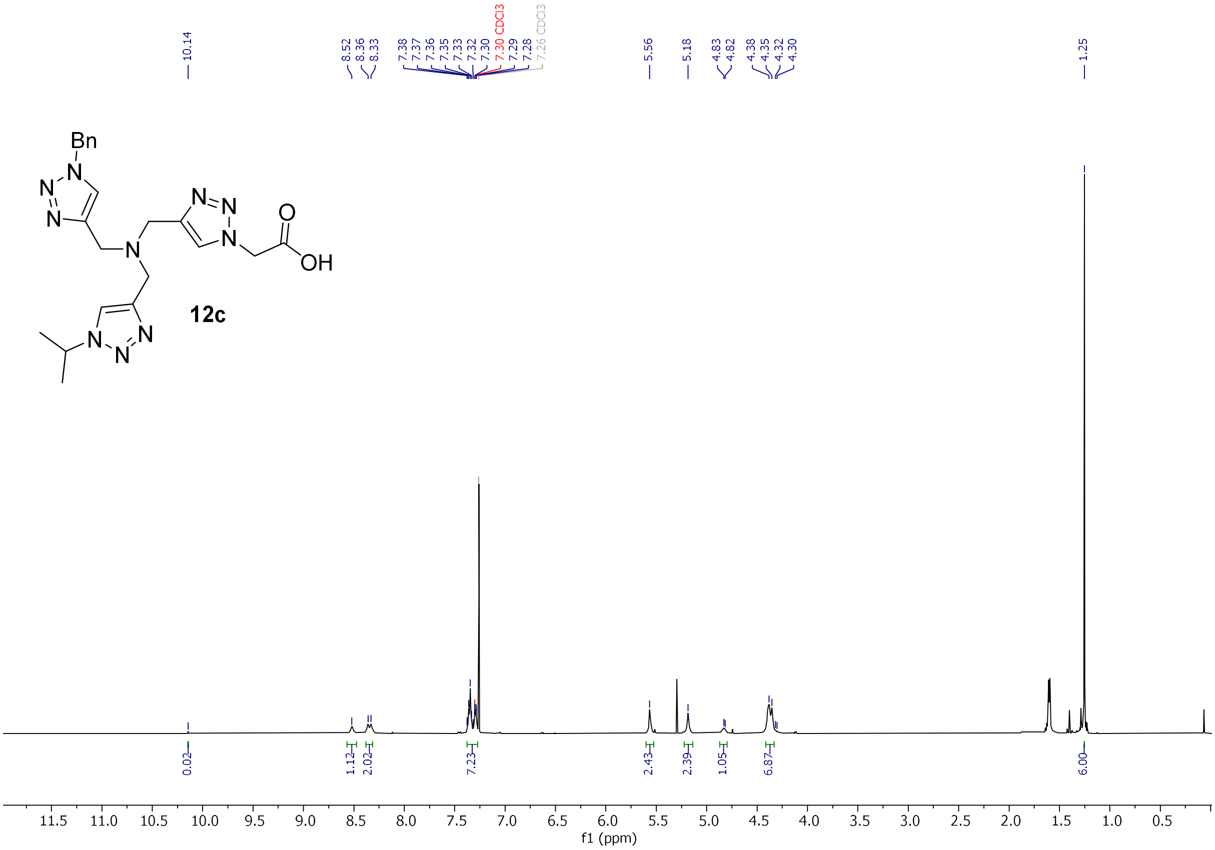

^{13}C NMR spectrum of compound 12c. Spectra were taken in $CDCl_3$-d$_1$ at 126 MHz

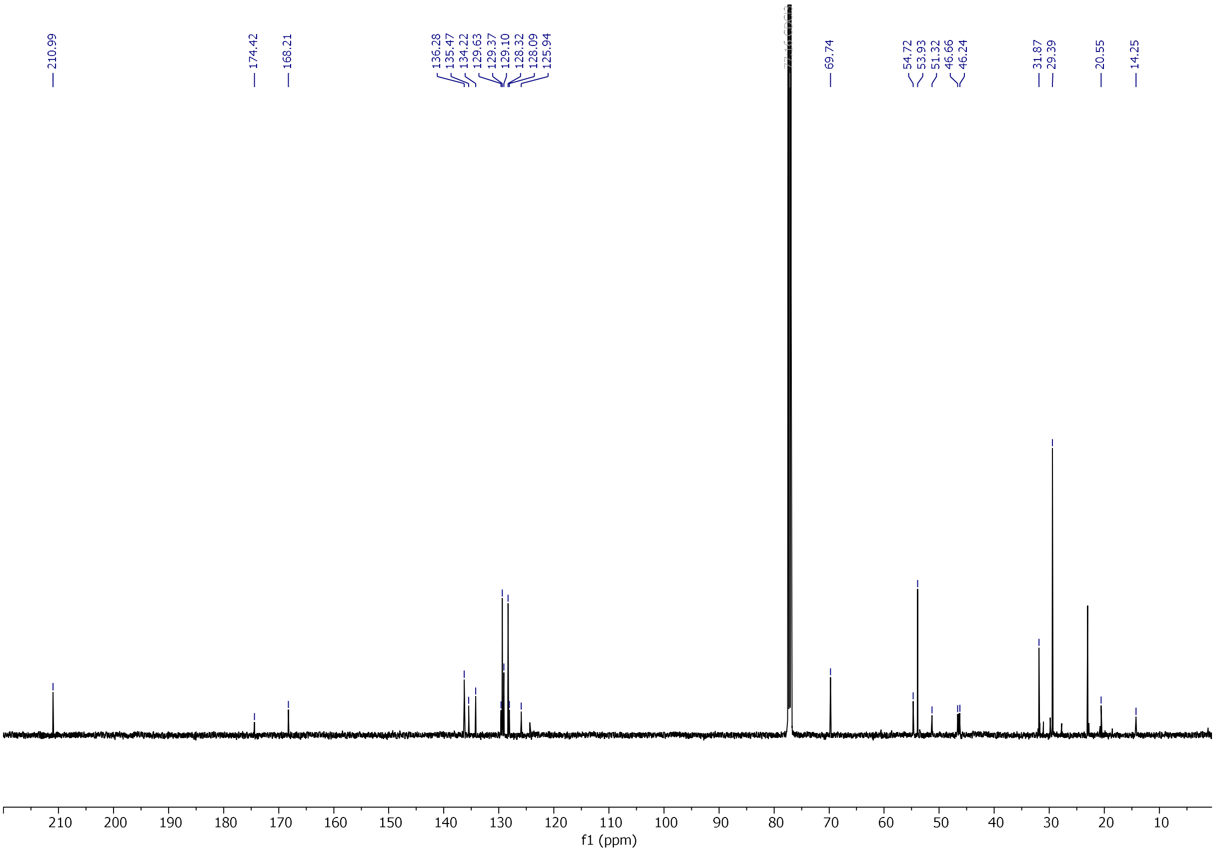

^{1}H NMR spectrum of compound 12d. Spectra were taken in $CDCl_3$-d_1 at 500 MHz

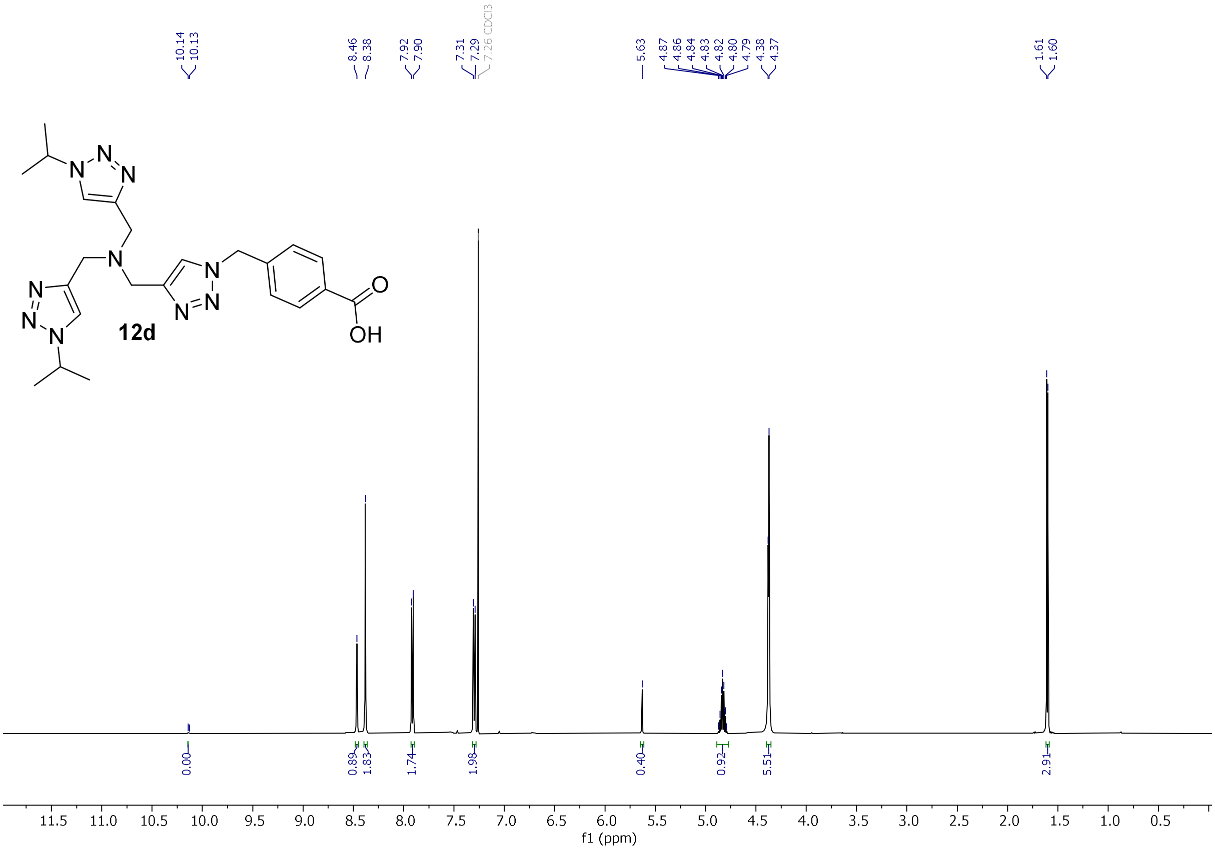

^{13}C NMR spectrum of compound 12d. Spectra were taken in $CDCl_3$-d_1 at 126 MHz

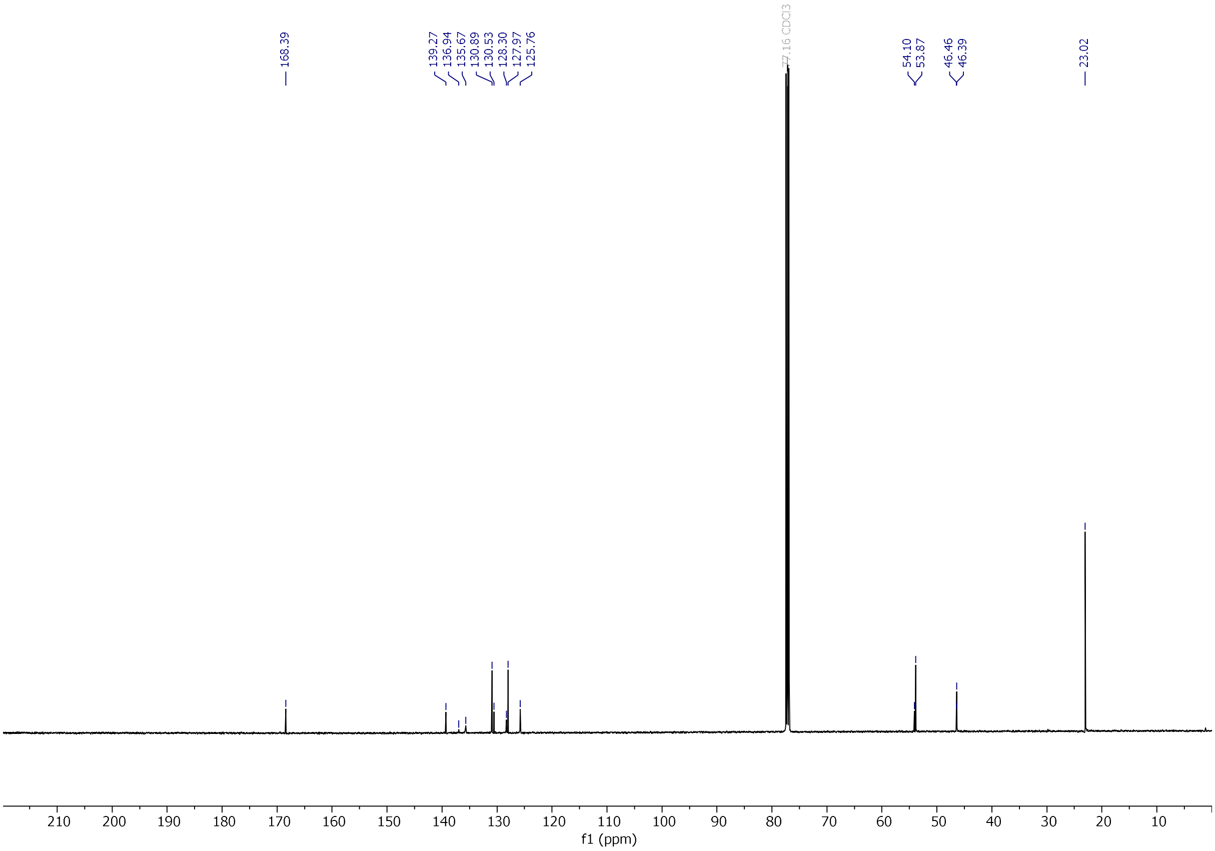

HPLC-MS analysis of compound 11e, rt: 2.906 min, **ESI (m/z):** $[M]^+$ calcd. for $C_{28}H_{48}N_{10}O_5$, 604.38, found $[M+H]^+$ 605.1 and $[M+Na]^+$ 627.0

* no DAD signal detected as compound **11e** is not UV active

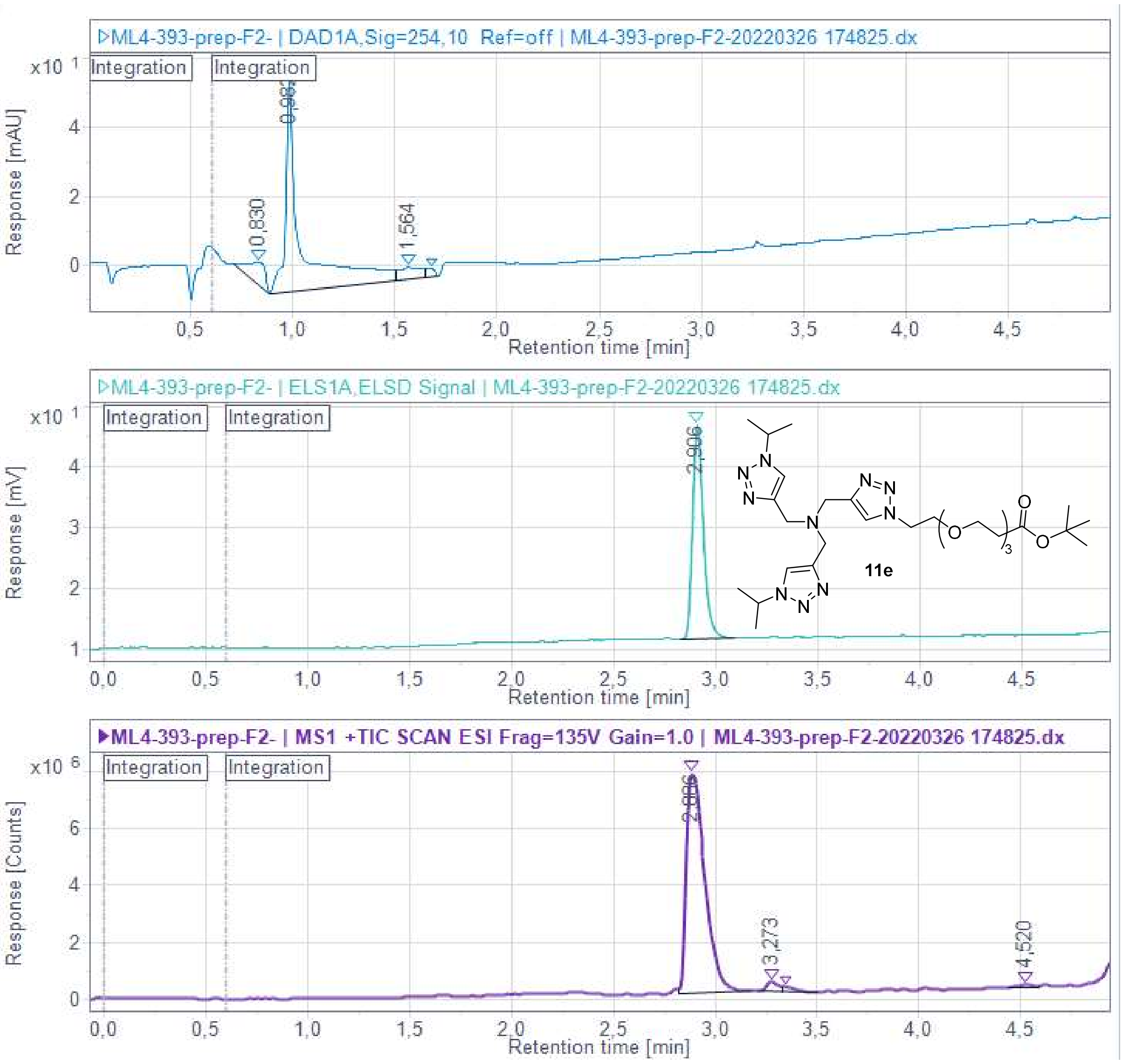

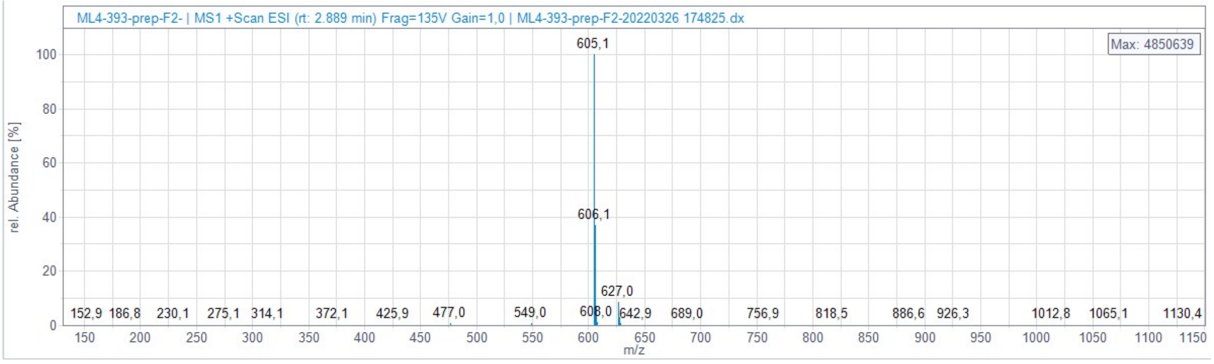

^{1}H NMR spectrum of compound 12e. Spectra were taken in $CDCl_3$-d_1 at 500 MHz

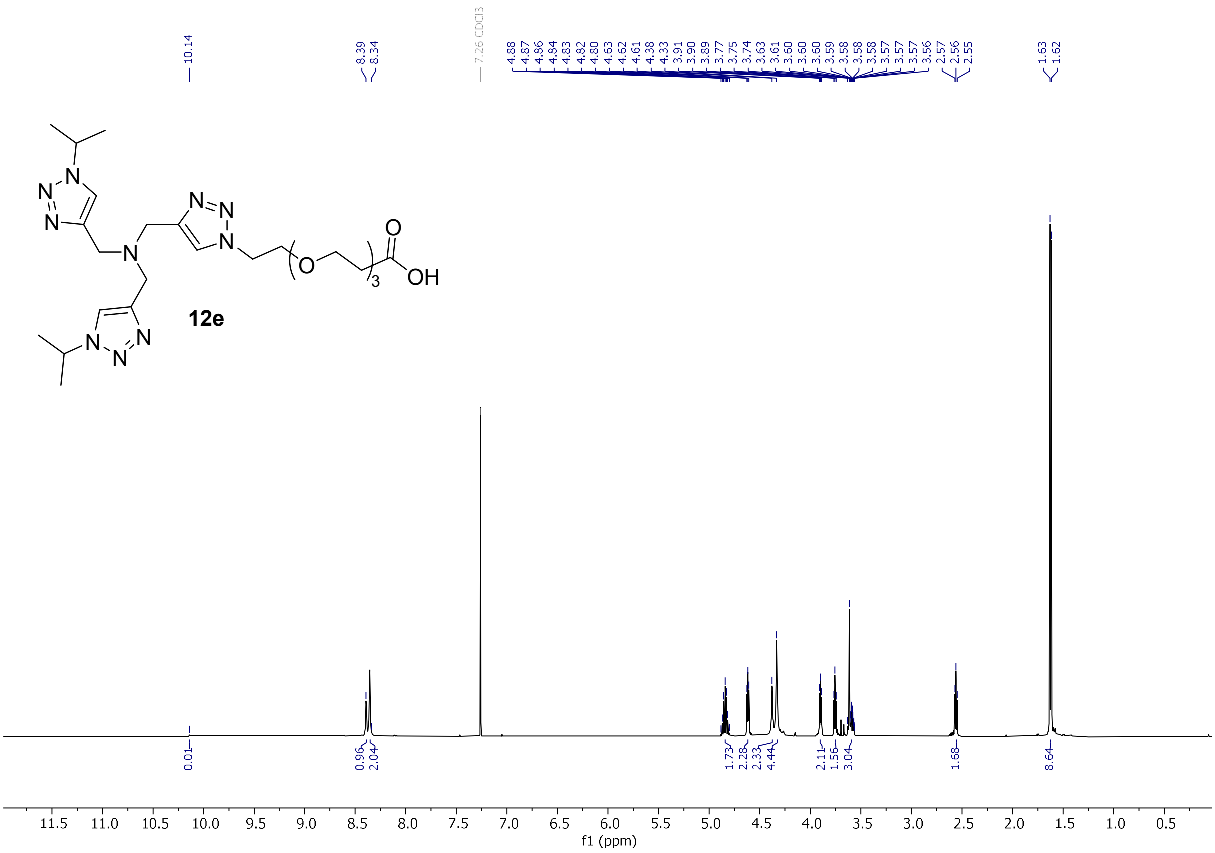

^{13}C NMR spectrum of compound 12e. Spectra were taken in $CDCl_3$-d_1 at 126 MHz

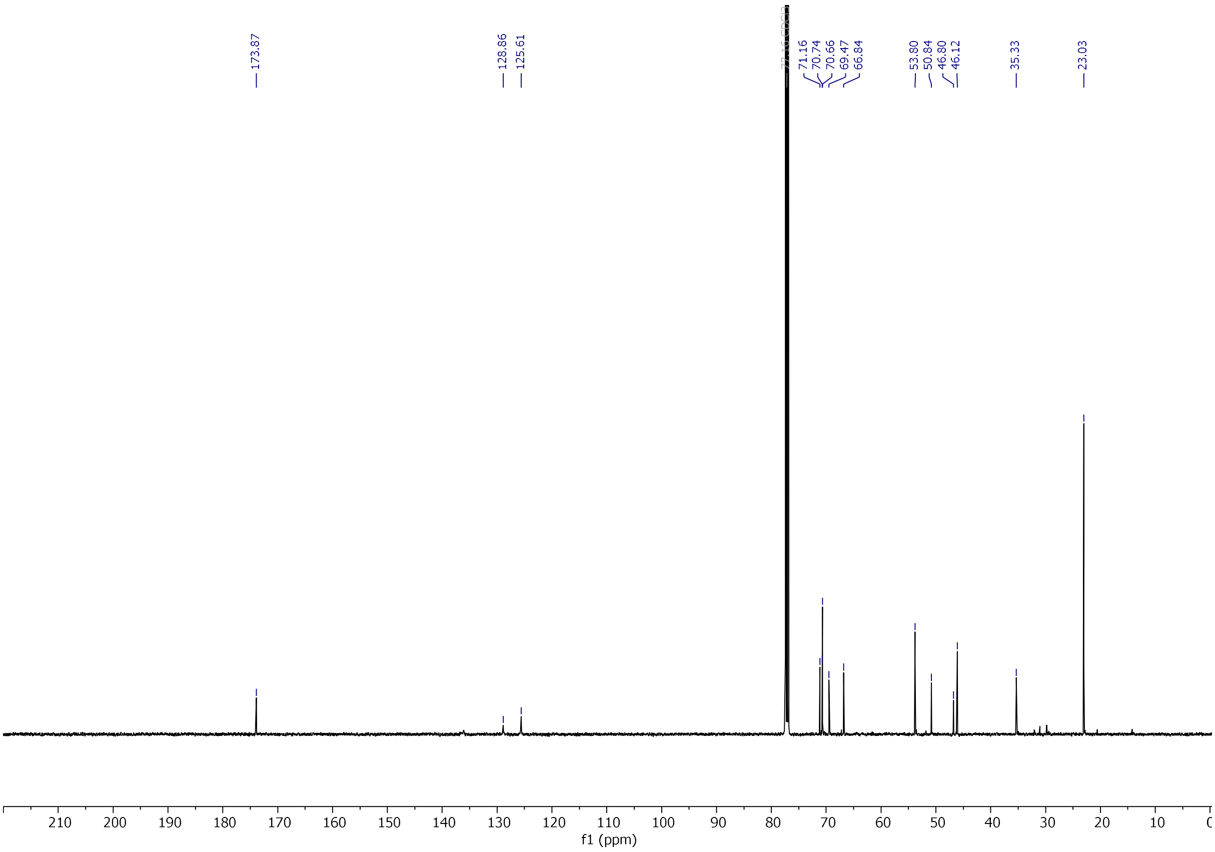

4. Copper loading onto ligand-functionalised resins

The procedure is an adapted version of the method reported by Fokin et al.[5]

Step 1: Tris-triazole ligand functionalisation of Tentagel-NH_2 resin by peptide coupling

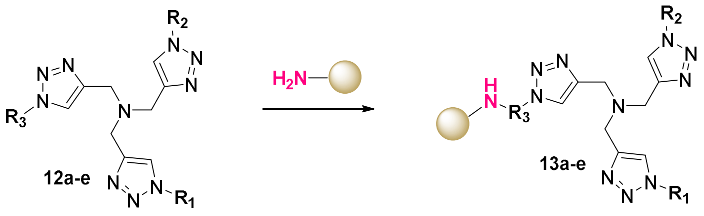

a) R_1=iPr, R_2=iPr, R_{3b}=CH_2CO
b) R_1=Bn, R_2=Bn, R_{3b}=CH_2CO
c) R_1=Bn, R_2=iPr, R_{3b}=CH_2CO
d) R_1=iPr, R_2=iPr, R_{3b}=BnCO
e) R_1=iPr, R_2=iPr, R_{3b}=$CH_2CH_2(OCH_2CH_2)_3CO$

The commercially purchased Tentagel-NH_2 (HL 12902) resin 0.46 µmol/g (1.0 eq., 42.7 mg, 19.6 µmol) from Rapp Polymer GmbH, were loaded into a polypropylene tube of 2mL, equipped with a plastic frit and placed onto a closed Solid Phase Extraction Vacuum Manifold system. The resins were swelled with DCM (3 x 1.5 mL) and DMF (3 x 1.5 mL). In a separate glass vial, the acid **12a-e** (2.0 eq., 14.8 mg, 36.8 µmol) was dissolved in DMF (1.0 mL) and activated by addition of DIPEA (1.0 eq., 3.2 µL, 18.4 µmol), Oxyma (1.9eq., 4.96 mg, 35.0 µmol) and DIC (1.9 eq., 5.5 µL, 35.0 µmol). After 5 minutes, the solution was added into the polypropylene tube containing the swollen resin, closed off and placed in a rotation wheel to rotate overnight.

The monitoring of the peptide coupling was done via the Kaiser test, the reaction was stirred until the test results were negative (no blue stain of the light yellow resin). Next, the resins were washed by use of the SPE Vacuum filtration with; 1:1 DMF/H_2O (1,5 mL), DMF (3 x 1,5 mL), MeOH (3 x 1,5 mL), DCM (3 x10 mL) and then dried under high vacuum at room temperature overnight, resulting into yellow sticky resins (total weight +/-46mg).

Step 2: Copper loading onto the ligand-functionalised resin and ascorbate reduction into Cu(I)

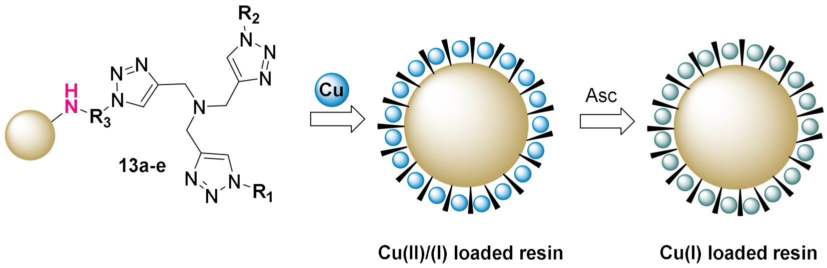

The ligand functionalised Tentagel resin (1.0 eq., 26 mg, 11.9 µmol) was loaded into a polypropylene tube of 2mL, equipped with a plastic frit and placed onto a closed SPE Vacuum Manifold filtration slot. The ligand-resins were swollen with DCM (3 x 1.5 mL). A solution of ($Cu(MeCN)_4$ BF_4 or) $Cu(MeCN)_4$ PF_6 (3.0 eq., 13.4 mg, 35.9 µmol) in 10% MeCN/DCM (2.0 mL) was added and vortexed for 2 minutes.

The solution was filtered by ultra-filtration and washed with MeOH (3 x 1.0 mL). Followed by the copper reduction with a solution of sodium ascorbic acid (10 eq., 24.0 mg, 119.6 μmol) in MeOH (2.0 mL) and vortexed for 2 minutes.

Then, the resin was washed with MeOH (1.5 mL), then DCM (1.5 mL) repeated the cycle 3 times accordingly. Next, the copper loaded resin were dried under high vacuum at room temperature for 24 hours. The high vacuum drying influences a colour change of the resin from dark to light green.

Step 3: copper content measurement

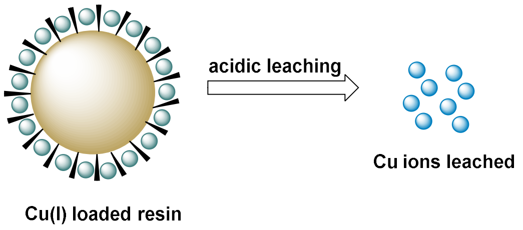

The copper (Cu) content (%, w/w) was determined by leaching the Cu loaded tris-triazole Tentagel resins (+/- 1.0 mg) in 3:1 ratio of HCl/HNO_3 (0.5 mL) for 2 hours. The solution was then diluted by adding 100 μL to 4.0 mL Milli-Q H_2O. The ICP-MS content measurements were performed in an Agilent 7500ce instrument**.** Table 1 of the manuscript contains the comparison of the theoretical maximal Cu versus the ICP-MS measured amount of Cu content, of isopropyl functionalised Tentagel resin variating in size and commercially purchased TBTA-Tentagel of 75 μm resin.

Table S1. Chemical and functional characterization of Cu(I)-microdevices.

Ligand / Resin size	Est. maximum loading capacity (μmol/mg)[a]	Exp.Cu-to-ligand loading ratio avg. (%)[a]	2-to-Res conversion with 0.5mg resin in PBS (%)[b]	2-to-Res conversion with 1.0mg resin in PBS (%)[b]
**14a / 10 μm**	0.236	32	24	46
**14a / 30 μm**	0.194	32	69	52
**14a / 75 μm**	0.391	43	82	79
**TBTA / 75 μm**	0.17	27	64	43

[a] The ICP-MS samples were prepared by leaching the Cu(I)-microdevices with a 3:1 $HCl:HNO_3$ solution and dilution to 2.5% v/v acid/H_2O sample solution. The copper content per microdevice was determined by calculating the maximal ligand loading and estimating the maximal copper coordination value at a 1:1 ratio.

[b] The uncaging percentage of 26, 21, 46, 17 μM prodye **2** (~ratio 1:1 and 1:2 = prodye **2** : maximal μmol Cu(I)/0.5 or 1 mg resin) by 0.5 & 1.0 mg Cu(I)-microdevices (17a/10 μm, 17a/30 μm, 17a/75 μm and comm. TBTA/75 μm) was determined in PBS in absence of NaAsc. Data in the table shows uncaging percentages after 120 min reactions.

Fluorogenic screening assays were performed to evaluate uncaging by the Cu(I)-resins. Figure S1 contains the results from the screening of the *iso*-propyl functionalised Tentagel resin with the sizes of 10, 30 and 75 µm versus the commercially purchased TBTA functionalised Tentagel resin 75 µm.

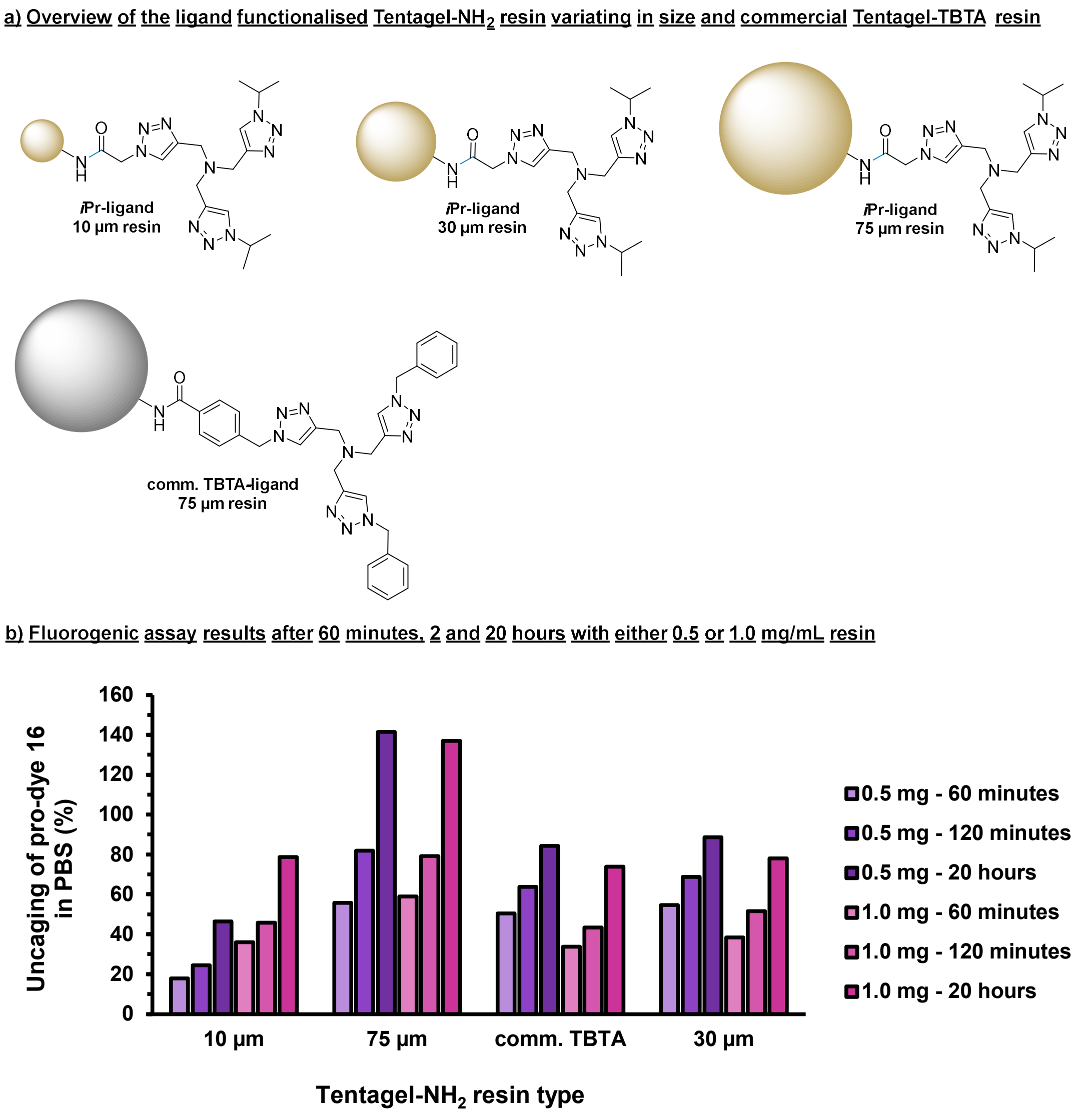

Figure S1: Fluorogenic uncaging assay mediated by the *iso*-propyl Cu(I) resin in different sizes versus TBTA-Tentagel.

5. Cu(I)-assemblable PROTACs.

General Procedure A

In a 10 mL vial was weighed the azide (0.046 mmol, 1.2eq.), the alkyne (0.040 mmol, 1.0eq.)., (+)-sodium l-ascorbate (1.58 mg, 0.008 mmol, 0.2 eq.), copper(II) sulfate powder (1.30 mg, 0.008 mmol, 0.2 eq.), and TBTA (7 mg, 0.013 mmol, 0.3 eq.) The reaction mixture was treated with DMF (0.5 mL) and 3−4 drops of water, and then the headspace of the vial was purged briefly with nitrogen and stirred at 80 °C for 1 h. The solvent was removed in vacuo and the solid was re-suspended in water. The aqueous phase was extracted with ethyl acetate. The organics were combined and dried over anhydrous $MgSO_4$, concentrated in vacuo and purified using flash chromatography.

Compound 17

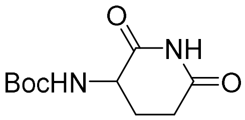

To a mixture of Boc-L-glutamine **15** (500 mg, 2.03 mmol, 1.0 eq) in THF (10 mL) was added 1,1′-carbonyldiimidazole (395 mg, 2.44 mmol, 1.2 eq) and a catalytic amount of 4-(dimethylamino)pyridine (1 mg). It was heated at reflux for 10 h until a clear solution formed. The solvent was removed in vacuo and the crude was dissolved in EtOAc (50 mL). The organic layer was washed with H_2O (15 mL), followed by brine (15 mL). The organic layer was dried with $MgSO_4$, the solvent removed *in vacuo* and purified using flash chromatography to give the title compound as a white solid (357 mg, 1.56 mmol, 77% yield).

^{1}H NMR (500 MHz, DMSO) δ 10.71 (s, 1H), 7.09 (d, J = 8.7 Hz, 1H), 4.25 – 4.17 (m, 1H), 2.73 – 2.66 (m, 1H), 2.48 – 2.43 (m, 1H), 1.96 – 1.85 (m, 2H), 1.38 (s, 9H). **13C NMR (126 MHz, DMSO) δ** 173.10, 172.65, 154.35, 78.31, 50.56, 31.64, 28.33, 24.76.

Compound 18

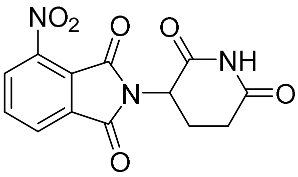

Compound **17** (250 mg, 1.1 mmol, 1.0 eq), phthalic anhydride (317 mg, 1.6 mmol, 1.5 eq), NaOAc (108 mg, 1.3 mmol, 1.2 eq) was dissolved im glacial AcOH (5 mL) and refluxed for 6 h. After cooling the

reaction mixture was poured into H_2O (20 mL) and the solid was collected by filtration, washed with H_2O (3 × 2 mL) and dried *in vacuo* to give the title compound as a grey solid (270 mg, 0.9 mmol, 81%).

^{1}H NMR (500 MHz, DMSO) δ 11.13 (s, 1H), 8.37 – 8.31 (m, 1H), 8.26 – 8.20 (m, 1H), 8.15 – 8.07 (m, 1H), 5.19 (dd, J = 5.4, 13 Hz, 1H), 2.94 – 2.83 (m, 1H), 2.65 – 2.57 (m, 1H), 2.56 – 2.44 (m, 1H), 2.12 – 2.03 (m, 1H). **^{13}C NMR (126 MHz, DMSO)** 172.77, 169.57, 165.29, 162.64, 144.58, 136.92, 133.15, 128.97, 127.40, 122.69, 49.59, 30.99, 21.89.

Compound 19

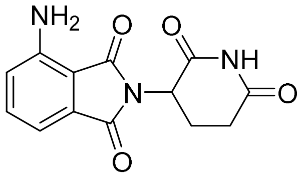

To a 10 mL microwave vial compound containing EtOH (1 mL), compound **18** (75 mg, 0.25 mmol, 1.0 eq) and SnCl2.H2O (279 mg, 1.24 mmol, 5.0 eq) was added. The reaction was heated at 100 ºC for 1 h using a microwave. The mixture was then quenched to pH7 with sat. aq. NaHCO3. The aqueous layer was extracted with EtOAc (3 × 10 mL). The organic layers were combined and washed with brine (10 mL). The organic layer was dried with MgSO4, the solvent removed in vacuo and the crude was purified with flash chromatography to give the title compound as a yellow solid (8 mg, 0.03 mmol, 11% yield).

^{1}H NMR (500 MHz, DMSO) δ 11.04 (s, 1H), 7.46 (dd, J = 7.0, 8.5 Hz, 1H), 7.00 (dd, J = 7.7, 9.8 Hz, 2H), 6.48 (s, 2H), 5.03 (dd, J = 5.5, 12.7 Hz, 1H), 2.93 – 2.81 (m, 1H), 2.62 – 2.49 (m, 2H), 2.06 – 1,97 (m, 1H). **^{13}C NMR (126 MHz, DMSO)** 172.90, 170.21, 168.72, 167.50, 146.87, 135.59, 132.15, 121.85, 111.13

Compound 20

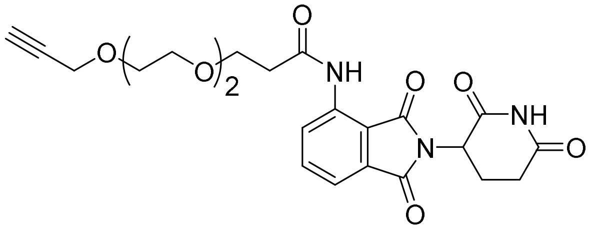

Compound **19** (500 mg, 1.8 mmol, 1.0 eq.) was dissolved in DCM:TFA (3:1) (5 mL) and stirred for 2 h at r.t. The solvent was removed *in vacuo*. The intermediate was dissolved in $SOCl_2$ (3 mL) and refluxed for 1 h. The solvent was removed and the intermediate was dissolved in dry THF (3 mL). To this pomalidomide (546 mg, 2.0 mmol, 1.1 eq.) and pyridine (142 mg, 1.8 mmol, 1.0 eq.) in dry THF (10 mL)

was added. The reaction was refluxed overnight. The reaction was quenched with H_2O (20 mL) followed by extraction with EtOAc (3 x 30 mL). The organics were combined dried with $MgSO_4$ and concentrated *in vacuo*. The crude was purified via flash chromatography to give the title compound as a bright yellow solid. (427 mg, 1.0 mmol, 56% yield).

^{1}H NMR (500 MHz, $CDCl_3$) δ 9.87 (s, 1H), 8.84 (dd, *J* = 8.5, 0.8 Hz, 1H), 8.41 (s, 1H), 7.70 (dd, *J* = 8.5, 7.3 Hz, 1H), 7.54 (dd, *J* = 7.3, 0.8 Hz, 1H), 4.98 – 4.90 (m, 1H), 4.25 – 4.11 (m, 2H), 3.88 – 3.59 (m, 10H), 2.98 – 2.69 (m, 5H), 2.41 (t, *J* = 2.4 Hz, 1H), 2.20 – 2.12 (m, 1H). **^{13}C NMR (126 MHz, $CDCl_3$) δ** 171.00, 168.71, 168.07, 166.91, 137.76, 136.38, 131.41, 125.84, 118.60, 115.83, 79.75, 74.74, 70.74, 70.48, 69.29, 66.72, 58.48, 49.36, 38.76, 31.49, 22.86.

Compound 21

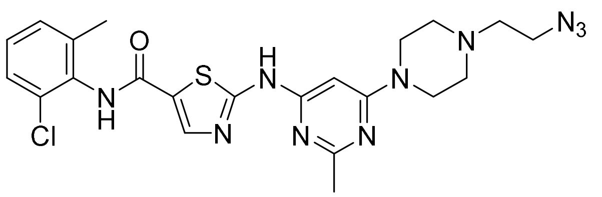

Dasatinib **16** (200 mg, 0.4 mmol, 1 eq) was sealed in a 10 ml heat-dried microwave vial and dissolved in 8 ml dry DMF under anhydrous conditions. The vial was placed in an ice bath and TEA (114 µL, 0.8 mmol, 2 eq) added dropwise, followed by addition of methane sulfonyl chloride (48 µL, 0.6 mmol, 1.5 eq). The reaction was stirred during 48 h. NaN_3 (120 mg, 1.8 mmol, 4.5 eq) was then added and the mixture stirred overnight at 50 ºC. The reaction mixture was quenched with brine (25 mL). The aqueous phase was extracted with DCM (70 mL × 3) and the organic phases were combined and dried with $MgSO_4$. The solvent was removed *in vacuo* and the crude was purified with flash chromatography to give the title compound as a white solid (144 mg, 0.28 mmol, 70% yield).

^{1}H NMR (500 MHz, DMSO) δ 11.46 (s, 1H), 9.86 (s, 1H), 8.22 (s, 1H), 7.40 (dd, J = 7.7, 1.3 Hz), 7.34 – 7.18 (m, 2H), 6.06 (s, 1H), 3.68 – 3.48 (m, 4H), 3.43 – 3.37 (m, 2H), 3.32 (s, 2H), 2.63 – 2.50 (m, 4H), 2.41 (s, 3H, CH_3), 2.24 (s, 3H). **^{13}C NMR (126 MHz, DMSO) δ** 165.17, 162.54, 162.36, 159.90, 156.95, 140.80, 138.80, 133.51, 132.42, 129.00, 128.15, 126.99, 125.70, 82.67, 56.56, 52.04, 47.11, 43.60, 25.56, 18.28.

Compound 22a

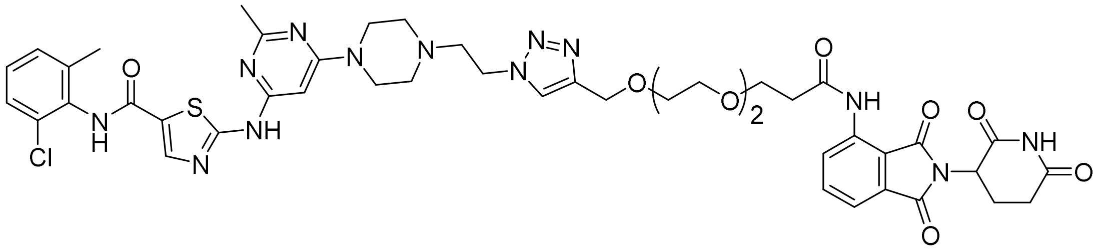

Prepared according to general procedure A, affording the title compound as a yellow solid (34 mg, 0.035 mmol, 86% yield).

^{1}H NMR (500 MHz, DMSO) δ 11.43 (s, 1H), 11.13 (s, 1H), 9.86 (s, 2H), 8.54 (d, *J* = 8.4 Hz, 1H), 8.22 (s, 1H), 8.08 (s, 1H), 7.81 (dd, *J* = 8.5, 7.3 Hz, 1H), 7.60 (d, *J* = 7.3 Hz, 1H), 7.39 (d, *J* = 7.5 Hz, 1H), 7.32 – 7.22 (m, 2H), 6.05 (s, 1H), 5.14 (dd, *J* = 12.8, 5.4 Hz, 1H), 4.51 – 4.47 (m, 4H), 3.73 (t, *J* = 6.0 Hz, 2H), 3.60 – 3.45 (m, 12H), 3.31 (s, 4H), 2.90 (ddd, *J* = 16.9, 13.9, 5.4 Hz, 1H), 2.79 (t, *J* = 6.4 Hz, 2H), 2.69 (t, *J* = 6.0 Hz, 2H), 2.64 – 2.52 (m, 2H), 2.40 (s, 3H), 2.24 (s, 3H), 2.11 – 2.03 (m, 1H).**^{13}C NMR (126 MHz, DMSO) δ** 172.70, 170.37, 169.73, 167.66, 166.63, 165.15, 162.53, 162.32, 159.89, 156.93, 143.67, 140.80, 138.80, 136.45, 136.10, 133.52, 132.42, 131.41, 128.99, 128.13, 126.97, 125.94, 125.70, 124.18, 118.21, 116.61, 82.64, 69.72, 69.66, 69.54, 68.84, 66.09, 63.49, 56.88, 51.91, 48.90, 46.61, 43.50, 37.52, 30.89, 25.52, 21.95, 18.26.

Compound 22b

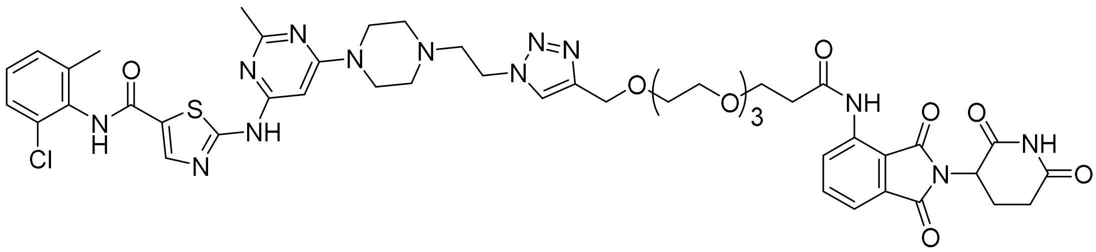

Prepared according to general procedure A, affording the title compound as a yellow solid (32 mg, 0.03 mmol, 78% yield).

^{1}H NMR (500 MHz, DMSO) δ 11.44 (s, 1H), 11.14 (s, 1H), 9.86 (s, 2H), 8.54 (d, *J* = 8.4 Hz, 1H), 8.22 (s, 1H), 8.10 (s, 1H), 7.82 (dd, *J* = 8.5, 7.3 Hz, 1H), 7.60 (d, *J* = 7.2 Hz, 1H), 7.40 (dd, *J* = 7.7, 1.8 Hz, 1H), 7.34 – 7.20 (m, 2H), 6.05 (s, 1H), 5.14 (dd, *J* = 12.8, 5.4 Hz, 1H), 4.51 (d, *J* = 3.0 Hz, 4H), 3.73 (t, *J* = 6.0 Hz, 2H), 3.60 – 3.42 (m, 16H), 3.32 (s, 4H), 2.95 – 2.84 (m, 1H), 2.79 (t, *J* = 6.4 Hz, 2H), 2.69 (t, *J* = 6.0 Hz, 2H), 2.66 – 2.51 (m, 2H), 2.40 (s, 3H), 2.24 (s, 3H), 2.07 (m, 1H).**^{13}C NMR (126 MHz, DMSO) δ** 172.73, 170.41, 169.76, 167.67, 166.66, 165.18, 162.54, 162.34, 159.91, 156.95, 143.69,

140.82, 138.82, 136.48, 136.13, 133.52, 132.43, 131.43, 129.02, 128.16, 127.00, 125.96, 125.71, 124.24, 118.24, 116.61, 82.66, 69.73, 69.64, 69.54, 68.87, 66.10, 63.52, 56.91, 51.93, 48.91, 46.61, 43.50, 37.53, 30.91, 25.55, 21.97, 18.28.

Compound 22c

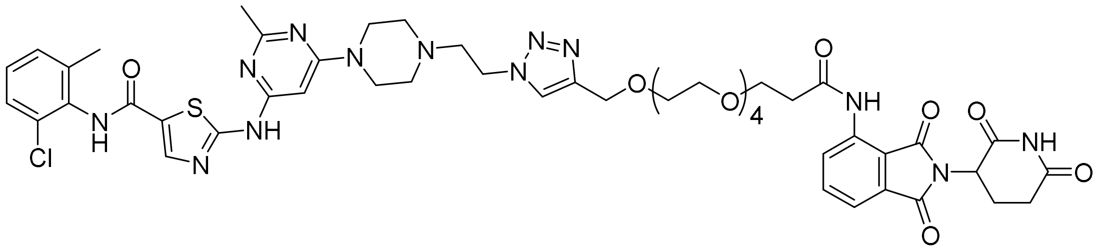

Prepared according to general procedure A, affording the title compound as a yellow solid (30 mg, 0.028 mmol, 70% yield).

^{1}H NMR (500 MHz, DMSO-d_6) δ 11.44 (s, 1H), 11.14 (s, 1H), 9.87 (s, 2H), 8.55 (d, *J* = 8.4 Hz, 1H), 8.22 (s, 1H), 8.10 (s, 1H), 7.86 – 7.79 (m, 1H), 7.60 (d, J = 7.3 Hz, 1H), 7.40 (d, J = 6.7 Hz, 1H), 7.33 – 7.22 (m, 2H), 6.05 (s, 1H), 5.14 (dd, J = 12.8, 5.4 Hz, 1H), 4.51 (d, J = 8.4 Hz, 4H), 3.73 (t, J = 6.0 Hz, 2H), 3.60 – 3.40 (m, 20H), 3.32 (s, 4H), 2.90 (ddd, J = 17.0, 13.8, 5.4 Hz, 1H), 2.79 (t, J = 6.3 Hz, 2H), 2.70 (t, *J* = 6.0 Hz, 2H), 2.65 – 2.52 (m, 2H), 2.40 (s, 3H), 2.24 (s, 3H), 2.07 (m, 1H).**^{13}C NMR (126 MHz, DMSO) δ** 172.73, 170.42, 169.76, 167.67, 166.66, 165.18, 162.55, 162.35, 159.91, 156.95, 143.69, 140.82, 138.82, 136.48, 136.14, 133.52, 132.44, 131.44, 129.02, 128.16, 127.00, 125.96, 125.71, 124.26, 118.24, 116.62, 82.66, 69.73, 69.66, 69.54, 68.88, 66.10, 63.53, 56.91, 51.93, 48.91, 46.61, 43.51, 40.0, 37.53, 30.91, 25.55, 21.97, 18.28.

6. Biological assays

The compositions of commonly used buffers are listed below. Buffers were prepared using Milli-Q® water, unless stated otherwise.

Buffer	Composition
Blocking Buffer	5% (w/v) bovine serum albumin (BSA) (Sigma, A9647) added to TBST 0.1%.
Phosphate Buffered Saline (PBS)	137 mM NaCl, 2.7 mM KCl, 10 mM Na_2HPO_4, 1.8 mM KH_2PO_4, pH 7.4.
Protease/Phosphatase inhibitors (PI)	1.25 mM PMSF (Sigma, 93482), 0.1% (v/v) aprotinin (Sigma, A6278), 100 µM Na3VO4 (Sigma, S6508), 500 µM NaF (Sigma, S7920).<br>Added to RIPA buffer.
Radioimmunoprecipitation assay (RIPA) buffer	25 mM Tris•HCl pH 7.6, 150 mM NaCl, 1% (v/v) NP-40, 1% (w/v) sodium deoxycholate, 0.1% (v/v) SDS (ThermoFischer, 89900)
RIPA lysis buffer	PI are added to RIPA buffer directly before use.
Tris Buffered Saline (TBS)	24.23 g Trizma HCl and 80.06 g NaCl in 1 L water, pH 7.6.
Tris Buffered Saline Tween (TBST 0.1%)	Add 0.1% (v/v) Tween® 20 (Sigma, P1379) to TBS.
Tris Glycine SDS (TGS)	25 mM Tris, 192 mM glycine, 0.1% (w/v) SDS, pH 8.3.
Trypsin	10X Trypsin-EDTA (Sigma, T4174) diluted to 1X in PE (PBS with EDTA) buffer.

Antibodies for Western blots were diluted in blocking buffer to which sodium azide (0.02% w/v) was added and prepared antibody dilutions were stored at 4°C.

Target	Host	Company	ID	Dilution
ABL	Mouse	SCB	SC-23	1/1000
GAPDH	Rabbit	CDT	5174	1/1000

Cell Culture Methods

MDA-MB-231 cell lines were grown in Dulbecco's Modified Eagle's Medium (Gibco, 21969035) supplemented with 10% (v/v) foetal bovine serum (Gibco, 10270-106) and 2mM L-glutamine (Gibco, 25030081). Cells were maintained in a Heracell 240i tissue culture incubator (37°C, humidified air, 5% CO_2). Cell lines in culture were mycoplasma tested every 2 months.

Cell viability assays

On day 0, cells were seeded in 96-well plates at 1000 cells per well density. Edge wells are not used due to evaporation effects affecting cell growth in these wells. After 48 hours, the medium was removed and 95 µL of fresh medium was added. Dilution plates of the test compounds were prepared in DMSO and diluted 1:50 into medium, of which 5 µL were added to the cell plates (final DMSO concentration 0.1% v/v). Cells were treated for 5 days before cell viability was measured using the resazurin-based PrestoBlue solution (Invitrogen, A13262), which becomes fluorescent red in the reducing environment of viable cells. PrestoBlue (10% v/v) was added to treated plates on day 7 and fluorescence (550 nm excitation, 580 nm emission) was measured 90 min after addition, using the Perkin Elmer Envision 2101 plate reader. All conditions were normalized to the untreated cells (100%) and blank wells containing just media and PrestoBlue (10%). GraphPad Prism 7 was used to fit non-linear regression curves (four-parameter logistic fit). Experiments were performed in triplicate.

Biocompatibility study

The toxicity of the Cu microdevices were tested by performing viability studies on MDA-MB-231 cells. Cells were plated as indicated above. Each well was then replaced with 100 µL of fresh media containing Cu microdevices at 0.1, 0.5, 1.0, 1.5 or 2.0 mg/mL. PrestoBlue (10% v/v) was added to treated plates on day 7 and fluorescence (λex/em = 550/580 nm) was measured 90 min after addition, using the Perkin Elmer Envision 2101 plate reader. All conditions were normalized to the untreated cells (100%) and blank wells containing just media and PrestoBlue (10%). GraphPad Prism 7 was used to fit non-linear regression curves (four-parameter logistic fit). Experiments were performed in triplicate.

Cell lysates

Cells were seeded in 6-well plates and incubated for 48 h until 70% confluence was reached. Compounds were added (final [DMSO] 0.1% v/v) and cells incubated for the time specified (3 or 24 h). Plates were put on ice, the medium was removed and cells were washed twice with ice-cold PBS. RIPA lysis buffer with protease and phosphatase inhibitors was added (100 µL / well) and the cells were scraped, collected and incubated on ice for 10 min with occasional vortexing. Lysates were centrifuged at 4°C (17,000 x g, 10 min) and the supernatant was transferred into a new microcentrifuge tube. Lysates were stored at -20°C (short-term) or -70°C (long-term). The protein concentration of lysates was determined in a BCA assay. The Pierce BCA Protein Assay Kit (Thermo Scientific, 23225) was used according to the manufacturer's instructions, with samples being diluted 1 in 5 and set up in duplicate on 96-well plates. Plates were read on a Spark 20M microplate reader (Tecan) and absorbance was measured at 562 nm.

Western Blotting

Samples for SDS-PAGE were prepared as follows: 20 µg cell lysate was diluted with RIPA buffer to a final volume of 15 µL, to which 5 µL of 4X Laemmli Sample Buffer (Bio-Rad, 1610747) were added. Samples were heated to 95°C for 5 min and then loaded onto Mini-PROTEAN TGX precast gels (12-well or 15 well, 4-15% or 12%, depending on experiment; Bio-Rad). Precision Plus Protein Dual Color Standards (Bio-Rad, 1610374) were loaded alongside the lysates. Gels were run in Mini-PROTEAN Tetra Cell tanks in TGS buffer (pH 8.3) at 150 V for 45 min. Proteins were transferred onto Trans-Blot Turbo Nitrocellulose membranes (Bio-Rad, 1704159) using the Trans-Blot Turbo Transfer System (Bio-Rad) for semi-dry blotting at 2.5 A (midi) or 1.3 A (mini) and up to 25 V for 10 min. Membranes were cut and blocked with blocking buffer for 1 h at rt before incubation with primary antibodies at 4°C overnight. Membranes were washed twice with TBST (0.1%) for 3 minutes before addition of secondary antibodies and incubation for 2 h at rt, before washing another 3 times with TBST (0.1%) for 5 min. Antibodies were detected by chemiluminescence by incubating membranes for 2 min with the Clarity ECL Western Blotting Substrates (Bio-Rad, 102031309) before imaging in the ChemiDoc XRS+ Imaging System. Protein bands were quantified using the ImageLab software v5.2 with automated band detection and background subtraction (disk size 70 mm). Experiments were performed in triplicate.

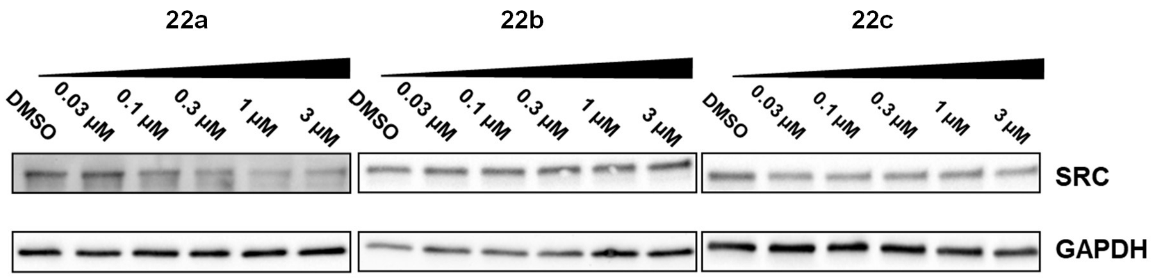

Figure S2. Study of SRC protein degradation by 22a-c in MDA-MB-231 cells. Cells were treated for 24 h prior to cell lysis and analysis by Western blot. Representative Western blot of MDA-MB-231 cells of two biological replicates.

Click assay

A solution containing the desired concentration of **14a** (1 mg/mL), 100 µM of azido derivative of dasatinib, **21**, and 100 µM of CRBN ligand that contained a terminal alkyne, **20a**, was prepared in an Eppendorf tube in H_2O:MeOH (7:3). The mixtures were shaken at 700 rpm and 37 °C in a Thermomixer for 48 h. At timepoints 2, 4, 24, 48 h the supernatant was analyzed by LC/MS (Agilent 1260 Infinity II) using a DAD detector (254 nm) with gradient A.

Click assay in presence of sodium ascorbate

A solution containing the desired concentration of **14a** (1 mg/mL), 100 µM of azido derivative of dasatinib, **21**, 100 µM of CRBN ligand that contained a terminal alkyne, **20**, and sodium ascorbate (0.0, 12.5, 25.0, 50.0, 100.0, 150.0 µM) was prepared in an Eppendorf tube in H_2O:MeOH (7:3). The mixtures were shaken at 700 rpm and 37 °C in a Thermomixer for 2 h. The supernatant was analyzed by LC/MS (Agilent 1260 Infinity II) using a DAD detector (254 nm) with gradient A.

2 h

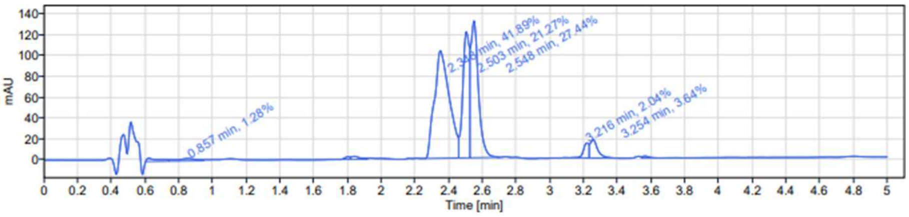

4 h

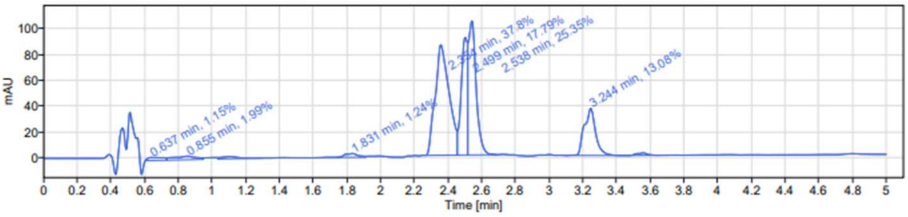

24 h

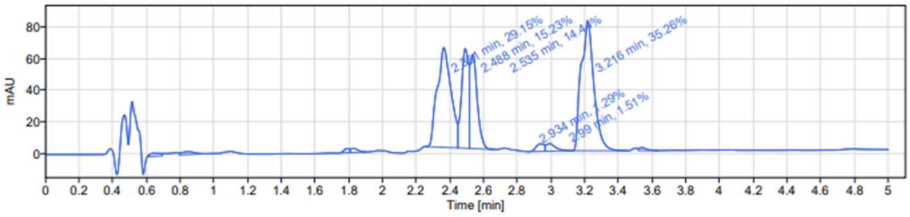

48 h

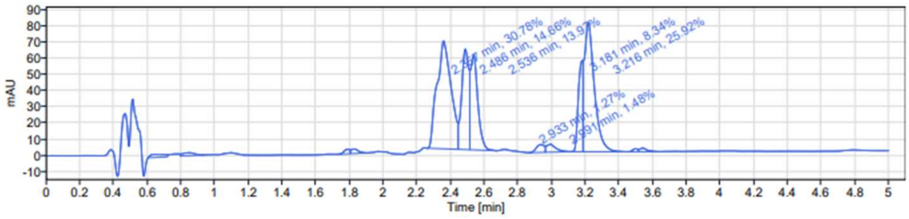

Figure S3. In vitro synthesis of PROTAC without NaAsc. Representative HPLC chromatograms of the reaction at various timepoints. Reaction conditions: **20a** (100 µM), **21** (100 µM) and Cu(**I**)-microdevices (1 mg/mL) were incubated in H_2O:MeOH (70:30) at 37°C in a thermomixer.

In situ PROTAC assembly in cancer cell culture.

Cells were seeded in 6-well plates and incubated for 48 h until 70% confluence was reached. MDA-MB-231 were treated with **20c** (0.25 µM), **21** (0.25 µM), Cu(**I**)- microdevices (1 mg/ml) and NaAsc (150 µM) for 48 h prior to cell lysis and analysis by Western blot. Negative controls lack one or more of the key components. **22a** (0.25 µM) only was used as positive control.

7. Literature references:

(1) Ghosh, P. B.; Whitehouse, M. W. Potential Antileukemic and Immunosuppressive Drugs. Preparation and in Vitro Pharmacological Activity of Some 2,1,3-Benzoxadiazoles (Benzofurazans) and Their N-Oxides (Benzofuroxans). *J. Med. Chem.* **1968**, *11* (2), 305–311. https://doi.org/10.1021/jm00308a027.

(2) Bertrand, P.; Gesson, J. P. Click Chemistry with *O* -Dimethylpropargylcarbamate for Preparation of pH-Sensitive Functional Groups. A Case Study. *J. Org. Chem.* **2007**, *72* (9), 3596–3599. https://doi.org/10.1021/jo070131j.

(3) Ortega-Liebana, M. C.; Porter, N. J.; Adam, C.; Valero, T.; Hamilton, L.; Sieger, D.; Becker, C. G.; Unciti-Broceta, A. Truly-Biocompatible Gold Catalysis Enables Vivo-Orthogonal Intra-CNS Release of Anxiolytics. *Angew. Chem. Int. Ed.* **2022**, *61* (1). https://doi.org/10.1002/anie.202111461.

(4) L'abbé, G.; Bruynseels, M.; Delbeke, P.; Toppet, S. Molecular Rearrangements of 4-Iminomethyl-1,2,3-Triazoles. Replacement of 1-Aryl Substituents in 1 *H* -1,2,3-Triazole-4-Carbaldehydes. *J. Heterocycl. Chem.* **1990**, *27* (7), 2021–2027. https://doi.org/10.1002/jhet.5570270733.

(5) Chan, T. R.; Fokin, V. V. Polymer-Supported Copper(I) Catalysts for the Experimentally Simplified Azide–Alkyne Cycloaddition. *QSAR Comb. Sci.* **2007**, *26* (11–12), 1274–1279. https://doi.org/10.1002/qsar.200740131.

(6) Soriano del Amo, D.; Wang, W.; Jiang, H.; Besanceney, C.; Yan, A. C.; Levy, M.; Liu, Y.; Marlow, F. L.; Wu, P. Biocompatible Copper(I) Catalysts for in Vivo Imaging of Glycans. *J. Am. Chem. Soc.* **2010**, *132* (47), 16893–16899. https://doi.org/10.1021/ja106553e.

(7) Malkoch, M.; Schleicher, K.; Drockenmuller, E.; Hawker, C. J.; Russell, T. P.; Wu, P.; Fokin, V. V. Structurally Diverse Dendritic Libraries: A Highly Efficient Functionalization Approach Using Click Chemistry. *Macromolecules* **2005**, *38* (9), 3663–3678. https://doi.org/10.1021/ma047657f.

(8) Ursuegui, S.; Schneider, J. P.; Imbs, C.; Lauvoisard, F.; Dudek, M.; Mosser, M.; Wagner, A. Expedient Synthesis of Trifunctional Oligoethyleneglycol-Amine Linkers and Their Use in the Preparation of PEG-Based Branched Platforms. *Org. Biomol. Chem.* **2018**, *16* (44), 8579–8584. https://doi.org/10.1039/C8OB02097C.

(9) Edem, P. E.; Czorny, S.; Valliant, J. F. Synthesis and Evaluation of Radioiodinated Acyloxymethyl Ketones as Activity-Based Probes for Cathepsin B. *J. Med. Chem.* **2014**, *57* (22), 9564–9577. https://doi.org/10.1021/jm501357r.
